# Smoking cessation intervention for Indigenous pregnant women: A systematic review of randomized controlled trials

**DOI:** 10.1016/j.pmedr.2026.103479

**Published:** 2026-04-17

**Authors:** Dan Yedu Quansah, Salma Mahmoodianfard, Javad Heshmati, Sarah Visintini, Meagan Noble, Ekua Amponsah Agyemang, Kerri-Anne Mullen, Hassan Mir

**Affiliations:** a Canadian Women's Heart Health Centre, University of Ottawa Heart Institute, Ottawa, Canada; b School of Epidemiology and Public Health, University of Ottawa, Ottawa, Canada; c Division of Cardiology, Department of Medicine, University of Ottawa Heart Institute, University of Ottawa, Ottawa, Canada; d Berkman Library, University of Ottawa Heart Institute, Ottawa, Canada; e Northern Ontario School of Medicine University, Canada; f Faculty of Medicine and Dentistry, Department of Public health and Preventive Medicine, University of Alberta, Canada; g Faculty of Medicine, University of Ottawa, Ottawa, Canada

**Keywords:** Smoking, Cessation, Indigenous, Pregnant, Women, Interventions

## Abstract

**Objective:**

To synthesize evidence on smoking cessation interventions among pregnant Indigenous women.

**Methods:**

We conducted a systematic search of databases from thier inception (MEDLINE, 1946; Embase, 1947; CENTRAL, 1991; APA PsycINFO, 1806; Informit Indigenous Collection, 1977; Bibliography of Indigenous Peoples in North America, 1900; and Global Health, 1973) to May 28, 2025. Eligible studies included trials targeting smoking or vaping cessation among pregnant Indigenous women. Interventions at the individual or community level were eligible, including education, counseling, pharmacotherapy, incentives, biomarker feedback, digital health, or community-based programs.

**Results:**

Of 5534 studies screened, five studies involving 723 pregnant Indigenous women from Australia, New Zealand, and the United States met inclusion. Validated cessation rates were low, rarely exceeding 20% across interventions. Three studies reported improvements in quit attempts and quit-date setting. All interventions were feasible and acceptable, particularly those embedded within Indigenous health services. Facilitators included Indigenous leadership, provider training, and use of culturally relevant materials, whereas low recruitment (32%) and retention rates (<40%), staff turnover, and variable intervention fidelity were identified as barriers to intervention implementation.

**Conclusions:**

Evidence suggests that although culturally adapted smoking cessation interventions are feasible, they have not consistently improved abstinence rates among pregnant Indigenous women, reflecting broader structural and contextual barriers.

## Highlights

• Five trials have evaluated cessation interventions in pregnant Indigenous women.
• Culturally adapted interventions improved quit attempts but not sustained abstinence.
• High attrition and implementation challenges limited intervention effectiveness.
• Evidence gaps persist for pregnant Indigenous populations in Canada and the United States.
• System and community-level approaches are needed to address structural barriers.

## 1 Introduction

Tobacco use in pregnancy is a significant modifiable risk factor for adverse maternal and infant health outcomes, including intrauterine growth restriction, preterm birth, low birthweight, and sudden infant death syndrome (Tarasi et al., 2022; Avşar et al., 2021). Despite substantial public health efforts, smoking during pregnancy remains disproportionately higher among Indigenous women compared to non-Indigenous women (Gould et al., 2017a). In Australia, 44% to 52% of Aboriginal and Torres Strait Islander women report smoking during pregnancy compared to 12% to 15% of non-Indigenous women (Eades et al., 2012). In New Zealand, 34% of pregnant Māori women smoked in 2007 compared to 11% of all New Zealand women, and among Indigenous peoples of Alaska, smoking prevalence during pregnancy is nearly three times higher than in non-Indigenous women (36% vs. 13%) (Glover et al., 2015a). These inequities mirror patterns observed among First Nations women in Canada, where smoking rates exceed 60% in some communities compared to less than 30% in non-Indigenous populations (Heaman and Chalmers, 2005; Public Health Agency of Canada, 2009). The pattern of adverse perinatal health outcomes among Indigenous women due to smoking are shaped by complex, interrelated factors, including systemic racism, colonialism, socioeconomic disadvantage, psychosocial stress, and limited access to culturally safe cessation services (Gould et al., 2017b; Ramsoondar et al., 2023). It is important to acknowledge that tobacco holds cultural and spiritual significance for many Indigenous Peoples, where it is traditionally used as a sacred medicine in ceremony and healing. However, harms related to the use of commercial tobacco, differs fundamentally in purpose, preparation, and meaning.

Indigenous women frequently report barriers to participation in mainstream cessation programs, including judgmental interactions with healthcare providers, lack of culturally appropriate resources, and inadequate integration of family or community supports (Wood et al., 2008). Other barriers include receiving inconsistent information that smoking cessation during pregnancy is safe, perception that nicotine replacement therapy was unsafe in pregnancy, barriers that prevent attendance to prenatal appointments (Salgin et al., 2025). As a result, conventional approaches such as brief advice, pharmacotherapy, and general counseling often achieve limited effectiveness in Indigenous populations (Coleman et al., 2015). Evidence suggests that tailored, community-driven interventions may improve engagement and outcomes. Systematic reviews of smoking cessation during pregnancy show that financial incentives are among the most effective strategies, increasing quit rates by two- to three-fold in general populations (Notley et al., 2019; Tappin et al., 2025). However, few studies have evaluated these or other intensive approaches among Indigenous women. The available evidence is inconsistent, with studies highlighting both the promise and the challenges of culturally tailored smoking cessation interventions for pregnant Indigenous women. While feasibility and acceptability have been demonstrated, consistent improvements in biochemically verified (refers to the objective confirmation of smoking status using biomarkers such as exhaled carbon monoxide (CO) or cotinine levels) quit rates remain unsuccessful (Notley et al., 2019). As such, synthesizing available evidence is critical to inform future interventions. This review summarized findings from randomized controlled trials (RCTs) that evaluated smoking cessation interventions for pregnant Indigenous women, with particular attention to effectiveness, behavioral outcomes, feasibility, cultural acceptability and implementation.

## 2 Methods

The protocol of this review is registered on PROSPERO (CRD420250591378). This review followed the PRISMA guidelines, consistent with the reporting of systematic reviews.

### 2.1 Search strategy and study selection

We conducted a systematic review of the literature to identify RCTs that evaluated smoking cessation interventions among Indigenous populations globally. A peer-reviewed search strategy was conducted in June and September of 2024 in MEDLINE, Embase, CENTRAL, and APA PsycINFO, Informit Indigenous Collection, Bibliography of Indigenous Peoples in North America, and Global Health (McGowan et al., 2016). All databases were searched from the date of inception (MEDLINE, 1946; Embase, 1947; CENTRAL, 1991; APA PsycINFO, 1806; Global Health, 1973, Informit Indigenous Collection, 1977; and Bibliography of Indigenous Peoples in North America, 1900). Only studies published in English were included. A search update was conducted May 28, 2025, except for Informit Indigenous Collection, as it was initially searched as part of a trial, was difficult to export from, and the original search did not yield any relevant studies. The searches were undertaken using combinations of keywords related to global Indigenous peoples and tobacco use cessation and was informed by previously conducted systematic searches and search filters (see supplementary file). Search results were exported to Covidence (Melbourne, Australia) and duplicates were eliminated using the platform's duplicate identification feature. Titles and abstracts were screened independently by two reviewers. Full-text articles were reviewed by two reviewers against the eligibility criteria. At all times the two reviewers reached consensus on study inclusion through discussion; overall agreement during screening exceeded 90%.

### 2.2 Eligibility criteria

Studies were eligible if they were intervention trials and evaluated smoking cessation intervention delivered to Indigenous individuals or communities with the aim of reducing or stopping tobacco or nicotine use during pregnancy. Indigenous people included Aboriginal, First Nations, Inuit, Métis, Māori, Indigenous people of Alaska, and Indigenous people from American communities. Participants were currently using commercial tobacco or e-cigarettes, or recent quitters at risk of relapse. Interventions implemented at either the individual or community level, involving education programs, counseling, pharmacotherapy, incentive-based approaches, biomarker feedback, digital health tools, or other culturally adapted initiatives were included. Studies were excluded if they were case reports, case series, observational studies, registry analyses, or narrative or systematic reviews.

The primary outcomes of interest were cessation or reduction in smoking or vaping, as determined by self-report or biochemical validation (i.e., CO-confirmed status). Secondary outcomes included perceived cultural safety or acceptability, changes in the intention to quit, and measures of behavior change, such as quit attempts or readiness to quit.

### 2.3 Data extraction and synthesis

Data from each eligible study were extracted based on the study design, setting, population, sample size, intervention content and delivery, follow-up duration, recruitment and retention rates, and reported outcomes. Where available, effect estimates, and associated *p*-values were recorded if available. Given that the included studies varied substantially in study design, interventions, and outcome reporting, we could not pool results quantitatively and meta-analysis was not feasible. Instead, results were synthesized narratively. Findings are presented descriptively, with emphasis on intervention effectiveness, behavioral outcomes, feasibility, and cultural acceptability. For this review, we distinguish between culturally adapted and culturally grounded interventions. Culturally adapted interventions referred to those modified to align with Indigenous populations through tailored materials, language, or delivery approaches. Culturally grounded interventions are rooted in Indigenous values and knowledge systems, emphasizing relationship-centered approaches involving family, community, and self.

Terms such as culturally safe, culturally targeted, culturally relevant, culturally tailored, culturally adapted, and culturally grounded are used variably in literature and reflect different levels of cultural integration. In this review, these terms are not treated as interchangeable. We aim to distinguish between surface-level adaptations and deeper, culturally grounded approaches rooted in Indigenous values, relationships, and knowledge systems.

### 2.4 Risk of bias assessment

Risk of bias for the included RCTs was assessed independently using a 12-item checklist adapted from the Cochrane Risk of Bias 2.0 framework. The checklist evaluated methodological domains, including randomization, allocation concealment, blinding, handling of missing data, selective reporting, and appropriateness of the statistical analysis. Each criterion was rated as *Yes (Y = 1)*, *Unclear (U = 0.5)*, or *No (N = 0)*. The total score for each study was calculated by summing across all items and converted into an overall risk category, defined as low risk (≥ 9 points, equivalent to ≥75% of criteria met), moderate risk (6–8.5 points, 50–74%), or high risk (< 6 points, < 50%).

## 3 Results

### 3.1 Study selection and characteristics

**Fig. 1:**
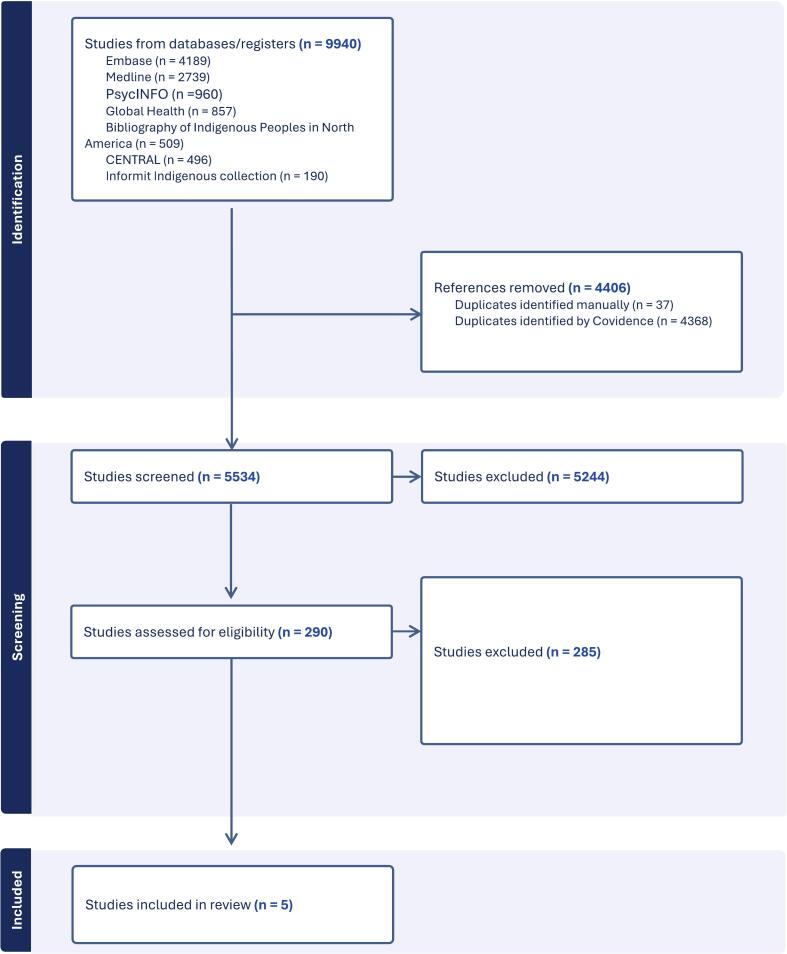
Flow chart of study selection for randomized controlled trials of smoking cessation interventions in Indigenous pregnant women through database searches up to May 2025.

Out of 5534 eligible studies screened, five studies met our inclusion criteria and were included in this review (Supplemental File, References S1–S5) (Fig. 1). These studies enrolled a total of 723 pregnant Indigenous women from Australia, New Zealand, and the United States (Alaska). Study designs included two RCTs (S1–S2) (Eades et al., 2012; Patten et al., 2019), one feasibility RCT (S3) (Gould et al., 2019), a pilot cluster step-wedge trial (S4) (Glover et al., 2015b) and one community cluster RCT (S5) (Patten et al., 2020). All interventions included elements of cultural adaptation; however, the degree of Indigenous leadership varied. Interventions by Gould et al. and Patten et al. (2020) were co-designed with Indigenous communities or delivered within community-controlled settings. In contrast, those by Eades et al. and Patten et al. (2019) were implemented within Indigenous-serving health services - such as Aboriginal Community Controlled Health Services in Australia, Tribal Health Organizations or the Indian Health Service in the United States, but did not explicitly report Indigenous governance. The study by Glover et al., which targeted Māori women, also did not clearly describe the extent of Indigenous leadership. Overall, comparators were usual care or standard smoking cessation counseling. Table 1 shows the detailed characteristics of the included studies. Although vaping was included in the eligibility criteria, none of the included studies reported vaping outcomes separately, and no vaping-specific analyses were feasible.

**Table 1:** Summary of randomized controlled trials of smoking cessation intervention among Indigenous pregnant women (Australia, New Zealand, and the United States, 2012–2020) included in this systematic review.

Author, year and country	Study objective	Study Design and population	Intervention description and duration	Major findings	Conclusions
**Eades et al., 2012 (****Patten et al., 2019****), Australia**	To determine the effectiveness of an intensive quit smoking intervention on smoking rates at 36 weeks' gestation among pregnant Aboriginal and Torres Strait Islander women.	***Design:*** randomized controlled trial ***Population:*** 263 Aboriginal and Torres Strait Islander pregnant women (148 in intervention and 115 in control group) ***Age:*** Mean age of 24 years	***Intervention (N*** ***=*** ***148):*** Intensive smoking cessation intervention (case management, counseling, resources, nicotine replacement therapy access) ***Usual care (N-115):*** General advice about quitting smoking, based on guidelines **Duration:** Pregnancy until delivery	No significant difference in cessation rates (11.40% vs 9.60%); intervention feasible but implementation issues limited effect. No significant difference (*p* > 0.05); in implementation issues (staff turnover, fidelity), limited effectiveness despite feasibility.	***Culturally relevant:*** Delivered in Aboriginal Maternal and Infant Health Services; community-informed approach ***Barriers:*** Implementation problems, staff turnover, high baseline prevalence of smoking ***Facilitators:*** Community-controlled health services involvement, culturally relevant setting.

**Glover et al., 2015 (****Gould et al., 2019****), New Zealand**	To investigate if pregnant indigenous New Zealand women who smoke are more likely to abstain from smoking if given products or vouchers.	***Design:*** feasibility randomized controlled trial ***Population***: 24 Māori pregnant women, Māori women (16 in intervention groups and 8 in control group) ***Age:*** mean age of 25 years	***Intervention (N*** ***=*** ***16):*** patients were randomized to either***: 1)*** usual cessation support plus a retail voucher to the value of New Zealand $25 for each ‘abstinent from smoking’ week for eight weeks (voucher) or 2) usual cessation support plus product to the value of New Zealand $25 for each abstinent from smoking’ week for eight weeks (product). ***Usual cessation control (N*** ***=*** ***8):*** patients were provided with cessation support, including information about different cessation products and services. **Duration:** during pregnancy	Recruitment 32% (24/75 eligible), retention 37.50%. At 8 weeks, validated abstinence: 3/24 (12.50%) Voucher vs product arm: abstinence 17% vs 8% (not statistically significant, *p* > 0.05). Feasibility limited due to poor recruitment/retention; some evidence that vouchers may outperform products.	***Culturally relevant:*** Targeted to Māori women, delivered in culturally relevant settings ***Barriers:*** Low recruitment/retention, stigma, tone of trial promotion affected engagement ***Facilitators:*** Use of incentives, cultural adaptation

**Gould et al., 2018 (****Glover et al., 2015b****), Australia**	To explore the feasibility and acceptability of a) a co-designed multi-component intervention for healthcare providers at AMSs in culturally targeted pregnancy specific smoking cessation care and b) the study design.	***Design:*** pilot cluster randomized step-wedge trial ***Population***: 22 pregnant women; 50 health providers, Aboriginal and Torres Strait Islander women ***Age:*** ≥16 years	***Intervention***: ICAN QUIT in Pregnancy – multi-component intervention (provider training, patient resources, carbon monoxide monitoring, oral nicotine replacement therapy) ***Usual care:*** Not applicable **Duration:** 12 weeks	Quit attempts 40.90% (9/22); biochemically validated quit rate 13.60% at 12 weeks; intervention feasible and acceptable to Aboriginal medical services staff. Self-reported quit attempts significantly higher compared with baseline (*p* < 0.05); overall validated cessation rate remained modest.	***Culturally relevant:*** Co-designed with Aboriginal Advisory Panel; culturally tailored resources; delivered in Aboriginal Medical Services ***Barriers:*** Low recruitment at some sites; staff burden; lengthy surveys; step-wedge design confusion ***Facilitators:*** Cultural tailoring, community consultation, health provider training, use of NRT and CO meters, supportive resources

**Patten et al., 2019 (****Eades et al., 2012****), USA (Alaska)**	To evaluate the feasibility and potential efficacy of a SCT-based biomarker feedback intervention among pregnant Alaska Native smoker	***Design:*** randomized controlled trial ***Population***: 60 pregnant women (30 intervention, 30 control), Alaska Native women ***Age:*** mean age 26 years	***Intervention (N*** ***=*** ***30)***: Biomarker feedback intervention: Welcome letter and brochure with graphical explanation of maternal cotinine and infant 4-(methylnitrosamino)-1-(3-pyridyl)-1-butanol (tobacco lung specific carcinogen) exposure; three counseling calls using the 5A’s framework; personalized cotinine results provided; risks emphasized via motivational interviewing; social-cognitive theory strategies to reinforce risk perception, self-efficacy, and short-term goal setting. ***Usual care (N*** ***=*** ***30):*** Welcome letter and generic brochure on smoking risks in pregnancy; three counseling calls using the 5A’s framework; standard cessation advice without biomarker results or personalized feedback. **Duration:** 3 counseling calls (weeks 2–4), assessed at delivery	Quit rates were similar in both groups at delivery (20% intention to treat, 26% per protocol); intervention was feasible and acceptable but no added effect over usual care. At delivery, biochemically confirmed abstinence: 20% intention to treat vs 20% control (*p* = 1.00). Per protocol: 26% in both groups (p = 1.00). Quit date setting significantly higher in intervention vs control (87% vs 63%, *p* = 0.04). No significant changes in social-cognitive theory-based psychosocial measures.	***Culturally relevant:*** Community advisory board; culturally adapted messaging; embedded in Alaska Native health system ***Barriers:*** Message salience limited; small sample; only one geographic site; readiness to quit was already high ***Facilitators:*** Integration into existing Quit Tobacco Program; community engagement; culturally adapted biomarker feedback

**Patten et al., 2020 (****Patten et al., 2020****), USA (Alaska)**	To evaluate the effectiveness of a community-level intervention to reduce tobacco use during pregnancy and postpartum among Alaska Native women.	***Design:*** cluster RCT ***Population***: 352 pregnant (188 in intervention and 164 in control group) Alaska Native women, 16 villages (67% tobacco users at baseline; gestational age 26.8 ± 9.8 weeks) ***Age:*** mean age 25.8 ± 5.0	***Intervention (N*** ***=*** ***188):*** Community-level campaign using brochures, posters, DVDs, social media, and peer-led phone counseling (up to six sessions pre/postpartum) by trained local “Native Sisters” incorporating Yup'ik cultural teachings and 5A’s model. ***Control (N*** ***=*** ***164):*** standard prenatal counseling following 5A’s framework (Ask, Advise, Assess, Assist, Arrange), pregnancy-specific written materials, and referral to cessation services; access to state quit line and nicotine replacement therapies via Medicaid or Indian Health Service. ***Duration:*** ∼20 months, participants followed until 6 months postpartum.	No significant difference in biochemically confirmed tobacco abstinence at 6 months postpartum (85.60% vs 82.30%, *p* = 0.38). Intervention participants were more likely to make a quit attempt at 2 months postpartum (70% vs 51%, *p* = 0.01) and to use nicotine replacement therapy at delivery (7% vs 1%, *p* = 0.02). Higher exposure to intervention was associated with greater odds of abstinence at 6 months (odds ratio: 1.20; 95% confidence interval 1.01, 1.43). Overall program reach: 73% of eligible women, 55% of all pregnant women.	The community-level intervention was feasible and achieved broad community reach but did not significantly improve smoking abstinence. The program increased quit attempts and nicotine replacement therapy use in the early postpartum. ***Culturally relevant:*** Co-designed with the Yukon-Kuskokwim Health Corporation and guided by a Community Advisory Committee. ***Barriers:*** Limited access to technology, staff turnover. ***Facilitators:*** Peer-led model using trusted local strategies.

### 3.2 Intervention description

Interventions across the five included studies differed in delivery format, intensity, and behavioral components, but shared a consistent focus on cultural adaptation and Indigenous leadership. In the largest RCT, Eades et al. (Eades et al., 2012) tested an intensive smoking cessation program combining individualized counseling, case management, and access to nicotine replacement therapy (NRT) compared with usual antenatal care. In one study (Eades et al., 2012), participants received a biomarker feedback intervention, in which urinary cotinine test results were reviewed alongside a brochure, illustrating the link between maternal cotinine levels and neonatal exposure to NNAL (a tobacco-specific nitrosamine). Counselors used motivational interviewing within the 5A’s framework (Ask, Advise, Assess, Assist, Arrange) to discuss personalized biomarker results, reinforce risk awareness, and promote short-term quit goals, whereas the control group received standard cessation counseling without biomarker feedback. Glover et al., (Gould et al., 2019) evaluated an incentive-based approach among Māori pregnant women, providing retail vouchers or household products valued at NZ$25 for each week of validated abstinence over eight weeks, in addition to usual cessation support. The study conducted by Gould et al., (Glover et al., 2015b) implemented the ICAN QUIT in Pregnancy program, a multi-component intervention co-designed with Aboriginal Medical Services that included health provider training, culturally tailored patient resources, carbon monoxide (CO) monitoring, and the use of oral NRT. The Healthy Pregnancy Project trial (Patten et al., 2020) implemented a community-level intervention which integrated social marketing materials (posters, DVDs, brochures, social media) and peer-led telephone counseling by trained local “Native Sisters” grounded in Yup'ik cultural teachings and the 5A’s model.

### 3.3 Smoking cessation outcomes

Quit rates during pregnancy remained low across studies. The largest trial, Eades et al., (Patten et al., 2019) found no significant difference in biochemically confirmed abstinence at 36-week gestational age between women receiving NRT compared to usual care (11% vs 5%, *p* = 0.21). In the original report, this corresponded to an adjusted risk ratio of 0.93 (95% CI: 0.86, 1.08) for continued smoking.

Similarly, Patten et al., (Eades et al., 2012) reported no difference in biochemically confirmed abstinence at delivery between Indigenous women of Alaska randomized to biomarker feedback, plus counseling and those receiving counseling alone. Abstinence rates were identical in both groups (intention-to-treat: 20% vs. 20%; per-protocol: 26% vs. 26%; *p* = 1.00).

In the feasibility trial by Glover et al., (Gould et al., 2019) 21% of Māori women achieved at least six weeks of abstinence during the eight-week intervention, with higher quit rates in the product incentive intervention arm (38%) than the voucher (13%) or control (13%) arms. In addition, only 37.5% of Māori participants were retained over eight weeks, limiting interpretation of process outcomes, but women in the product incentive arm showed a non-significant increase in sustaining abstinence across multiple weeks (16.7% vs. 8.3% in the voucher arm; *p* > 0.05) compared to controls. Although not statistically significant (p > 0.05), there were observable absolute differences between intervention arms. In the ICAN QUIT in Pregnancy pilot trial (Patten et al., 2020), quit smoking attempts increased significantly (41% vs. baseline, *p* < 0.05), but validated abstinence was 13.6% at 12 weeks compared to baseline (Glover et al., 2015b). In a more recent community-based cluster RCT (Patten et al., 2020), a culturally grounded campaign combining social marketing and peer counseling found that although the intervention achieved broad reach, biochemically confirmed abstinence at six months postpartum did not differ significantly between groups (14.4% vs. 17.7%, *p* = 0.38). However, participants in the intervention arm were more likely to make a quit attempt at two months postpartum (70% vs. 51%, *p* = 0.012) and to use NRT at delivery (7% vs. 1%, *p* = 0.02).

Attrition was high across several studies, with follow-up completion ranging from 37% to 70%. Only two trials reported intention-to-treat (ITT) analyses, whereas the remaining studies relied primarily on per-protocol or completer analyses. The use of per-protocol analyses, together with differential attrition, may overestimate intervention effects by excluding participants lost to follow-up. Combined with inconsistent biochemical validation across studies, these factors limit the robustness of the cessation estimates.

### 3.4 Behavioral, psychosocial, and process outcomes

Secondary behavioral outcomes varied across studies. Patten et al., (Eades et al., 2012) reported that women in the biomarker feedback arm were significantly more likely to set a quit date compared with usual care (87% vs. 63%, *p* = 0.04), Although women in the biomarker feedback arm were more likely to set a quit date, no significant differences were observed in self-efficacy, motivation, or perceived risk between groups (Patten et al., 2020), which suggests that biomarker feedback may influence short-term behavioral intentions through mechanisms not captured by these measures.

Similarly, findings from the Healthy Pregnancies Project (Patten et al., 2020) showed that higher exposure to the campaign was associated with increased odds of abstinence at six months postpartum (OR: 1.20, 95% CI: 1.01, 1.43) (Patten et al., 2020).

### 3.5 Implementation outcomes and barriers

Implementation feasibility was a challenge in all included studies. High attrition (>30% lost to follow-up), staff turnover, and difficulties maintaining protocol adherence were highlighted by Eades et al., (Patten et al., 2019). Despite high willingness to attempt quitting (over 70% at baseline), Eades et al., (Patten et al., 2019) reported that intervention fidelity varied, with physicians delivering key components in about 64% of cases and much lower adherence among nurses and health workers.

The study by Glover et al., (Gould et al., 2019) faced barriers in recruitment (32% of referred women consented) and retention (38%) and suggested that future strategies may need to reduce reliance on health professional referral. The study did not report recruitment or retention stratified by self-referral versus health-professional referral. In contrast, Gould et al., (Glover et al., 2015b) emphasized the importance of provider training, cultural adaptation, and use of CO monitors to increase engagement. Although participants' engagement with intervention components was described, acceptability was not formally measured. Greater exposure to campaign elements was associated with increased smoking abstinence, suggesting a relationship between intervention engagement and effectiveness. In addition, the use of local “Native Sisters” as peer counselors and Yup'ik cultural materials facilitated engagement, although geographic isolation, limited technology access, and staff turnover were identified as barriers (Chamberlain et al., 2017). In Patten et al., (Eades et al., 2012), a biomarker feedback intervention delivered alongside motivational interviewing within the 5A’s framework was compared with standard cessation counseling without biomarker feedback; however, despite successful implementation, with high treatment adherence (80% completed all calls) and acceptability (87% recommend the program), cessation outcomes did not improve compared to standard counseling.

### 3.6 Risk of bias

A detailed domain-level assessment for each included study is presented in Table 2. Briefly, the risk of bias assessment of the included RCTs ranged from moderate to low risk of bias. Eades et al. (Patten et al., 2019) and Patten et al. (Eades et al., 2012) were assessed to be of low risk. Both studies provided clear descriptions of the randomization process, used objective biochemical verification of abstinence, and reported complete outcome data, although minor concerns were noted regarding attrition and variability in intervention delivery. The feasibility RCT by Glover et al. (Gould et al., 2019) and the pilot stepped-wedge RCT by Gould et al. (Glover et al., 2015b) were assessed to be moderate risk, primarily due to small sample sizes, incomplete blinding, and missing outcome data. Patten et al. (Patten et al., 2020) was also rated as moderate risk, due to variable exposure to the peer-counseling intervention.

**Table 2:** Summary of the risk of bias assessment for smoking cessation interventions among Indigenous pregnant women in Australia, New Zealand, and the United States (2012−2020), assessed using an adapted Cochrane Risk of Bias 2 (RoB 2) tool.

Author (year)	Randomization adequately described	Allocation concealment adequate	Participants and personnel blinded	Outcome assessors blinded	Groups similar at baseline	Handling of missing data described	Attrition and withdrawals balanced	Identical treatment of groups	Outcomes measured objectively	Selective reporting avoided	Appropriate statistical analysis	Trial design appropriate	Total score/Overall risk of bias
Eades et al., 2012 (Patten et al., 2019)	Yes	Unclear	No	Yes	Yes	Unclear	Unclear	Yes	Yes	Yes	Yes	Yes	9.50/Low
Patten et al., 2019 (Eades et al., 2012)**,**	Yes	Yes	No	Yes	Yes	Unclear	Unclear	Yes	Yes	Yes	Yes	Yes	10/Low
Glover et al., 2015 (Gould et al., 2019)	Unclear	Unclear	No	Unclear	Yes	No	No	Unclear	Yes	Unclear	Yes	Yes	6.50/Moderate
Gould et al., 2019 (Glover et al., 2015b)	Unclear	Unclear	No	Unclear	Yes	Unclear	Unclear	Yes	Yes	Yes	Unclear	Yes	7.50/Moderate
Patten et al., 2020 (Patten et al., 2020)	Unclear	Unclear	No	Unclear	Yes	Unclear	Unclear	Yes	Yes	Yes	Yes	Yes	8.50/Moderate

Scoring system: Each criterion was rated as Yes (1), Unclear (0.5), or No (0).

Overall risk categories: Low (≥ 9 points/≥ 75%), Moderate (6–8.5 points/50–74%), High (< 6 points/< 50%).

## 4 Discussion

This review examined smoking cessation interventions for pregnant Indigenous women and found that despite the need for effective interventions, only five eligible studies were identified. Across the included studies, biochemically validated quit rates were low and did not differ significantly between intervention and control groups. Nonetheless, we observed an improvement in intermediate outcomes such as quit attempts and quit-date setting. All included studies emphasized the feasibility and cultural acceptability of tailored approaches. These findings underscore both the limited evidence and the considerable challenges of achieving sustained cessation in this population, highlighting the need for innovative and culturally grounded strategies. The inclusion of studies from only Australia and New Zealand highlights a substantial gap in cessation research among First Nations, Inuit, Métis, and other Indigenous populations in Canada and the United States.

We observed modest cessation outcomes in this review, consistent with available evidence. Reviews of psychosocial interventions in the general pregnant population have reported small increases in quit rates compared with usual care, but absolute rates rarely exceed 20% (Chamberlain et al., 2017; Lumley et al., 2009). Pharmacological strategies, including NRT, have shown mixed results in pregnancy, with modest absolute quit rates and uncertainty related to increased nicotine metabolism, and limited safety data for other pharmacotherapies such as bupropion and varenicline (Coleman et al., 2015). In the general pregnancy population, clinical guidelines recommend non-pharmacological interventions, including counseling and behavioral support, as first-line smoking cessation therapy. If these are unsuccessful, pharmacologic options such as NRT, bupropion, or varenicline are considered (Mir et al., 2025). Nicotine metabolism accelerates from mid-pregnancy, often reducing NRT effectiveness; therefore, higher or combination NRT dosing may be needed to control withdrawal and prevent relapse (Mir et al., 2025). Bupropion is usually considered a reasonable alternative, particularly for pregnant individuals with depression (Mir et al., 2025). Although financial incentive interventions have shown the most robust effects in general pregnant populations, nearly doubling smoking abstinence rates (Notley et al., 2019; Berlin et al., 2021), their limited impact in the Māori feasibility trial likely reflects insufficient statistical power and low retention, rather than a true lack of benefit. Likewise, biomarker feedback, which has been shown to increase motivation in other settings (McClure et al., 2009; Hetherington et al., 2012), may require stronger integration into culturally safe models of care to be effective in Indigenous populations.

Although few women achieved abstinence, some interventions influenced readiness to change. Quit attempt and quit-date setting increased in trials incorporating culturally adapted resources and personalized feedback, reflecting early steps in the cessation pathway. Such behavioral shifts indicate engagement and motivation that could translate into quitting if reinforced by sustained support (Chamberlain et al., 2017; Soulakova et al., 2018). However, in Indigenous contexts, structural and social stressors such as systemic racism, poverty, trauma, and stigma may limit the extent to which increased motivation alone can lead to long-term cessation (Gould et al., 2017a; Passey et al., 2013). In addition, early initiation of nicotine use within some Indigenous communities may contribute to higher levels of nicotine dependence, which further complicates cessation efforts in this perinatal population. Addressing these broader determinants through integrated and family- and community-oriented approaches may be useful for behavioral gains to yield durable outcomes.

It is important to note that the limited effectiveness of cessation interventions reflects broader systemic and historical forces rather than individual behavior. Structural determinants including colonial policies, intergenerational trauma, racism, socioeconomic disadvantage, and limited access to culturally safe care shape tobacco use and the ability to quit during pregnancy. As such, individual or culturally adapted strategies alone are insufficient; system-level approaches such as stable funding for Indigenous-controlled health services, community-wide supports, and policies addressing social conditions are needed to improve long-term cessation outcomes.

Across trials, cultural tailoring was central to acceptability. Participants valued interventions that reflected their identities and were delivered in trusted community-controlled health services, while providers found that training and practical tools such as CO monitors facilitated engagement (Eades et al., 2012; Patten et al., 2019; Glover et al., 2015b; Patten et al., 2020). It is worthy to note that intervention delivery within Indigenous health services does not necessarily equate to Indigenous leadership. Across included studies, only a subset of interventions were Indigenous-led or community-controlled. However, given the low recruitment and retention observed in several studies, these findings may not generally reflect the experiences of all participants. These observations align with the importance of cultural safety and community involvement in tobacco control for Indigenous women (Gould et al., 2017a; Wood et al., 2008). These issues are recognized in Indigenous health research, where mistrust of external programs, stigma, and resource constraints often limit participation and sustainability (Glover et al., 2015c). At the same time, feasibility was constrained by recruitment and retention challenges, staff turnover, and inconsistent delivery of key components and limited continuity of care (Patten et al., 2019; Glover et al., 2015b; Patten et al., 2020). Small sample sizes, high attrition, and per-protocol analyses further reduced statistical power to detect clinically meaningful differences. In addition, while cultural tailoring improved acceptability, some interventions may not have fully addressed deeper structural determinants such as chronic stress, racism, poverty, and limited social support. These intersecting factors likely contributed to the low cessation rates across studies. It is also known that pregnancy is associated with unique barriers to research participation, including competing clinical appointments, fatigue, childcare responsibilities, transportation challenges, and shifting priorities across pregnancy, which further compound Indigenous-specific barriers and likely contributed to the high attrition observed in these trials (Shankar et al., 2024; Gao et al., 2024). These findings suggest that effective cessation programs will require both cultural adaptation and structural investment in Indigenous-led health services and workforce capacity. The findings of Healthy Pregnancies Project (Patten et al., 2020) extend earlier evidence by demonstrating that a community-wide, culturally grounded intervention can achieve extensive reach and increase quit attempts, even when abstinence outcomes remain unchanged. This highlights the importance of multi-level, Indigenous-led approaches that integrate behavioral counseling with broader community engagement to sustain tobacco cessation during and after pregnancy.

The available evidence from the included RCTs indicates that culturally adapted interventions alone are not sufficient to achieve sustained abstinence (Eades et al., 2012; Patten et al., 2019; Glover et al., 2015b; Patten et al., 2020). As such, future programs must combine culturally grounded delivery with strategies that have demonstrated effectiveness elsewhere, such as pharmacotherapy, financial incentives, intensive behavioral support, and relapse prevention (Vila-Farinas et al., 2024). Policy makers should prioritize co-designed programs led by Indigenous elders and knowledge keepers, ensure stable funding for Indigenous-controlled health services to enable fidelity, sustainability, and scalability (Washio and Cassey, 2016).

A strength of this review is its focus on a high-priority population that experiences disproportionate tobacco-related harm but has been underrepresented in clinical research. Results of the review provide a broad picture of current knowledge. Limitations include the small number of eligible studies, their modest sample sizes, and heterogeneity in design and outcome measures, which precluded quantitative synthesis, meta-analysis, and subgroup analyses for key demographic and clinical factors. High attrition and incomplete biochemical validation may have biased findings of individual studies, and restricting the review primarily to published trials raises the possibility of publication bias. However, our search strategy, which included Indigenous-specific databases indexing grey and community-based literature, likely reduced this risk and improved the inclusiveness of our search strategy. Nonetheless, Indigenous-led or community-controlled cessation programs may be disseminated outside indexed academic journals, meaning such work could remain underrepresented even with the inclusion of Indigenous-specific databases. The focus on randomized designs may under-represent promising community-based programs evaluated using participatory or qualitative methods. Although vaping was an eligibility criterion, none of the included studies reported vaping-specific outcomes. This reflects a gap in the current evidence base and limits the assessment of vaping among Indigenous pregnant populations.

Future studies should prioritize randomized trials that are Indigenous-led and co-designed with communities. Combining evidence-based cessation strategies with cultural adaptation and community engagement will be critical in designing future intervention trials. Although not evaluated in the included studies, digital health platforms and mobile applications tailored for Indigenous users have shown promise in improving engagement and continuity of cessation support in other populations (Hefler et al., 2019; Loppie et al., 2020). Hybrid models that combine financial incentives, counseling, and family involvement may further strengthen motivation and relapse prevention. There is also the need to extend follow-up beyond pregnancy to address postpartum relapse, for example embedding interventions within routine maternal care pathways. Studies should investigate the predictors of quitting as well as relapse in this population to help improve and tailor smoking cessation interventions.

## 5 Conclusions

Evidence from this review suggests that culturally tailored smoking cessation interventions for pregnant Indigenous women are feasible and generally well accepted, particularly when delivered within Indigenous health or community-controlled services. Interventions such as biomarker feedback counseling, education with pharmacotherapy, and a community-wide multimedia and peer-counseling intervention demonstrated increases in quit attempts, quit-date setting, and engagement, yet sustained cessation remains elusive. The current evidence base consists of a small number of underpowered and heterogeneous trials, limiting inferences about effectiveness and highlighting the need for adequately powered, Indigenous-led, co-designed studies that extend support into the postpartum period.

## Availability of data and materials

Data sharing is not applicable to this article as no datasets were generated or analyzed during the current study.

## CRediT authorship contribution statement

**Dan Yedu Quansah:** Writing – review & editing, Writing – original draft, Methodology, Investigation, Formal analysis, Data curation, Conceptualization. **Salma Mahmoodianfard:** Validation, Methodology, Investigation, Data curation. **Javad Heshmati:** Writing – review & editing, Methodology, Data curation, Conceptualization. **Sarah Visintini:** Writing – review & editing, Methodology, Investigation, Conceptualization. **Meagan Noble:** Writing – review & editing, Validation, Methodology. **Ekua Amponsah Agyemang:** Writing – review & editing, Validation, Methodology, Investigation. **Kerri-Anne Mullen:** Writing – review & editing, Supervision, Methodology, Investigation, Formal analysis, Data curation, Conceptualization. **Hassan Mir:** Writing – review & editing, Visualization, Supervision, Formal analysis, Data curation, Conceptualization.

## Consent for publication

Not applicable.

## Ethics approval and consent to participate

This study is a systematic review of previously published literature and did not involve the collection of primary data from human participants. All data were obtained from publicly available, anonymized sources. In accordance with institutional policies, ethics approval and informed consent were not required.

## Funding

The research of Dr. Dan Quansah and Dr. Javad Heshmati is supported by a Health System Impact Fellowship of the 10.13039/501100000024Canadian Institutes of Health Research. The authors acknowledge the CAN-TAP-TALENT for its role in supporting the completion of this CAN-TAP-TALENT Research Project (awarded to Dr. Salma Mahmoodianfard). The CAN-TAP-TALENT is funded by the Canadian Institutes of Health Research (CIHR)-FRN 184898. The authors acknowledge the Canadian Cancer Society Challenge Grant (Grant # 708258) for its support (awarded to Dr. Hassan Mir). Dr. Hassan Mir is also supported by a Chair in Smoking Cessation from the 10.13039/100008572University of Ottawa Heart Institute.

## Declaration of competing interest

The authors declare that they have no known competing financial interests or personal relationships that could have appeared to influence the work reported in this paper.

## Data Availability

No data was used for the research described in the article.

## Appendix A Supplementary data

Supplementary material 1 References cited within the Review section with their respective citation number.

Supplementary material 2 Detailed search strategy for randomized controlled trials of smoking cessation interventions in Indigenous pregnant women through database searches up to May 2025.
