# Supplementary material for: Smoking cessation intervention for Indigenous pregnant women: A systematic review of randomized controlled trials
Source: Prev Med Rep. 2026 Apr 17;66:103479. doi: 10.1016/j.pmedr.2026.103479 (PMC13126278; doi:10.1016/j.pmedr.2026.103479)
Supplement: Supplementary material 1 — References cited within the Review section with their respective citation number. [file mmc1.docx]

**Supplementary File**

**Five references cited within the Review section with their respective citation number**

**S1**  16. Eades SJ, Sanson-Fisher RW, Wenitong M, Panaretto K, D’Este C, Gilligan C, et al. An intensive smoking intervention for pregnant aboriginal and Torres strait islander women: A randomised controlled trial. Medical Journal of Australia. 2012 Jul 2;197(1):42–6.

**S2** 17. Patten CA, Koller KR, Flanagan CA, Hiratsuka VY, Hughes CA, Wolfe AW, et al. Biomarker feedback intervention for smoking cessation among Alaska Native pregnant women: Randomized pilot study. Patient Educ Couns. 2019 Mar 1;102(3):528–35.

**S3** 18. Gould GS, Bovill M, Pollock L, Bonevski B, Gruppetta M, Atkins L, et al. Feasibility and acceptability of Indigenous Counselling and Nicotine (ICAN) QUIT in Pregnancy multicomponent implementation intervention and study design for Australian Indigenous pregnant women: A pilot cluster randomised step-wedge trial. Addictive Behaviors. 2019 Mar 1;90:176–90.

**S4** 19. Glover M, Kira A, Walker N, Bauld L. Using Incentives to Encourage Smoking Abstinence Among Pregnant Indigenous Women? A Feasibility Study. Matern Child Health J. 2015 Jun 1;19(6):1393–9.

**S5** 20. Patten CA, Lando HA, Desnoyers CA, Bock MJ, Alexie L, Decker PA, et al. Healthy Pregnancies Project: Cluster Randomized Controlled Trial of a Community Intervention to Reduce Tobacco Use among Alaska Native Women. Int J Environ Res Public Health. 2020 Dec 12;17(24):9302.
