# Supplementary material for: Smoking cessation intervention for Indigenous pregnant women: A systematic review of randomized controlled trials
Source: Prev Med Rep. 2026 Apr 17;66:103479. doi: 10.1016/j.pmedr.2026.103479 (PMC13126278; doi:10.1016/j.pmedr.2026.103479)
Supplement: Supplementary material 2 — Detailed search strategy for randomized controlled trials of smoking cessation interventions in Indigenous pregnant women through database searches up to May 2025. [file mmc2.docx]

# Supplemental File

## S1. Medline Search

| Database: MEDLINE(R) ALL 1946 to May 27, 2025  Platform: Ovid  Date Searched: May 28, 2025 | | |
| --- | --- | --- |
| **#** | **Searches** | **Results** |
| 1 | exp Indigenous Peoples/ | 31466 |
| 2 | Ethnopharmacology/ | 2152 |
| 3 | Health Services, Indigenous/ | 4579 |
| 4 | exp Medicine, Traditional/ not (Chinese or China).ti,ab,kf. | 28919 |
| 5 | Shamanism/ | 245 |
| 6 | ((traditional adj1 (medicine* or heal* or food* or health*)) not (Chinese or China)).ti,ab,kf. | 24123 |
| 7 | (Aboriginal? or aborigine? or aborigen* or amerindian* or amerindio* or indian? or indigene* or indigenous* or indigena* or nativo or originarios or tribe? or tribal*).ti,ab,kf. | 181234 |
| 8 | or/1-7 [Indigenous, Broad] | 237794 |
| 9 | Arctic Regions/ or Nunavut/ | 8296 |
| 10 | (Aamjiwnaang or Abenaki? or Abitibiwinni or Ahtahkakoop or Aishihik or Aklavik or Akuliarmiut* or Akwesasne* or Alert Bay or Aleut* or Alexandra Fiord or Algonqui?n* or Amadjuak or Amerind* or Amisk or Anish?na?b?e* or Aquiatulavik Point or Arctic or Armstrong settlement or Aroland or Arviat or Asimakaniseekan or Assabaska or Assiniboine or Athabasca? or Athapaskan or Atikamek* or Attawapiskat or Aundeck-Omni-Kaning or autochtone*).ti,ab,kf. | 20411 |
| 11 | (Baffin Island or Baker Lake or Barriere Lake or Batchewana or Bathurst Inlet or Beardy or Bearfoot Onondaga or Beaver Creek or Behochoko or Belcher Islands or Beothuk* or Berens River Bloodvein or Betsiamite* or Bibigo?ining or Biinjitiwabik Zaaging or Bimose or Bingwi Neyaashi or Birch Portage or Bittern Lake or Bkejwanon* or Blackfoot or Bois-brule* or Brochet or Budd's Point or Buffalo River or Bungee or Bunibonibee or Bylot Island).ti,ab,kf. | 624 |
| 12 | (Cacouna or Cambridge Bay or Canoe Lake or Canupawakpa or (Cape adj1 (Dorset or Dyer or Smith)) or Carcross or Carmacks or Carrot River or Carry the Kettle or Cayuga or Charlton Depot or Chesterfield Inlet or Chipewyan or Chipp#wa* or Chisasibi or Clyde River or Coal Harbour or Colville Lake or Coral Harbour or Coucoucache or country food* or Craig Harbour or Cree or Cypress Health).ti,ab,kf. | 1429 |
| 13 | (Dakelh or Dakota or Dauphin River or Dawson City or Day Star or Deh Cho or Deline or Dene or Denesuline or Destruction Bay or Devon Island or Dipper Rapids or Dogrib* or dokis or Dopitciwa* or Dundas Harbour or Dunne?za).ti,ab,kf. | 3739 |
| 14 | (Eabametoong or (Eagle adj (Plains or village)) or Eastmain or Eeyou Istchee or Ehdiitat or Ekuanitshit* or Elak Dase or Ellesmere Island or Ennadai or eskimo* or Esquimau* or Essipit* or ethnomedicin* or ethnopharmacol*).ti,ab,kf. | 18407 |
| 15 | ((first adj2 (nation? or people?)) or First-Nation? or Firstnation? or Fisher River or Fond du Lac or (Fort adj1 (Conger or Good Hope or Hope or Liard or McPherson or Providence or Ross or Selkirk or Simpson or Smith)) or Four Portages).ti,ab,kf. | 5608 |
| 16 | (Grandmother's Bay or Gespeg or Gesgapegiag* or Ginoogaming or Gits#an or Gjoa Haven or (God's adj1 (river or lake)) or Grise Fiord or Gwi?ch?in or Gwichya).ti,ab,kf. | 25 |
| 17 | (Haida or Haines Junction or Haisla or Hall Beach or (Hare adj1 (boy* or girl* or men or man or woman or women or people* or person or persons or band? or native* or tribe or tribal)) or Hatchet Lake or Haudenosaunee or Hay River or Hazen Camp or Heiltsuk or Huron or Huron-Wendat or Huronne-Wendat).ti,ab,kf. | 544 |
| 18 | (Han not (Chinese or China)).ti,ab,kf. | 8863 |
| 19 | (Igloolik or Igluligaarjuk or Ikaluit or Ils a la Crosse or ((indian or council) adj3 band) or Innu? or Inuit* or Inuk* or Inupiat* or Inuvik or Inuvialu* or Inuinnaqtun or Iqaluit or Iqaluktuttiaq or Iroquois or Isachsen or Island Lake or Itivimiut* or Ivujivik).ti,ab,kf. | 2997 |
| 20 | (James Bay or Joseph Bighead).ti,ab,kf. | 157 |
| 21 | (Kabapikotawangag or Kalaallit* or Kahkewistahaw or Kahnawa?ke or Kanesatake or Kasabonika Lake or Kashechewan or Kaska? or Kawacatoose or Kawawachikamach* or Kebaowek or Keeseekoowenin or Kekerten or Keno City or Keewaytinook or Kee?Way?Win or Kelsey Trail Health or Kiashke Zaaging or Kimmirut or Kingnaitmiut* or King William Island or Kinistin or Kinonjeoshtegon or Kipawa or Kipisa or Kitchenuhmaykoosib or Kitcisakik or Kitigan Zibi or Kitikmeot or Kitimat or Kitsakie or Kivalliq or Kivitoo or Konadaha Seneca or Koocheching or Ktunaxa or Kugaaruk or Kugluktuk or Kutchin* or Kuujjuaq or Kwakiutl or Kwakwaka?wakw).ti,ab,kf. | 162 |
| 22 | (La Plonge or (Lac adj (Brochet or Romanie or John or Simon or La Hache or La Ronge)) or Lake Manitoba or Lean Man or Lenape or (Little adj (Black Bear or Saskatchewan or Red River or Salmon)) or Listuguj* or Long Point First or Lucky Man).ti,ab,kf. | 55 |
| 23 | (M?Chigeeng or MacDowell Lake or Mackenzie River Basin or MacKenzie Valley or Magnetawan or Maguse River or Makaw or Makaoo or Malecite* or Maliotenam or Maliseet or Mamawetan or Mamit Innuat or Mamuitun or Manawan or Mani-Utenam or Manitoulin or Manto Sipi or Mashteuiatsh or Matachewan or Mathias Colomb or Matimekosh* or Matimekush* or Mattagami or Mawiomi or (medicine adj (man or men or woman or women)) or Metchif or Metif or metis or Miawpukek or Michif or Mic?mac or mic mac or Migmaw or Mig maw or Mi?gmaq or Mi?gmawei or Mi?kmaq or Mi#chif or Mingan or Ministikwan or Minoahchak or Mirond Lake or Mishkeegogamang or Missanabie or Mistawasis or Mistissini* or Mitaanjigaming or Mixed-blood* or Mo?hawk or Mocreebec or Montagnais or Moos-Toosis or (moravian adj2 thames) or Moraviantown or Morin Lake or Mosquito-Grizzley Bear* or Muscowpetung or Mushkegowuk or Muskoday or Muskowekwan).ti,ab,kf. | 1749 |
| 24 | ("Nacho Nyak Dun" or Naicatchewenin or Nain or Nako#a or Namaygoosisagagun or Nan#sivik or Naongashiing or Naotkamegwanning or Naskapi* or Natashquan* or (native adj2 (group? or health or community or communities or person$ or people$ or population* or america* or canad* or Nation or band or bands or reserv*)) or (Native* adj1 (man or men or women or woman or boy* or girl* or child* or adolescent* or youth? or adult*)) or Nekaneet or Nelson House or Nemaska* or Nemiscau or Neskantaga or Netsilik or New Thunderchild or Nibinamik or Nigigoonsiminikaaning or Nihtat or Nipissing or Nisga?a or Nisichawayasihk or Nlaka?pamux or Norman Wells or Northlands Nursing Station or North Slave or (northern adj1 (Saskatchewan or Manitoba or Quebec or Alberta or British Columbia or Ontario)) or Norway House or Nottingham Island or Nugumiut* or Nunatsiavut or Nunav* or Nutaqqavut or Nuwata or Nuxalk or Nuu-chah-nulth).ti,ab,kf. | 21320 |
| 25 | (ob?djiwan or Obashkaandagaang or Ocean Man or Ochapowace or O-Chi-Chak-Ko-Sipi or Ochiichagwe or Odanak or off-reserve or Oji-Cree or Ojibw* or Okanagan or Okanese or Okemasis or Oki or Old Crow or One Arrow or Oneida or Onion Lake or Onigaming or Onkwehonwe or Onodaga or on-reserve or Opaskwayak or Opawakoscikan or O-Pipon-Na-Piwin or Opitciwa* or Oqomiut* or Original people? or Ouj?-Bougoumou or Ouje?Bougoumou or Oweenkeno).ti,ab,kf. | 1076 |
| 26 | (Padlei or Padloping Island or Pakua Ship* or Pakuaship* or Pangnirtung or Parmachene or pasqua or Passamaquoddy or Pauingassi or Pauktuutit or Paulatuk or Peepeekisis or Peguis or Pelly or Perry Island or Pessamit* or Peter Ballantyne or (Peuple adj2 (premier or racine or natif*)) or Pheasant Rump or Piapot or Pikogan or Pikwakanagan or Pinaymootang or Pine Bluff or Pond Inlet or Port Burwell or Potato River or Potawatomi or Prairie North Health or premiere nation or Prince Albert Parkland Health or Pukatawagan).ti,ab,kf. | 85 |
| 27 | (Qaumauangmiut* or Qayuqtuvik or Qikiqtarjuaq or Qikqta* or Qingaumiut* or Qoloqtaaluk).ti,ab,kf. | 10 |
| 28 | (Rankin Inlet or Rapid Lake or Read Island or Red Pheasant or red road or Red Sucker Lake or Repulse Bay or residential school* or Resolute Bay or Resolution Island or Roseau River).ti,ab,kf. | 507 |
| 29 | (Sabaskong or Sachs Harbour or Sagamok or Sagkeeng or Sahtu or Sakimay or Salish or Sa?lteaux or Sandy Narrows or Sanikiluak or Sanikiluarmiut* or Sapotaweyak or Saulteaux or Saumingmiut* or Sayisi Cree or Schefferville or Sec wepmc or Seekaskootch or Sekani or Sekon or Seneca or Sept-Iles or shaman* or Shamattawa or Shawanaga or Sheguiandah or Shesheep or Sheshegwaning or Shoal Lake or Shubenacadie or Sikusilaamiut* or Sioux or Siphik or Six Nations or Skownan or Slave Lake or Slavey* or Southend or South Indian Lake or South Slave or Sunrise Health or Standing Buffalo or Stanjikoming or Stanley or Starblanket or Stl?atlimc or St Theresa Point or Sturgeon or Subarctic or Sucker River or Sweetgrass).ti,ab,kf. | 7201 |
| 30 | (Tadoule Lake or Tagish or Tahltan or Takuaikan or Talirpingmiut* or Talo?yoak or Ta?an Kwach?an or Tanana? or Tanquary Camp or Tarramiut* or Tasttine or Tataskweyak or Tavani or Taykwa Tagamou or Temagami or T#mis#aming or Teslin or Tetlitn or Thom Bay or Tlingit or Tlicho or Tli Cho or Tootinaowaziibeeng or Tr?ondek Hwech?in or treaty or treaties or Trout Lake or Tsilgehtchic or Tsilhqot?in or Tsimshian or Tsuu T?inia or Tulita or Tuktoyaktuk or Tumor Lake or Tungasugit or Turtle island or Tuscarora or Tutchone* or Tuttarvingat).ti,ab,kf. | 2892 |
| 31 | (Uashat or Ulukhaktck or Umingmaktok or Unamen Shipu or Ungava or Upper Liard or Uqqurmiut* or (urban adj3 (Indian* or Native* or Aboriginal* or indigenous*))).ti,ab,kf. | 2101 |
| 32 | (Victoria Island or Vuntut).ti,ab,kf. | 48 |
| 33 | (Waban-Aki or Wabaseemoog or Wabauskang or Wabigoon Lake or Wager Bay or Wahgoshig or Wahnapitae or Wahpeton or Wakashan or Wapachewanak or Wapekeka or Wa-Pii or Wasagamack or Wasauksing or Washagamis Bay or Waskaganish or Waswanipi or Waterhen or Watson Lake or Wauzhushk or Wawakepewin or Waywayseecappo or Webequie or Weenusk or Wemindji or Wemotaci or Wendake or Wendat* or Wet?suwet?en or Whale Cove or Whapmagoostui or (White adj (Bear or Cap)) or Whitehorse or Wikwemikong or Willow Bunch or Winneway or Witchekan or Wolastoqiyik or Wolinak or Wood Mountain or Woody Lake or Wrigley or Wuskwi or Wyandot).ti,ab,kf. | 250 |
| 34 | (Yellowkni* or Yellowquill or Yellow Quill or York Landing or Yukon).ti,ab,kf. | 933 |
| 35 | or/9-34 [Indigenous, Canada] | 94652 |
| 36 | Alaska/ | 6504 |
| 37 | ("A' ani" or Absaroka or Haaninin or Atsina or "Gros Ventre" or Acopsel or Tlacopsel or Lacopsel or Ahtna or Ahtena or Akenitsi or Occaneechi or Akokisa or Horcoquisa or Orcoquizas or Aleut or Unangax or Unangan or Alibamu or "Alabama Alsea" or Alutiiq or Sugpiag or Amahami or Awaxawi or Androscoggin or Arosaguntacook or Ameriscoggin or Anishinaabeg or Chippewa or Anihsinape or Saulteaux or Apalachee or Aranama or "Texan Coahuilteca" or Tamique or Arikara or Sahnish or Arickaree or Adakadaho or Assiniboine or Nakota or Nakoda or Nakona or "Atsa' Kudok-wa" or Awatixa or Bannock or "Snake Indian*" or Bidai or Quasmigdo or Biloxi or Blackfoot or Niitsitapi or Sikasikaitsitapi or Cahto or Kaipomo or Cahuilla or Ivilyuqaletem or Ivilyuat or Catawba or Inna or Iswa or Chemehuevi or Chickasaw or "Chilula Chimakum" or Aqokulo or Chimariko or Chiricahua or Tsokanende or Chitimacha or Chetimachan or Sitimacha or Chowanoke or Roanoke or Chumash or Ciboney or "Taino Ciwat" or Clatsop or Coos or Coosa or Uchis or Chiaha or Coste or Talisi or Coquille or Kokwell or Coso or Cowlitz or Taitnapam or "Crow Nation" or "Cui Ui Ticutta" or Cupeno or Kuupangaxwichem or Cupa or "Cup' ig" or Nunivak or "Dakota Oyate" or Lakota or Nakota or Santee or Teton or Sioux or Deadose or "Deg Xina" or "Deg Xit' an" or Kaiyuhkhotana or "Deg Hit' an" or "Dena' ina" or Tanaina or "Dichinanek' Hwt' ana" or "Upper Kuskokwim Athabascan*" or Kolchan or Goltsan or "Tundra Kolosh" or "Do lkabya" or Duwamish or Esselen or Eyak or "Gidi' tikadi" or Guwevkabaya or "Gwich' in" or Kutchin or Haida or Xaadas or Xaat or Halchidhoma or Havasupai or "Green Water People" or Hiratsa or Hiraaca or "Ho-chaaqa" or Winnebago or Holikachuk or Innoko or "Tlegon-khotana" or Hopi or "Houma-Louisiana" or Huaco or Waco or Hualapai or Hupa or Natinixwe or "Natinook-wa" or "Hwech' in" or Hankutchin or "Iroquois Confederacy" or "Hodinoso ni" or "Illinois Confedera*" or Ilinoweg or Illini or Inupiat or Inuit or Ioway or Baxoje or Jicarilla or Juaneno or Acjachemen or Jumano or Kalapuya or Clackama or Kalispel or "Pend d' Oreilles" or Qlispe or Karuk or Karok or "Chum-ne" or Katkoc or Kansa or Kanza or Kawaiisu or Nuwa or Kennebec or "Kinipekw Kittitas" or Klickitat or "Qwu' lh-hwai-pum" or "Awi-adshi" or Mahane or Wahnookt or "Koa' aga' itoka" or Keresan or Kichai or Kitsai or Keechi or "K' itaish" or Kiowa or Gaigwu or Cauigu or Kutjau or "Kwu-da" or "Tep-da" or Kitanemuk or Kittitas or Klickitat or "Qwu' lh-hwai-pum" or "Awi-adshi" or Mahane or Wahnookt or "Koa' aga' itoka" or Konkow or "Koop Ticutta" or Koyukon or Ktunaxa or Kootenai or Kucadikadi or "Kotsa' va" or Kumeyaay or "Tipai-Ipai" or Kamia or Diegueno or Kwapa or Cocopah or Cucapa or "Xawitt kwnchawaay" or Lassik or Lenape or "Leni-Lenape" or Lipan or Luiseno or Payomkawichum or Madqwadabaya or "Desert Yavapai" or Mahican or Mohicans or Makah or Makuhadokado or Maliseet or Wolistoqiag or Manahoac or Mahock or Meipontsky or Mandan or Mattole or "Bear River" or "Tul' bush" or "Ni' ekeni" or Meherrin or Menominee or Mackinac or Mescalero or Myaamiaki or Kickapoo or Twigtwee or Missouria or Miwok or Miwuk or Moadokado or Modoc or Mohave or "Aha Makhav" or Mohawk or "Kaneng' hega" or Molala or Molale or Molele or Nyyhmy or Moosonee or "Moose Cree" or Monsonis or Multnomah or (Chinook not cultivar) or Nabedache or Nabaydacu or Wawadishe or Nabiltse or Dakubetede or "Nacho Nyak Dun" or Tutchone or Nacono or "Na' isha" or Nanticoke or Navajo or Ndee or Nial or Niimiipu or "Nez Perce" or Watapala or Watapahlute or Nisenan or Nisqually or Nomlaki or Noamlakee or "Central Wintun" or Nongatl or Nottoway or Cheroenhaka or "Northern Cheyenne" or Ohlone or Costanoan or Omaha or "O' odham" or Pima or Papago or Osage or Otoe or Otse or "Ozav Dika" or Palus or Passamaquoddy or Pestomuhkati or Patiri or Petaros or Pastia or Patwin or "Southern Wintun" or Panis or Skidi or Pedee or Penobscot or "Petun Piipaash" or "Kokmalik' op" or Piscatawa or Doeg or Conoy or "Pit River" or Pomo or Kashaya or Ponca or Ponka or Pottawatomi or Bodewadmik or Powhatan or Puyallup or Spuyalepabs or Quapaw or Ugahxpa or Quechan or Kwtsaan or Quileute or Salinan or Saponi or Monacan or Sapon or "Eastern Blackfoot" or Christanna or Sawawatodo or Serrano or Taaqtam or "Maarenga' yam" or Yuhaviatam or Shasta or Chasta or Sasti or Shoshone or Siletz or Sinkine or Sinkyone or "Siuslaw Umpqua" or Skitswish or "Schitsu' umash" or Snohomish or Snuqualmi or Sokoki or Missiquoi or Stillaguamish or Stoluckwamish or Suquamish or Sutaio or Swinomish or Skagit or Syilx or Sotaae or "Taga Ticutta" or Takelma or Dagelma or Taltushtuntede or Galice or "Tanan Gwich' in" or Taos or Taovaya or Tataviam or Alliklik or Tawakoni or Tahuacano or Tenino or Thawikila or Hathawekela or "Fort Ancient" or Tigua or Tillamook or Nehalem or Timbisha or Panamint or Timpanogos or Tlingit or "Toi Ticutta" or Tolowa or "Talawa Dini' " or Tongva or Gabrieleno or Fernandeno or Tobikhar or Tonkawa or Ticanwatic or Tsikip or Appalousa or Opelousa or Tsitsistas or Tubatulabal or Tukabatchee or Tuscarora or Tomahittan or Kuskarawock or Tutelo or Tutero or Totteroy or Tutera or Yusan or Tututni or Umatilla or Umpqua or Waccamaw or Waxmaw or Wadatika or "Harney Valley Paiute" or Wailiki or Waluulapam or "Walla Walla" or Walpapi or Huipui or Wampanoag or Massasoit or Wanapum or Wappo or Washoe or Wichita or Willapa or Kwalhioqua or "Wi pukba" or "Verde Valley Yavapai" or Wintu or "Northern Wintun" or Wiyot or "Wee' at" or Weyet or Yakama or "Yamosopo Tuviwarai" or Yaqui or Yoeme or Yatasi or Yattasih or "Yavbe' " or "Yavapai" or "Ysleta del Sur" or Yojuane or Yokuts or Mariposa or Yuki or Yupighyt or "Yup'ik" or Yupik or Yurok or "Olekwo'l" or Zuni or "Native Hawaiian*" or Kanaka or Maoli or Oiwi).ti,ab,kf. | 16233 |
| 38 | ((Applegate or Alabama or Delaware or Flathead or Hohe or Iowa or Ishak or Kaw or Kato or Spokane or Miami or Arkansas or Tali or Tunica or Pawnee or Okanagan or "Coeur D' Alene" or Piscataway or Ree or Tula or Wichita or Yuma) adj3 (reservation* or nation or people or peoples or population or man or men or woman or women or child* or youth* or elder or elders or communit* or tribe or tribes or tribal or Indian*)).ti,ab,kf. | 2366 |
| 39 | or/36-38 [Indigenous, United States] | 24675 |
| 40 | ("Abakuria" or "Abaluhya" or "Abagusii" or "Abakuria" or "Aembu" or "Agikuyu" or "Akamba" or "Anuak" or "Anywaa" or "Amazigh" or "Ambala" or "Ambeere" or "Ambundu" or "Ambuun" or "Amharan" or "Angba" or "Baaka" or "Baamba" or "Babindi" or "Babini" or "Baboma" or "Bachokwe" or "Bacwa" or "Bafumbira" or "Baganda" or "Bagyele" or "Bagwere" or "Bagyeli" or "Bakiga" or "Bakola" or "Baholo" or "Bakalanga" or "Bakiga" or "Bakolo" or "Bakongo" or "Bakonjo" or "Baluba" or "Balunda" or "Balovale" or "Bamasaba" or "Bambuti" or "Bangala" or "Bangoli" or "Bangungu" or "Bantu" or "Banyankole" or "Banyarwanda" or "Banyole" or "Banyoro" or "Bapende" or "Bapedi" or "Barabaig" or "Barombi" or "Barundi" or "Baruuli" or "Basamia" or "Basoga" or "Batswana" or "Batooro" or "Batsamba" or "Batswana" or "Batwa" or "Bayaka" or "Bedzan" or "Bazombe" or "Bebayaka" or "Bedzan" or "Bhaca" or "Biaka" or "Borana" or "Chewa" or "Copts" or "Cormorian" or "Cushitic" or "Dahalo" or "Datooga" or "Dikidiki" or "Dogon" or "Ewondo" or "Fulani" or "Fuliru" or "Ganguela" or "Gciriku" or "Gyele" or "Hadza" or "Hadzabe" or "Haillom" or "Haratin" or "Herero" or "Himba" or "Hlubi" or "Iriryen" or "Iqvayliyen" or "Kabyle*" or "Kalenjin" or "Kanioka" or "Kanioka" or "Kaonde" or "Karamojong" or "Kavango" or "Kereuyu" or "Khoikhoi" or "KhoiSan" or "Kikuyu Kwangali" or "Lokele" or "Lowme" or "Lotuko" or "Lwalwa" or "Maasai" or "Makonde" or "Makua" or "Mande" or "Masalit" or "Matumbi" or "Mayeuyi" or "Mayeyi" or "Mbenga" or "Mbukushu" or "Mbochi" or "Mboro" or "Mbuti" or "Medzan" or "Mijikenda" or "Mozabite*" or "Nafusa" or "Ndebele" or "Ngombe" or "Namaqua" or "Nyanga" or "Nyamwezi" or "Ogiek" or "Ovambo" or "Ovimbundu" or "Phuthi" or "Pokomo" or "Rendille" or "Riffian" or "Riffians" or "Sakuma" or "Samburu" or "Sandawe" or "Sangha" or "Sango" or "Sengwer" or "Serer" or "Sesotho" or "Shangaan" or "Shawiya" or "Shenwa" or "Shi" or "Shilluk" or "Sukua" or "Sukus" or "Swahili" or "Tabwa" or "Tambuka" or "Taveta" or "Thembu" or "Tigrayan" or "Topoke" or "Tsonga" or "Toubou" or "Tuareg" or "Tumbuka" or "Ugana" or "Wochua" or "Xhosa" or "Xindonga" or "Yoruba" or "Zenati" or "Zuwara" or (("Indigenous" or "Afar" or "Afars" or "Aka" or "Akie" or "Ameru" or "Asua" or "Ateker" or "Atwot" or "Awjila" or "Bafia" or "Baka" or "Bakongo" or "Bakwe" or "Balunda" or "Balovale" or "Bango" or "Bassa" or "Beja" or "Bekpak" or "Bemba" or "Bembe" or "Benet" or "Berber" or "Berbers" or "Bira" or "Bowe" or "Bubi" or "Budja" or "Bulu" or "Bunrun" or "Chaga" or "Chopi" or "Damara" or "Dinka" or "Djerba" or "Duala" or "Dzing" or "Efe" or "Elmolo" or "Fang" or "Foora" or "Fula" or "Fur" or "Ghomara" or "Ghadames" or "Gllana" or "Glu" or "Gogo" or "Gongo" or "Haya" or "Havu" or "Hema" or "Hima" or "Hunde" or "Hutu" or "Huva" or "Iboko" or "Igbo" or "Ijo" or "Jieng" or "Kadu" or "Kande" or "Kango" or "Katla" or "Kgaga" or "Khoe" or "Kola" or "Komo" or "Kota" or "Kua" or "Kuba" or "Kwango" or "Kx'z" or "Kxoe" or "Lala" or "Lozi" or "Luo" or "Luba" or "Lupu" or "Masmuda" or "Matmata" or "Mbala" or "Mbam" or "Mbo" or "Mbolo" or "Mbuza" or "Mongo" or "Mpondo" or "Myene" or "Naadh" or "Nama" or "Nande" or "Naro" or "Ngoni" or "Ndau" or "Ndebele" or "Ngoli" or "Ngondi" or "Ngoni" or "Nguni" or "Nkoya" or "Nkumu" or "Nuba" or "Nubian" or "Nuer" or "Nzebi" or "Ogoni" or "Omoro" or "Oroko" or "Pygmy" or "Popoi" or "Poto" or "Puru" or "Rashad" or (San not ("San Francisco" or "San Diego" or "San Antonio")) or "Sango" or "Sanhaja" or "Sena" or "Shilha" or "Shira" or "Shona" or "Shua" or "Sokna" or "Somali*" or "Sotho" or "Sua" or "Subu" or "Swazi" or "Taitaa" or "Tchokwe" or "Teke" or "Tembo" or "Tetela" or (Tonga and Africa*) or "Tshwa" or "Tsoa" or "Twa" or "Turkana" or "Tuu" or "Venda" or "Vira" or "Watta" or "Wakuti" or "Yaaku" or "Yaka" or "Yakoma" or "Yanzi" or "Yao" or "Yeke" or "Yela" or "Yeyi" or "Zulu") adj3 (population* or people or peoples or person or persons or elder or elders or man or men or woman or women or child* or youth* or clan or clans or tribe or tribes or tribal or family or families or parent* or grandparent* or elder or elders or grandmother* or grandfather* or baby or babies or infant or infants or patient or patients or speakers or speaking or village* or communit*))).ti,ab,kf. [Indigenous, Africa] | 33801 |
| 41 | "torres strait islander*".ti,ab,kf. [Indigenous, Australia] | 3105 |
| 42 | (Acatec or Aguacateco or Amuzgo or Bokota or Boruca or Bribri or "Bri Bri" or Buglere or Cabecar or Cakchiquel or Changuena or Chatino or Chiapanec or Chicomuceltec or Chinantee or Chocho or Cholti or "Ch'olti'" or "Ch'olti'anor Chontal" or Chorotega or Chorti or Chuj or Chumbia or Corobici or (Cueva not Spain) or Cuicatec or Cuitlatee or Cuytec or Dorasque or Embera or Garifuna or Guatuso or Guaymi or Guaymis or Guetar or Huastec or Huave or Huetar or Itzaj or Ixil or Jacalteco or Jonaz or Kanjobal or Kekchi or Kuna or Maleku or Mangue or Matambu or Matlatzinca or Mazahua or Motozintlec or Mayan or Mayangna or Mestizo or Miskito or Mixtec or Mopan or Nahua or Nahuatl or Ngabe or Otomi or Pantec or Paya or Popoloca or Popoloc or Poqomam or Poqomchi or "Q'eqchi'" or Quiche or Quitirrisi or Sacapulteco or Sipacapense or Subtiaba or Tacaneco or Tarasco or Tamaulipec or Tamazultec or Tecoxquin or Tectiteco or Tecual or Tecuexe or Tepehura or Tepuztecor or Teribe or Terraba or Totonac or Trique or Tzeltal or Tzotzil or Tzutujil or Ulwa or Uspantec* or Uspanteko or Voto or Xinca or Waunana or Wounaan or Yucatec or Zapotec or Zoque).ti,ab,kf. [Indigenous, Central America] | 2845 |
| 43 | exp China/ and minority group/ | 589 |
| 44 | (((China or Chinese) and "ethnic minorit*") or "Achang" or "Bonan" or "Bouyei" or "Blang" or "Deang" or "Dongxiang" or "Dulong" or "Ewenki" or "Gaoshan" or "Gelao" or "Hezhe" or "Jingpo" or "Jinuo" or "Kazak" or "Kirgiz" or "Korean" or "Lahu" or "Luoba" or "Manchu" or "Maonan" or "Menba" or "Miao" or "Mongolian" or "Mulao" or "Naxi" or "Oroqen" or "Ozbek" or " Pumi" or "Qiang" or "Shui" or "Tajik" or "Tatar" or "Tibetan" or "Tujia" or "Uigur" or "Yugur" or "Zhuang" or (("Hui" or "Yi" or "Dong" or "Yao" or "Bai" or "Hani" or "Li" or "Dai" or "She" or "Lisu" or "Va" or "Sui" or "Tu" or "Xibe" or "Daur" or "Salar" or "Nu" or "Gin" or "Jino") adj3 (ethnic or minorit* or population* or people or peoples or person or persons or elder or elders or man or men or woman or women or child* or youth* or clan or clans or tribe or tribes or tribal or family or families or parent* or grandparent* or elder or elders or grandmother* or grandfather* or baby or babies or infant or infants or patient or patients or speakers or speaking or village* or communit*))).ti,ab,kf. | 105402 |
| 45 | or/43-44 [Indigenous, China] | 105604 |
| 46 | Greenland/ | 2791 |
| 47 | (Greenland* or Kalaallit or Kalaallisut or Nuuk or Sisimiut or Ilulissat or Qaqortoq Aasiaat or Maniitsoq or Tasiilaq or Uummannaq or Narsaq or Paamiut or Nanortalik or Upernavik or Qasigiannguit or Tunumiit or Inughuit or Avanersuarmiut).ti,ab,kf. | 4875 |
| 48 | or/46-47 [Greenland] | 5184 |
| 49 | ("Adivasis" or "Adnamanese" or "Andaman" or "Baluch" or "Baluchis" or "Bodo" or "Boro" or "Boros" or "Bote" or "Brahuis" or "Chakmas" or "Chepang" or "Chhantyal" or "Damai" or "Dewan" or "Ghale" or "Gurkha" or "Gurung" or "Hayu" or "Hyolmo" or "Jarawa" or "Jirel" or "Jumma" or "Kalash" or "Khas" or "Kirati" or "Koinch" or "Kulung" or "Kusunda" or "Limbu" or "Lohorung" or "Magar" or "Makrani" or "Mangar" or "Marma" or "Miji" or "Mongar" or "Mro" or "Naga" or "Nepami" or "Newar" or "Nicobar" or "Onge" or "Rai" or "Rang" or "Raute" or "Sajolang" or "Santhal" or "Sentinelese" or "Sindhis" or "Sulemani" or "Sunuwar" or "Tamang" or "Thakali" or "Thangmi" or "Tharu" or "Tripura" or "Tumbahangphe" or "Wanniyala-Aetto" or "Yakkha" or "Yolmopa").ti,ab,kf. [Indigenous, Indian Subcontinent] | 8993 |
| 50 | (Aguacatec or Akwa'ala or Abxubal or Ayuukja'ay or "Batzil k'op" or Binizaa or "Chichimeca Jonaz" or Chinantec or Chocho or "Ch ol" or Chontal or "Chuj" or Cochimi or Comcaac or Hamasipini or Harijio or "Ha shuta enima" or "Hach t'an" or Huastecor Hnahnu or Hnatho or "indos mexicano" or Ixcatec or Ixil or Jacaltec or K'akchikel or K'anjobal or Kanjobal or Kaqchikel or Kechi or K'iche or Kikapooa or Kikapu or Kiliwa or "Ko'lew" or "K'op o winik atel" or Kumiai or Lacandon or Laymon or Makurawe or Maya or Maya'wiinik or Mazahua or Mazatec or Me'phaa or Mesoamerindian* or Mexicanero or Mexikatlajtolli or "Mixe" or Mixtec or Motocintleco or Mti'pa or Nahuas or Ocuiltec or Otomi or (Oaxaca not Oaxaca-Blinder) or "Pame" or Papago or Tlahuica or Paipai or "Pima Bajo" or "pueblos indigenas" or Purepecha or P'urhepecha or Qatok or (Quiche not Guatemala) or Q'iche or Raramuri or "Runixa ngiigua" or ("Seri" not "Seri 82") or "Slijuala sihanuk" or Tacuate or Tarahumara or Teenek or Tepehua or Ti'pai or Tlapanec or "Tohono O'odham" or Totonac or Tachiwin or "Tsa jujmi" or Tzotzil or "Tu'un savi" or Tzeltal or "Uza" or "Winik" or Xigue or Yucatec or Zapotec).ti,ab,kf. [Indigenous, Mexico] | 3336 |
| 51 | ("Bedouin*" or "Jahalin" or "al-Kaabneh" or "al-Azazmeh" or "al-Ramadin" or "al-Rshaida").ti,ab,kf. | 1260 |
| 52 | ((nomadic or seminomad* or "semi nomad*") adj3 ("people" or "peoples" or "elder" or "elders" or "grandmother*" or "grandfather*" or "parent*" or "women" or "men" or "woman" or "man" or "child*" or "youth" or "youths" or "baby" or "babies" or "tribe" or "tribes" or "tribal" or "shaman*" or "native" or "patient*")).ti,ab,kf. | 270 |
| 53 | or/51-52 [Indigenous, Middle East] | 1519 |
| 54 | (maori* or maaori* or "m aori" or "ma ori" or moriori or tangata or whanau or rangatahi or tamariki or wahine or marae or hauora or kaupapa or tinana or hinengaro or wairua).ti,ab,kf. [Indigenous, New Zeland] | 5218 |
| 55 | (Saami or Sampi or (Sami not Ulus) or Samis or Southernsami* or Umesami* or Pitesami* or Lulesami* or Northernsami* or Enaresami* or Kolasami* or Lapp or Lapps or Lappish or Lappland or (Lapland* not longspur) or Lappalainen* or Saamelainen* or reindeer herd* or reindeer culture* or reindeer pastoral* or Lappbys or Samebys or reinbeitesdistrikt or paliskunta or siida).ti,ab,kf. [Indigenous, Northern Europe] | 1369 |
| 56 | (Ainus or Ainu or Aleuts or Alyutors or Chukchis or Chuvans or Dolgans or Enets or Entsy or Yupik or Yup'ik or Yuit or Yupigyt or Chaplino or Naukan or Itelmens or Kamchadals or Kereks or "Komi" or Koryaks or Nenets or Nentsy or Nganasans or Tavgi or Sami or Veps or Yukaghirs or Chulyms or Evenks or Tungus or Evens or "Kets" or Khantys or Mansi or Vguls or Selkups or Teleuts or Nanais or Nanaitsy or Negidal or Nivikh or Oroch or orok or Taz or udege or ulch or Kumadins or Chelkans or Shorians or Soyots or Telengits or Tofalars or Tugalars or "Tufans" or "Todzhins" or Laks or Tabasarans or Turuls or Aguls or Tsakhurs or Kumyks or Nogais or "Andis" or Akhvakh or Archins or Bagvalals or Bezhta or Botlikhs or Chamalals or Godoberi or Hinukh or Hunzibs or Khwarshi or Karata or Tindis or Tsez or Abazin or Besermyan or Izhorians or Karelians or Nagaybaks or Setos or Shapsugs or Quratay).ti,ab,kf. [Indigenous, Russia] | 8768 |
| 57 | (Abipon or Achuar or Achuagua or Akawaio or Amarizana or Andoque or Akawaio or Akuriyo or Anauya or Araona or Arawak or Ayamn or Aguaruna or Amahuaca or Amarakaeri or Andoa or Arabela or Arawak or Arhuaco or Ashaninca or Asheninca or Atsahuaca or Aymara or Ayoreo or Bakairi or "Baniva" or Barasana or Baniwa or "Baure" or Bororo or Cabiyari or Cacataibo or Caquinte or Cacua or Cahuarano or "Caiua" or "Camara Indians" or Camaracoto or Camsa or Canamari or Candoshi or Canela or Canichana or Capanahua or Carapana or Cariay or "Carib" or Carijona or Carutana or Cashibo or Cashinahua or Cawishana or Cavinena or Caxuiana or Cayuvava or Chontaquiro or Cocama or "Cubeo" or Curipaco or Chacobo or Chaima or (Chana not striatus) or Chapacura or Charrua or Chimila or Chitonahua or Chorote or Chipaya or Chiquitano or Chulupi or Carare or Coconuco or Cofan or Coreguaje or Coyaima or Chamacoco or Chamicuro or Chayahuita or Cocama or "Culina" or Culino or Cubeo or Cuiba or "Cuiva" or Cumanagoto or Curripaco or "Deni" or Desano or Embera or Guarani or Guajajara or "Guana" or Guanano or Guarayo or Guarayu or Guahibo or Guajiro or Guambiano or Guanano or Guayabero or Guarequena or Guinao or "Guana" or Gayon or Guahibo or Hixkaryana or Huachipairi or Huambisa or Huarayo or Lauanaua or Ikpeng or Ingariko or Irantxe or Itonama or Inapari or Iquito or Isconahua or Jumana or Japreria or Jirajara or Juruti or Jaqaru or Jebero or Kadiweu or Kaingang or Kamayura or Karaja or Karipuna or "Kariri" or Katukina or Kaxarari or Kayabi or Kayapo or "Kuikuro alapalo" or Kulina or "Kaiwa" or Kallawaya or "Kogui" or "Kuna" or Kaweskar or "Lule" or Macuna or Maipure or Mapuche or Mataco or Mocovi or Machinere or Machinerev or Machiguenga or Macushi or Macuna or "Madi" or Malayo or Mamainde or Manao or Mandauaca or Mandawaka or Mapidian or Mapuche or Mapidian or Maquiritare or Maquiritari or Maragua or Marawan or Mariate or Marubo or Mastanahua or Mataco or Matipuhy or "Matis" or "Matses" or Mawakua or Mawakwa or Maxakali or Mehinaku or Miranha or Moronawa or Munduruku or Movima or Muellama or Muinane or Mapoyo or "Mashco Piro" or Muniche or Nambikwara or Nocaman or Nuquini or Nomatsiguenga or Nanti or Ocaina or Omagua or Orejon or "Opon" or Pacahuara or "Paez" or Paicone or Palicur or Panare or "Pano" or "Paresi" or Paumari or "Pemon" or Pilaga or Puelche or Pauna or Pauserna or Piapoco or Piraha or Piratapuyo or Pisabo or Piaroa or "Pijao" or Piratapuyo or Paraujano or Pemon or Pemono or Piapoco or Puinave or Patamona or Poyanawa or Puinave or Puquina or Quechua or Quichua or Retuara or Resigaro or Reyesano or Sabanes or "Saliba" or Saluma or Sarave or Secoya or Selknam or "Sensi" or Shaninawa or Shapra or Sharanahua or Shebayo or Shiwiar or Shikiana or Sikiana or Siriono or Sinsiga or "Siona" or Suruwaha or Tacano or Tamanaco or Tiahuanaco or Tariano or Tehuelche or Tariano or Tatuyo or "Tembe" or "Terena" or Telembi or Ticuna or Ticuna or Tiriyo or Tiwanaku or Tiwanaku or "Torom" or "Totoro" or Tsimane or Tuberao or "Tucano" or Tunebo or Tuxinawa or Tuyuca or Uainuma or Urarina or Vilela or Waimaha or Waiampi or Waiwai or Wapishana or Waraiku or Warekena or Waura or Wayampi or Wayana or Wirina or Waimaha or Waunana or "Wiwa" or "Warao" or Wayuu or Witoto or Xavante or Xipaya or Xiriana or Xokleng or Yabaana or Yaminawa or Yaminahua or Yaruma or Yawalapiti or Yuracare or Yabarana or Yavitero or "Yine" or Yamana or Yaghan or Yucuna or Yurumangui or Yukpa or Yanesha or Yoranahua or Yagua or Yaminahua or Zaparo or Zamuco or "Trio Indians" or "More Indians" or "Bare Indians").ti,ab,kf. | 3372 |
| 58 | (("Inga" or "Maca" or "Leco" or "Mojo" or "Uro" or "Maco" or Lengua or "Toba" or "Zoe" or "Ona" or "Catio" or "Passe" or "Bari" or "Awa" or "Bora" or "Bara" or "Remo" or "Pano" or "Sape") adj3 (Indians or Indian or Indigenous or Amerindian* or Aborigin* or people or peoples or elder or elders or grandmother* or grandfather* or parent* or women or men or woman or man or child* or youth or youths or baby or babies or tribe or tribes or tribal or shaman* or native or patient*)).ti,ab,kf. | 480 |
| 59 | or/57-58 [Indigenous, South America] | 3829 |
| 60 | or/1-59 [Indigenous, Global] | 465613 |
| 61 | smoking cessation/ or smoking reduction/ or "tobacco use cessation"/ | 35747 |
| 62 | exp "Tobacco Use Cessation Devices"/ | 2807 |
| 63 | exp Smoking Cessation Agents/ | 33608 |
| 64 | Vaping/ | 4668 |
| 65 | ((smok* or tobacco* or cigar* or Vape? or vaping or e-cigarette? or e-liquid? or e-cig? or ecig? or eliquid? or ecigarette? or juul or nicotine) adj5 (quit* or ceas* or cessation* or stop* or suspend* or desist* or end* or break* or cutoff* or reduct* or reduce? or reducing or termin* or discontinu* or abstin* or dehabituat* or de-habituat*)).ti,ab,kf. | 72983 |
| 66 | (pharmaco* adj2 (smok* or tobacco* or cigar* or Vape? or vaping or e-cigarette? or e-liquid? or e-cig? or ecig? or eliquid? or ecigarette? or juul or nicotine)).ti,ab,kf. | 1476 |
| 67 | (Nicorette* or bupropion* or vareniclin* or nortriptylin* or clonidin* or chantix* or champix* or wellbutrin* or well-butrin* or Zyntabac* or Quomen* or Zyban* or Amfebutamon* or Cytisine* or baptitoxin* or cytisiniclin* or Polacrilex* or sophorin*).ti,ab,kf. | 25943 |
| 68 | (nicotine adj2 (gum* or lozenge* or patch* or spray* or tablet* or sublingual* or sub-lingual* or inhal* or strip? or microtab* or replace*)).ti,ab,kf. | 6737 |
| 69 | ((smok* or tobacco* or cigar* or Vape? or vaping or e-cigarette? or e-liquid? or e-cig? or ecig? or eliquid? or ecigarette? or juul or nicotine) adj2 (intervention* or treatment* or program* or therap*)).ti,ab,kf. | 24178 |
| 70 | or/61-68 | 126790 |
| 71 | 60 and 70 | 2608 |
| 72 | limit 71 to ed=20240101-20250528 | 164 |
| 73 | limit 71 to dt=20240101-20250528 | 221 |
| 74 | 72 or 73 | 244 |

## S 2. Embase Search

| Database: Embase Classic+Embase 1947 to 2025 May 27  Platform: Ovid  Date Searched: May 28, 2025 | | |
| --- | --- | --- |
| **#** | **Searches** | **Results** |
| 1 | exp indigenous people/ | 51023 |
| 2 | ethnopharmacology/ | 5013 |
| 3 | indigenous health care/ | 1752 |
| 4 | (traditional medicine/ or african medicine/ or exp indian traditional medicine/ or kampo medicine/ or korean medicine/ or exp latin american medicine/ or mongolian medicine/ or native american medicine/ or oriental medicine/ or tibetan medicine/ or exp traditional arabic medicine/ or vietnamese traditional medicine/) not (Chinese or China).ti,ab,kf. | 48709 |
| 5 | shamanism/ | 113 |
| 6 | ((traditional adj1 (medicine* or heal* or food* or health*)) not (Chinese or China)).ti,ab,kf. | 35008 |
| 7 | (Aboriginal? or aborigine? or aborigen* or amerindian* or amerindio* or indian? or indigene* or indigenous* or indigena* or nativo or originarios or tribe? or tribal*).ti,ab,kf. | 239969 |
| 8 | or/1-7 [Indigenous, Broad] | 325221 |
| 9 | Arctic/ or nunavut/ | 10784 |
| 10 | (Aamjiwnaang or Abenaki? or Abitibiwinni or Ahtahkakoop or Aishihik or Aklavik or Akuliarmiut* or Akwesasne* or Alert Bay or Aleut* or Alexandra Fiord or Algonqui?n* or Amadjuak or Amerind* or Amisk or Anish?na?b?e* or Aquiatulavik Point or Arctic or Armstrong settlement or Aroland or Arviat or Asimakaniseekan or Assabaska or Assiniboine or Athabasca? or Athapaskan or Atikamek* or Attawapiskat or Aundeck-Omni-Kaning or autochtone*).ti,ab,kf. | 22640 |
| 11 | (Baffin Island or Baker Lake or Barriere Lake or Batchewana or Bathurst Inlet or Beardy or Bearfoot Onondaga or Beaver Creek or Behochoko or Belcher Islands or Beothuk* or Berens River Bloodvein or Betsiamite* or Bibigo?ining or Biinjitiwabik Zaaging or Bimose or Bingwi Neyaashi or Birch Portage or Bittern Lake or Bkejwanon* or Blackfoot or Bois-brule* or Brochet or Budd's Point or Buffalo River or Bungee or Bunibonibee or Bylot Island).ti,ab,kf. | 706 |
| 12 | (Cacouna or Cambridge Bay or Canoe Lake or Canupawakpa or (Cape adj1 (Dorset or Dyer or Smith)) or Carcross or Carmacks or Carrot River or Carry the Kettle or Cayuga or Charlton Depot or Chesterfield Inlet or Chipewyan or Chipp#wa* or Chisasibi or Clyde River or Coal Harbour or Colville Lake or Coral Harbour or Coucoucache or country food* or Craig Harbour or Cree or Cypress Health).ti,ab,kf. | 1205 |
| 13 | (Dakelh or Dakota or Dauphin River or Dawson City or Day Star or Deh Cho or Deline or Dene or Denesuline or Destruction Bay or Devon Island or Dipper Rapids or Dogrib* or dokis or Dopitciwa* or Dundas Harbour or Dunne?za).ti,ab,kf. | 4300 |
| 14 | (Eabametoong or (Eagle adj (Plains or village)) or Eastmain or Eeyou Istchee or Ehdiitat or Ekuanitshit* or Elak Dase or Ellesmere Island or Ennadai or eskimo* or Esquimau* or Essipit* or ethnomedicin* or ethnopharmacol*).ti,ab,kf. | 22146 |
| 15 | ((first adj2 (nation? or people?)) or First-Nation? or Firstnation? or Fisher River or Fond du Lac or (Fort adj1 (Conger or Good Hope or Hope or Liard or McPherson or Providence or Ross or Selkirk or Simpson or Smith)) or Four Portages).ti,ab,kf. | 7846 |
| 16 | (Grandmother's Bay or Gespeg or Gesgapegiag* or Ginoogaming or Gits#an or Gjoa Haven or (God's adj1 (river or lake)) or Grise Fiord or Gwi?ch?in or Gwichya).ti,ab,kf. | 26 |
| 17 | (Haida or Haines Junction or Haisla or Hall Beach or (Hare adj1 (boy* or girl* or men or man or woman or women or people* or person or persons or band? or native* or tribe or tribal)) or Hatchet Lake or Haudenosaunee or Hay River or Hazen Camp or Heiltsuk or Huron or Huron-Wendat or Huronne-Wendat).ti,ab,kf. | 655 |
| 18 | (Han not (Chinese or China)).ti,ab,kf. | 8808 |
| 19 | (Igloolik or Igluligaarjuk or Ikaluit or Ils a la Crosse or ((indian or council) adj3 band) or Innu? or Inuit* or Inuk* or Inupiat* or Inuvik or Inuvialu* or Inuinnaqtun or Iqaluit or Iqaluktuttiaq or Iroquois or Isachsen or Island Lake or Itivimiut* or Ivujivik).ti,ab,kf. | 3492 |
| 20 | (James Bay or Joseph Bighead).ti,ab,kf. | 207 |
| 21 | (Kabapikotawangag or Kalaallit* or Kahkewistahaw or Kahnawa?ke or Kanesatake or Kasabonika Lake or Kashechewan or Kaska? or Kawacatoose or Kawawachikamach* or Kebaowek or Keeseekoowenin or Kekerten or Keno City or Keewaytinook or Kee?Way?Win or Kelsey Trail Health or Kiashke Zaaging or Kimmirut or Kingnaitmiut* or King William Island or Kinistin or Kinonjeoshtegon or Kipawa or Kipisa or Kitchenuhmaykoosib or Kitcisakik or Kitigan Zibi or Kitikmeot or Kitimat or Kitsakie or Kivalliq or Kivitoo or Konadaha Seneca or Koocheching or Ktunaxa or Kugaaruk or Kugluktuk or Kutchin* or Kuujjuaq or Kwakiutl or Kwakwaka?wakw).ti,ab,kf. | 190 |
| 22 | (La Plonge or (Lac adj (Brochet or Romanie or John or Simon or La Hache or La Ronge)) or Lake Manitoba or Lean Man or Lenape or (Little adj (Black Bear or Saskatchewan or Red River or Salmon)) or Listuguj* or Long Point First or Lucky Man).ti,ab,kf. | 61 |
| 23 | (M?Chigeeng or MacDowell Lake or Mackenzie River Basin or MacKenzie Valley or Magnetawan or Maguse River or Makaw or Makaoo or Malecite* or Maliotenam or Maliseet or Mamawetan or Mamit Innuat or Mamuitun or Manawan or Mani-Utenam or Manitoulin or Manto Sipi or Mashteuiatsh or Matachewan or Mathias Colomb or Matimekosh* or Matimekush* or Mattagami or Mawiomi or (medicine adj (man or men or woman or women)) or Metchif or Metif or metis or Miawpukek or Michif or Mic?mac or mic mac or Migmaw or Mig maw or Mi?gmaq or Mi?gmawei or Mi?kmaq or Mi#chif or Mingan or Ministikwan or Minoahchak or Mirond Lake or Mishkeegogamang or Missanabie or Mistawasis or Mistissini* or Mitaanjigaming or Mixed-blood* or Mo?hawk or Mocreebec or Montagnais or Moos-Toosis or (moravian adj2 thames) or Moraviantown or Morin Lake or Mosquito-Grizzley Bear* or Muscowpetung or Mushkegowuk or Muskoday or Muskowekwan).ti,ab,kf. | 2208 |
| 24 | ("Nacho Nyak Dun" or Naicatchewenin or Nain or Nako#a or Namaygoosisagagun or Nan#sivik or Naongashiing or Naotkamegwanning or Naskapi* or Natashquan* or (native adj2 (group? or health or community or communities or person$ or people$ or population* or america* or canad* or Nation or band or bands or reserv*)) or (Native* adj1 (man or men or women or woman or boy* or girl* or child* or adolescent* or youth? or adult*)) or Nekaneet or Nelson House or Nemaska* or Nemiscau or Neskantaga or Netsilik or New Thunderchild or Nibinamik or Nigigoonsiminikaaning or Nihtat or Nipissing or Nisga?a or Nisichawayasihk or Nlaka?pamux or Norman Wells or Northlands Nursing Station or North Slave or (northern adj1 (Saskatchewan or Manitoba or Quebec or Alberta or British Columbia or Ontario)) or Norway House or Nottingham Island or Nugumiut* or Nunatsiavut or Nunav* or Nutaqqavut or Nuwata or Nuxalk or Nuu-chah-nulth).ti,ab,kf. | 27202 |
| 25 | (ob?djiwan or Obashkaandagaang or Ocean Man or Ochapowace or O-Chi-Chak-Ko-Sipi or Ochiichagwe or Odanak or off-reserve or Oji-Cree or Ojibw* or Okanagan or Okanese or Okemasis or Oki or Old Crow or One Arrow or Oneida or Onion Lake or Onigaming or Onkwehonwe or Onodaga or on-reserve or Opaskwayak or Opawakoscikan or O-Pipon-Na-Piwin or Opitciwa* or Oqomiut* or Original people? or Ouj?-Bougoumou or Ouje?Bougoumou or Oweenkeno).ti,ab,kf. | 1355 |
| 26 | (Padlei or Padloping Island or Pakua Ship* or Pakuaship* or Pangnirtung or Parmachene or pasqua or Passamaquoddy or Pauingassi or Pauktuutit or Paulatuk or Peepeekisis or Peguis or Pelly or Perry Island or Pessamit* or Peter Ballantyne or (Peuple adj2 (premier or racine or natif*)) or Pheasant Rump or Piapot or Pikogan or Pikwakanagan or Pinaymootang or Pine Bluff or Pond Inlet or Port Burwell or Potato River or Potawatomi or Prairie North Health or premiere nation or Prince Albert Parkland Health or Pukatawagan).ti,ab,kf. | 111 |
| 27 | (Qaumauangmiut* or Qayuqtuvik or Qikiqtarjuaq or Qikqta* or Qingaumiut* or Qoloqtaaluk).ti,ab,kf. | 10 |
| 28 | (Rankin Inlet or Rapid Lake or Read Island or Red Pheasant or red road or Red Sucker Lake or Repulse Bay or residential school* or Resolute Bay or Resolution Island or Roseau River).ti,ab,kf. | 791 |
| 29 | (Sabaskong or Sachs Harbour or Sagamok or Sagkeeng or Sahtu or Sakimay or Salish or Sa?lteaux or Sandy Narrows or Sanikiluak or Sanikiluarmiut* or Sapotaweyak or Saulteaux or Saumingmiut* or Sayisi Cree or Schefferville or Sec wepmc or Seekaskootch or Sekani or Sekon or Seneca or Sept-Iles or shaman* or Shamattawa or Shawanaga or Sheguiandah or Shesheep or Sheshegwaning or Shoal Lake or Shubenacadie or Sikusilaamiut* or Sioux or Siphik or Six Nations or Skownan or Slave Lake or Slavey* or Southend or South Indian Lake or South Slave or Sunrise Health or Standing Buffalo or Stanjikoming or Stanley or Starblanket or Stl?atlimc or St Theresa Point or Sturgeon or Subarctic or Sucker River or Sweetgrass).ti,ab,kf. | 9132 |
| 30 | (Tadoule Lake or Tagish or Tahltan or Takuaikan or Talirpingmiut* or Talo?yoak or Ta?an Kwach?an or Tanana? or Tanquary Camp or Tarramiut* or Tasttine or Tataskweyak or Tavani or Taykwa Tagamou or Temagami or T#mis#aming or Teslin or Tetlitn or Thom Bay or Tlingit or Tlicho or Tli Cho or Tootinaowaziibeeng or Tr?ondek Hwech?in or treaty or treaties or Trout Lake or Tsilgehtchic or Tsilhqot?in or Tsimshian or Tsuu T?inia or Tulita or Tuktoyaktuk or Tumor Lake or Tungasugit or Turtle island or Tuscarora or Tutchone* or Tuttarvingat).ti,ab,kf. | 3418 |
| 31 | (Uashat or Ulukhaktck or Umingmaktok or Unamen Shipu or Ungava or Upper Liard or Uqqurmiut* or (urban adj3 (Indian* or Native* or Aboriginal* or indigenous*))).ti,ab,kf. | 2520 |
| 32 | (Victoria Island or Vuntut).ti,ab,kf. | 44 |
| 33 | (Waban-Aki or Wabaseemoog or Wabauskang or Wabigoon Lake or Wager Bay or Wahgoshig or Wahnapitae or Wahpeton or Wakashan or Wapachewanak or Wapekeka or Wa-Pii or Wasagamack or Wasauksing or Washagamis Bay or Waskaganish or Waswanipi or Waterhen or Watson Lake or Wauzhushk or Wawakepewin or Waywayseecappo or Webequie or Weenusk or Wemindji or Wemotaci or Wendake or Wendat* or Wet?suwet?en or Whale Cove or Whapmagoostui or (White adj (Bear or Cap)) or Whitehorse or Wikwemikong or Willow Bunch or Winneway or Witchekan or Wolastoqiyik or Wolinak or Wood Mountain or Woody Lake or Wrigley or Wuskwi or Wyandot).ti,ab,kf. | 314 |
| 34 | (Yellowkni* or Yellowquill or Yellow Quill or York Landing or Yukon).ti,ab,kf. | 1010 |
| 35 | or/9-34 [Indigenous, Canada] | 112410 |
| 36 | alaska/ | 2274 |
| 37 | ("A' ani" or Absaroka or Haaninin or Atsina or "Gros Ventre" or Acopsel or Tlacopsel or Lacopsel or Ahtna or Ahtena or Akenitsi or Occaneechi or Akokisa or Horcoquisa or Orcoquizas or Aleut or Unangax or Unangan or Alibamu or "Alabama Alsea" or Alutiiq or Sugpiag or Amahami or Awaxawi or Androscoggin or Arosaguntacook or Ameriscoggin or Anishinaabeg or Chippewa or Anihsinape or Saulteaux or Apalachee or Aranama or "Texan Coahuilteca" or Tamique or Arikara or Sahnish or Arickaree or Adakadaho or Assiniboine or Nakota or Nakoda or Nakona or "Atsa' Kudok-wa" or Awatixa or Bannock or "Snake Indian*" or Bidai or Quasmigdo or Biloxi or Blackfoot or Niitsitapi or Sikasikaitsitapi or Cahto or Kaipomo or Cahuilla or Ivilyuqaletem or Ivilyuat or Catawba or Inna or Iswa or Chemehuevi or Chickasaw or "Chilula Chimakum" or Aqokulo or Chimariko or Chiricahua or Tsokanende or Chitimacha or Chetimachan or Sitimacha or Chowanoke or Roanoke or Chumash or Ciboney or "Taino Ciwat" or Clatsop or Coos or Coosa or Uchis or Chiaha or Coste or Talisi or Coquille or Kokwell or Coso or Cowlitz or Taitnapam or "Crow Nation" or "Cui Ui Ticutta" or Cupeno or Kuupangaxwichem or Cupa or "Cup' ig" or Nunivak or "Dakota Oyate" or Lakota or Nakota or Santee or Teton or Sioux or Deadose or "Deg Xina" or "Deg Xit' an" or Kaiyuhkhotana or "Deg Hit' an" or "Dena' ina" or Tanaina or "Dichinanek' Hwt' ana" or "Upper Kuskokwim Athabascan*" or Kolchan or Goltsan or "Tundra Kolosh" or "Do lkabya" or Duwamish or Esselen or Eyak or "Gidi' tikadi" or Guwevkabaya or "Gwich' in" or Kutchin or Haida or Xaadas or Xaat or Halchidhoma or Havasupai or "Green Water People" or Hiratsa or Hiraaca or "Ho-chaaqa" or Winnebago or Holikachuk or Innoko or "Tlegon-khotana" or Hopi or "Houma-Louisiana" or Huaco or Waco or Hualapai or Hupa or Natinixwe or "Natinook-wa" or "Hwech' in" or Hankutchin or "Iroquois Confederacy" or "Hodinoso ni" or "Illinois Confedera*" or Ilinoweg or Illini or Inupiat or Inuit or Ioway or Baxoje or Jicarilla or Juaneno or Acjachemen or Jumano or Kalapuya or Clackama or Kalispel or "Pend d' Oreilles" or Qlispe or Karuk or Karok or "Chum-ne" or Katkoc or Kansa or Kanza or Kawaiisu or Nuwa or Kennebec or "Kinipekw Kittitas" or Klickitat or "Qwu' lh-hwai-pum" or "Awi-adshi" or Mahane or Wahnookt or "Koa' aga' itoka" or Keresan or Kichai or Kitsai or Keechi or "K' itaish" or Kiowa or Gaigwu or Cauigu or Kutjau or "Kwu-da" or "Tep-da" or Kitanemuk or Kittitas or Klickitat or "Qwu' lh-hwai-pum" or "Awi-adshi" or Mahane or Wahnookt or "Koa' aga' itoka" or Konkow or "Koop Ticutta" or Koyukon or Ktunaxa or Kootenai or Kucadikadi or "Kotsa' va" or Kumeyaay or "Tipai-Ipai" or Kamia or Diegueno or Kwapa or Cocopah or Cucapa or "Xawitt kwnchawaay" or Lassik or Lenape or "Leni-Lenape" or Lipan or Luiseno or Payomkawichum or Madqwadabaya or "Desert Yavapai" or Mahican or Mohicans or Makah or Makuhadokado or Maliseet or Wolistoqiag or Manahoac or Mahock or Meipontsky or Mandan or Mattole or "Bear River" or "Tul' bush" or "Ni' ekeni" or Meherrin or Menominee or Mackinac or Mescalero or Myaamiaki or Kickapoo or Twigtwee or Missouria or Miwok or Miwuk or Moadokado or Modoc or Mohave or "Aha Makhav" or Mohawk or "Kaneng' hega" or Molala or Molale or Molele or Nyyhmy or Moosonee or "Moose Cree" or Monsonis or Multnomah or (Chinook not cultivar) or Nabedache or Nabaydacu or Wawadishe or Nabiltse or Dakubetede or "Nacho Nyak Dun" or Tutchone or Nacono or "Na' isha" or Nanticoke or Navajo or Ndee or Nial or Niimiipu or "Nez Perce" or Watapala or Watapahlute or Nisenan or Nisqually or Nomlaki or Noamlakee or "Central Wintun" or Nongatl or Nottoway or Cheroenhaka or "Northern Cheyenne" or Ohlone or Costanoan or Omaha or "O' odham" or Pima or Papago or Osage or Otoe or Otse or "Ozav Dika" or Palus or Passamaquoddy or Pestomuhkati or Patiri or Petaros or Pastia or Patwin or "Southern Wintun" or Panis or Skidi or Pedee or Penobscot or "Petun Piipaash" or "Kokmalik' op" or Piscatawa or Doeg or Conoy or "Pit River" or Pomo or Kashaya or Ponca or Ponka or Pottawatomi or Bodewadmik or Powhatan or Puyallup or Spuyalepabs or Quapaw or Ugahxpa or Quechan or Kwtsaan or Quileute or Salinan or Saponi or Monacan or Sapon or "Eastern Blackfoot" or Christanna or Sawawatodo or Serrano or Taaqtam or "Maarenga' yam" or Yuhaviatam or Shasta or Chasta or Sasti or Shoshone or Siletz or Sinkine or Sinkyone or "Siuslaw Umpqua" or Skitswish or "Schitsu' umash" or Snohomish or Snuqualmi or Sokoki or Missiquoi or Stillaguamish or Stoluckwamish or Suquamish or Sutaio or Swinomish or Skagit or Syilx or Sotaae or "Taga Ticutta" or Takelma or Dagelma or Taltushtuntede or Galice or "Tanan Gwich' in" or Taos or Taovaya or Tataviam or Alliklik or Tawakoni or Tahuacano or Tenino or Thawikila or Hathawekela or "Fort Ancient" or Tigua or Tillamook or Nehalem or Timbisha or Panamint or Timpanogos or Tlingit or "Toi Ticutta" or Tolowa or "Talawa Dini' " or Tongva or Gabrieleno or Fernandeno or Tobikhar or Tonkawa or Ticanwatic or Tsikip or Appalousa or Opelousa or Tsitsistas or Tubatulabal or Tukabatchee or Tuscarora or Tomahittan or Kuskarawock or Tutelo or Tutero or Totteroy or Tutera or Yusan or Tututni or Umatilla or Umpqua or Waccamaw or Waxmaw or Wadatika or "Harney Valley Paiute" or Wailiki or Waluulapam or "Walla Walla" or Walpapi or Huipui or Wampanoag or Massasoit or Wanapum or Wappo or Washoe or Wichita or Willapa or Kwalhioqua or "Wi pukba" or "Verde Valley Yavapai" or Wintu or "Northern Wintun" or Wiyot or "Wee' at" or Weyet or Yakama or "Yamosopo Tuviwarai" or Yaqui or Yoeme or Yatasi or Yattasih or "Yavbe' " or "Yavapai" or "Ysleta del Sur" or Yojuane or Yokuts or Mariposa or Yuki or Yupighyt or "Yup'ik" or Yupik or Yurok or "Olekwo'l" or Zuni or "Native Hawaiian*" or Kanaka or Maoli or Oiwi).ti,ab,kf. | 20245 |
| 38 | ((Applegate or Alabama or Delaware or Flathead or Hohe or Iowa or Ishak or Kaw or Kato or Spokane or Miami or Arkansas or Tali or Tunica or Pawnee or Okanagan or "Coeur D' Alene" or Piscataway or Ree or Tula or Wichita or Yuma) adj3 (reservation* or nation or people or peoples or population or man or men or woman or women or child* or youth* or elder or elders or communit* or tribe or tribes or tribal or Indian*)).ti,ab,kf. | 3426 |
| 39 | or/36-38 [Indigenous, United States] | 25779 |
| 40 | ("Abakuria" or "Abaluhya" or "Abagusii" or "Abakuria" or "Aembu" or "Agikuyu" or "Akamba" or "Anuak" or "Anywaa" or "Amazigh" or "Ambala" or "Ambeere" or "Ambundu" or "Ambuun" or "Amharan" or "Angba" or "Baaka" or "Baamba" or "Babindi" or "Babini" or "Baboma" or "Bachokwe" or "Bacwa" or "Bafumbira" or "Baganda" or "Bagyele" or "Bagwere" or "Bagyeli" or "Bakiga" or "Bakola" or "Baholo" or "Bakalanga" or "Bakiga" or "Bakolo" or "Bakongo" or "Bakonjo" or "Baluba" or "Balunda" or "Balovale" or "Bamasaba" or "Bambuti" or "Bangala" or "Bangoli" or "Bangungu" or "Bantu" or "Banyankole" or "Banyarwanda" or "Banyole" or "Banyoro" or "Bapende" or "Bapedi" or "Barabaig" or "Barombi" or "Barundi" or "Baruuli" or "Basamia" or "Basoga" or "Batswana" or "Batooro" or "Batsamba" or "Batswana" or "Batwa" or "Bayaka" or "Bedzan" or "Bazombe" or "Bebayaka" or "Bedzan" or "Bhaca" or "Biaka" or "Borana" or "Chewa" or "Copts" or "Cormorian" or "Cushitic" or "Dahalo" or "Datooga" or "Dikidiki" or "Dogon" or "Ewondo" or "Fulani" or "Fuliru" or "Ganguela" or "Gciriku" or "Gyele" or "Hadza" or "Hadzabe" or "Haillom" or "Haratin" or "Herero" or "Himba" or "Hlubi" or "Iriryen" or "Iqvayliyen" or "Kabyle*" or "Kalenjin" or "Kanioka" or "Kanioka" or "Kaonde" or "Karamojong" or "Kavango" or "Kereuyu" or "Khoikhoi" or "KhoiSan" or "Kikuyu Kwangali" or "Lokele" or "Lowme" or "Lotuko" or "Lwalwa" or "Maasai" or "Makonde" or "Makua" or "Mande" or "Masalit" or "Matumbi" or "Mayeuyi" or "Mayeyi" or "Mbenga" or "Mbukushu" or "Mbochi" or "Mboro" or "Mbuti" or "Medzan" or "Mijikenda" or "Mozabite*" or "Nafusa" or "Ndebele" or "Ngombe" or "Namaqua" or "Nyanga" or "Nyamwezi" or "Ogiek" or "Ovambo" or "Ovimbundu" or "Phuthi" or "Pokomo" or "Rendille" or "Riffian" or "Riffians" or "Sakuma" or "Samburu" or "Sandawe" or "Sangha" or "Sango" or "Sengwer" or "Serer" or "Sesotho" or "Shangaan" or "Shawiya" or "Shenwa" or "Shi" or "Shilluk" or "Sukua" or "Sukus" or "Swahili" or "Tabwa" or "Tambuka" or "Taveta" or "Thembu" or "Tigrayan" or "Topoke" or "Tsonga" or "Toubou" or "Tuareg" or "Tumbuka" or "Ugana" or "Wochua" or "Xhosa" or "Xindonga" or "Yoruba" or "Zenati" or "Zuwara" or (("Indigenous" or "Afar" or "Afars" or "Aka" or "Akie" or "Ameru" or "Asua" or "Ateker" or "Atwot" or "Awjila" or "Bafia" or "Baka" or "Bakongo" or "Bakwe" or "Balunda" or "Balovale" or "Bango" or "Bassa" or "Beja" or "Bekpak" or "Bemba" or "Bembe" or "Benet" or "Berber" or "Berbers" or "Bira" or "Bowe" or "Bubi" or "Budja" or "Bulu" or "Bunrun" or "Chaga" or "Chopi" or "Damara" or "Dinka" or "Djerba" or "Duala" or "Dzing" or "Efe" or "Elmolo" or "Fang" or "Foora" or "Fula" or "Fur" or "Ghomara" or "Ghadames" or "Gllana" or "Glu" or "Gogo" or "Gongo" or "Haya" or "Havu" or "Hema" or "Hima" or "Hunde" or "Hutu" or "Huva" or "Iboko" or "Igbo" or "Ijo" or "Jieng" or "Kadu" or "Kande" or "Kango" or "Katla" or "Kgaga" or "Khoe" or "Kola" or "Komo" or "Kota" or "Kua" or "Kuba" or "Kwango" or "Kx'z" or "Kxoe" or "Lala" or "Lozi" or "Luo" or "Luba" or "Lupu" or "Masmuda" or "Matmata" or "Mbala" or "Mbam" or "Mbo" or "Mbolo" or "Mbuza" or "Mongo" or "Mpondo" or "Myene" or "Naadh" or "Nama" or "Nande" or "Naro" or "Ngoni" or "Ndau" or "Ndebele" or "Ngoli" or "Ngondi" or "Ngoni" or "Nguni" or "Nkoya" or "Nkumu" or "Nuba" or "Nubian" or "Nuer" or "Nzebi" or "Ogoni" or "Omoro" or "Oroko" or "Pygmy" or "Popoi" or "Poto" or "Puru" or "Rashad" or (San not ("San Francisco" or "San Diego" or "San Antonio")) or "Sango" or "Sanhaja" or "Sena" or "Shilha" or "Shira" or "Shona" or "Shua" or "Sokna" or "Somali*" or "Sotho" or "Sua" or "Subu" or "Swazi" or "Taitaa" or "Tchokwe" or "Teke" or "Tembo" or "Tetela" or (Tonga and Africa*) or "Tshwa" or "Tsoa" or "Twa" or "Turkana" or "Tuu" or "Venda" or "Vira" or "Watta" or "Wakuti" or "Yaaku" or "Yaka" or "Yakoma" or "Yanzi" or "Yao" or "Yeke" or "Yela" or "Yeyi" or "Zulu") adj3 (population* or people or peoples or person or persons or elder or elders or man or men or woman or women or child* or youth* or clan or clans or tribe or tribes or tribal or family or families or parent* or grandparent* or elder or elders or grandmother* or grandfather* or baby or babies or infant or infants or patient or patients or speakers or speaking or village* or communit*))).ti,ab,kf. [Indigenous, Africa] | 44766 |
| 41 | "torres strait islander*".ti,ab,kf. [Indigenous, Australia] | 3958 |
| 42 | (Acatec or Aguacateco or Amuzgo or Bokota or Boruca or Bribri or "Bri Bri" or Buglere or Cabecar or Cakchiquel or Changuena or Chatino or Chiapanec or Chicomuceltec or Chinantee or Chocho or Cholti or "Ch'olti'" or "Ch'olti'anor Chontal" or Chorotega or Chorti or Chuj or Chumbia or Corobici or (Cueva not Spain) or Cuicatec or Cuitlatee or Cuytec or Dorasque or Embera or Garifuna or Guatuso or Guaymi or Guaymis or Guetar or Huastec or Huave or Huetar or Itzaj or Ixil or Jacalteco or Jonaz or Kanjobal or Kekchi or Kuna or Maleku or Mangue or Matambu or Matlatzinca or Mazahua or Motozintlec or Mayan or Mayangna or Mestizo or Miskito or Mixtec or Mopan or Nahua or Nahuatl or Ngabe or Otomi or Pantec or Paya or Popoloca or Popoloc or Poqomam or Poqomchi or "Q'eqchi'" or Quiche or Quitirrisi or Sacapulteco or Sipacapense or Subtiaba or Tacaneco or Tarasco or Tamaulipec or Tamazultec or Tecoxquin or Tectiteco or Tecual or Tecuexe or Tepehura or Tepuztecor or Teribe or Terraba or Totonac or Trique or Tzeltal or Tzotzil or Tzutujil or Ulwa or Uspantec* or Uspanteko or Voto or Xinca or Waunana or Wounaan or Yucatec or Zapotec or Zoque).ti,ab,kf. [Indigenous, Central America] | 3735 |
| 43 | (((China or Chinese) and "ethnic minorit*") or "Achang" or "Bonan" or "Bouyei" or "Blang" or "Deang" or "Dongxiang" or "Dulong" or "Ewenki" or "Gaoshan" or "Gelao" or "Hezhe" or "Jingpo" or "Jinuo" or "Kazak" or "Kirgiz" or "Korean" or "Lahu" or "Luoba" or "Manchu" or "Maonan" or "Menba" or "Miao" or "Mongolian" or "Mulao" or "Naxi" or "Oroqen" or "Ozbek" or " Pumi" or "Qiang" or "Shui" or "Tajik" or "Tatar" or "Tibetan" or "Tujia" or "Uigur" or "Yugur" or "Zhuang" or (("Hui" or "Yi" or "Dong" or "Yao" or "Bai" or "Hani" or "Li" or "Dai" or "She" or "Lisu" or "Va" or "Sui" or "Tu" or "Xibe" or "Daur" or "Salar" or "Nu" or "Gin" or "Jino") adj3 (ethnic or minorit* or population* or people or peoples or person or persons or elder or elders or man or men or woman or women or child* or youth* or clan or clans or tribe or tribes or tribal or family or families or parent* or grandparent* or elder or elders or grandmother* or grandfather* or baby or babies or infant or infants or patient or patients or speakers or speaking or village* or communit*))).ti,ab,kf. [Indigenous, China] | 159724 |
| 44 | Greenland/ | 4071 |
| 45 | (Greenland* or Kalaallit or Kalaallisut or Nuuk or Sisimiut or Ilulissat or Qaqortoq Aasiaat or Maniitsoq or Tasiilaq or Uummannaq or Narsaq or Paamiut or Nanortalik or Upernavik or Qasigiannguit or Tunumiit or Inughuit or Avanersuarmiut).ti,ab,kf. | 5347 |
| 46 | or/44-45 [Greenland] | 5879 |
| 47 | ("Adivasis" or "Adnamanese" or "Andaman" or "Baluch" or "Baluchis" or "Bodo" or "Boro" or "Boros" or "Bote" or "Brahuis" or "Chakmas" or "Chepang" or "Chhantyal" or "Damai" or "Dewan" or "Ghale" or "Gurkha" or "Gurung" or "Hayu" or "Hyolmo" or "Jarawa" or "Jirel" or "Jumma" or "Kalash" or "Khas" or "Kirati" or "Koinch" or "Kulung" or "Kusunda" or "Limbu" or "Lohorung" or "Magar" or "Makrani" or "Mangar" or "Marma" or "Miji" or "Mongar" or "Mro" or "Naga" or "Nepami" or "Newar" or "Nicobar" or "Onge" or "Rai" or "Rang" or "Raute" or "Sajolang" or "Santhal" or "Sentinelese" or "Sindhis" or "Sulemani" or "Sunuwar" or "Tamang" or "Thakali" or "Thangmi" or "Tharu" or "Tripura" or "Tumbahangphe" or "Wanniyala-Aetto" or "Yakkha" or "Yolmopa").ti,ab,kf. [Indigenous, Indian Subcontinent] | 15244 |
| 48 | (Aguacatec or Akwa'ala or Abxubal or Ayuukja'ay or "Batzil k'op" or Binizaa or "Chichimeca Jonaz" or Chinantec or Chocho or "Ch ol" or Chontal or "Chuj" or Cochimi or Comcaac or Hamasipini or Harijio or "Ha shuta enima" or "Hach t'an" or Huastecor Hnahnu or Hnatho or "indos mexicano" or Ixcatec or Ixil or Jacaltec or K'akchikel or K'anjobal or Kanjobal or Kaqchikel or Kechi or K'iche or Kikapooa or Kikapu or Kiliwa or "Ko'lew" or "K'op o winik atel" or Kumiai or Lacandon or Laymon or Makurawe or Maya or Maya'wiinik or Mazahua or Mazatec or Me'phaa or Mesoamerindian* or Mexicanero or Mexikatlajtolli or "Mixe" or Mixtec or Motocintleco or Mti'pa or Nahuas or Ocuiltec or Otomi or (Oaxaca not Oaxaca-Blinder) or "Pame" or Papago or Tlahuica or Paipai or "Pima Bajo" or "pueblos indigenas" or Purepecha or P'urhepecha or Qatok or (Quiche not Guatemala) or Q'iche or Raramuri or "Runixa ngiigua" or ("Seri" not "Seri 82") or "Slijuala sihanuk" or Tacuate or Tarahumara or Teenek or Tepehua or Ti'pai or Tlapanec or "Tohono O'odham" or Totonac or Tachiwin or "Tsa jujmi" or Tzotzil or "Tu'un savi" or Tzeltal or "Uza" or "Winik" or Xigue or Yucatec or Zapotec).ti,ab,kf. [Indigenous, Mexico] | 4054 |
| 49 | ("Bedouin*" or "Jahalin" or "al-Kaabneh" or "al-Azazmeh" or "al-Ramadin" or "al-Rshaida").ti,ab,kf. | 1502 |
| 50 | ((nomadic or seminomad* or "semi nomad*") adj3 ("people" or "peoples" or "elder" or "elders" or "grandmother*" or "grandfather*" or "parent*" or "women" or "men" or "woman" or "man" or "child*" or "youth" or "youths" or "baby" or "babies" or "tribe" or "tribes" or "tribal" or "shaman*" or "native" or "patient*")).ti,ab,kf. | 334 |
| 51 | or/49-50 [Indigenous, Middle East] | 1822 |
| 52 | (maori* or maaori* or "m aori" or "ma ori" or moriori or tangata or whanau or rangatahi or tamariki or wahine or marae or hauora or kaupapa or tinana or hinengaro or wairua).ti,ab,kf. [Indigenous, New Zeland] | 6880 |
| 53 | (Saami or Sampi or (Sami not Ulus) or Samis or Southernsami* or Umesami* or Pitesami* or Lulesami* or Northernsami* or Enaresami* or Kolasami* or Lapp or Lapps or Lappish or Lappland or (Lapland* not longspur) or Lappalainen* or Saamelainen* or reindeer herd* or reindeer culture* or reindeer pastoral* or Lappbys or Samebys or reinbeitesdistrikt or paliskunta or siida).ti,ab,kf. [Indigenous, Northern Europe] | 1872 |
| 54 | (Ainus or Ainu or Aleuts or Alyutors or Chukchis or Chuvans or Dolgans or Enets or Entsy or Yupik or Yup'ik or Yuit or Yupigyt or Chaplino or Naukan or Itelmens or Kamchadals or Kereks or "Komi" or Koryaks or Nenets or Nentsy or Nganasans or Tavgi or Sami or Veps or Yukaghirs or Chulyms or Evenks or Tungus or Evens or "Kets" or Khantys or Mansi or Vguls or Selkups or Teleuts or Nanais or Nanaitsy or Negidal or Nivikh or Oroch or orok or Taz or udege or ulch or Kumadins or Chelkans or Shorians or Soyots or Telengits or Tofalars or Tugalars or "Tufans" or "Todzhins" or Laks or Tabasarans or Turuls or Aguls or Tsakhurs or Kumyks or Nogais or "Andis" or Akhvakh or Archins or Bagvalals or Bezhta or Botlikhs or Chamalals or Godoberi or Hinukh or Hunzibs or Khwarshi or Karata or Tindis or Tsez or Abazin or Besermyan or Izhorians or Karelians or Nagaybaks or Setos or Shapsugs or Quratay).ti,ab,kf. [Indigenous, Russia] | 13093 |
| 55 | (Abipon or Achuar or Achuagua or Akawaio or Amarizana or Andoque or Akawaio or Akuriyo or Anauya or Araona or Arawak or Ayamn or Aguaruna or Amahuaca or Amarakaeri or Andoa or Arabela or Arawak or Arhuaco or Ashaninca or Asheninca or Atsahuaca or Aymara or Ayoreo or Bakairi or "Baniva" or Barasana or Baniwa or "Baure" or Bororo or Cabiyari or Cacataibo or Caquinte or Cacua or Cahuarano or "Caiua" or "Camara Indians" or Camaracoto or Camsa or Canamari or Candoshi or Canela or Canichana or Capanahua or Carapana or Cariay or "Carib" or Carijona or Carutana or Cashibo or Cashinahua or Cawishana or Cavinena or Caxuiana or Cayuvava or Chontaquiro or Cocama or "Cubeo" or Curipaco or Chacobo or Chaima or (Chana not striatus) or Chapacura or Charrua or Chimila or Chitonahua or Chorote or Chipaya or Chiquitano or Chulupi or Carare or Coconuco or Cofan or Coreguaje or Coyaima or Chamacoco or Chamicuro or Chayahuita or Cocama or "Culina" or Culino or Cubeo or Cuiba or "Cuiva" or Cumanagoto or Curripaco or "Deni" or Desano or Embera or Guarani or Guajajara or "Guana" or Guanano or Guarayo or Guarayu or Guahibo or Guajiro or Guambiano or Guanano or Guayabero or Guarequena or Guinao or "Guana" or Gayon or Guahibo or Hixkaryana or Huachipairi or Huambisa or Huarayo or Lauanaua or Ikpeng or Ingariko or Irantxe or Itonama or Inapari or Iquito or Isconahua or Jumana or Japreria or Jirajara or Juruti or Jaqaru or Jebero or Kadiweu or Kaingang or Kamayura or Karaja or Karipuna or "Kariri" or Katukina or Kaxarari or Kayabi or Kayapo or "Kuikuro alapalo" or Kulina or "Kaiwa" or Kallawaya or "Kogui" or "Kuna" or Kaweskar or "Lule" or Macuna or Maipure or Mapuche or Mataco or Mocovi or Machinere or Machinerev or Machiguenga or Macushi or Macuna or "Madi" or Malayo or Mamainde or Manao or Mandauaca or Mandawaka or Mapidian or Mapuche or Mapidian or Maquiritare or Maquiritari or Maragua or Marawan or Mariate or Marubo or Mastanahua or Mataco or Matipuhy or "Matis" or "Matses" or Mawakua or Mawakwa or Maxakali or Mehinaku or Miranha or Moronawa or Munduruku or Movima or Muellama or Muinane or Mapoyo or "Mashco Piro" or Muniche or Nambikwara or Nocaman or Nuquini or Nomatsiguenga or Nanti or Ocaina or Omagua or Orejon or "Opon" or Pacahuara or "Paez" or Paicone or Palicur or Panare or "Pano" or "Paresi" or Paumari or "Pemon" or Pilaga or Puelche or Pauna or Pauserna or Piapoco or Piraha or Piratapuyo or Pisabo or Piaroa or "Pijao" or Piratapuyo or Paraujano or Pemon or Pemono or Piapoco or Puinave or Patamona or Poyanawa or Puinave or Puquina or Quechua or Quichua or Retuara or Resigaro or Reyesano or Sabanes or "Saliba" or Saluma or Sarave or Secoya or Selknam or "Sensi" or Shaninawa or Shapra or Sharanahua or Shebayo or Shiwiar or Shikiana or Sikiana or Siriono or Sinsiga or "Siona" or Suruwaha or Tacano or Tamanaco or Tiahuanaco or Tariano or Tehuelche or Tariano or Tatuyo or "Tembe" or "Terena" or Telembi or Ticuna or Ticuna or Tiriyo or Tiwanaku or Tiwanaku or "Torom" or "Totoro" or Tsimane or Tuberao or "Tucano" or Tunebo or Tuxinawa or Tuyuca or Uainuma or Urarina or Vilela or Waimaha or Waiampi or Waiwai or Wapishana or Waraiku or Warekena or Waura or Wayampi or Wayana or Wirina or Waimaha or Waunana or "Wiwa" or "Warao" or Wayuu or Witoto or Xavante or Xipaya or Xiriana or Xokleng or Yabaana or Yaminawa or Yaminahua or Yaruma or Yawalapiti or Yuracare or Yabarana or Yavitero or "Yine" or Yamana or Yaghan or Yucuna or Yurumangui or Yukpa or Yanesha or Yoranahua or Yagua or Yaminahua or Zaparo or Zamuco or "Trio Indians" or "More Indians" or "Bare Indians").ti,ab,kf. | 4760 |
| 56 | (("Inga" or "Maca" or "Leco" or "Mojo" or "Uro" or "Maco" or Lengua or "Toba" or "Zoe" or "Ona" or "Catio" or "Passe" or "Bari" or "Awa" or "Bora" or "Bara" or "Remo" or "Pano" or "Sape") adj3 (Indians or Indian or Indigenous or Amerindian* or Aborigin* or people or peoples or elder or elders or grandmother* or grandfather* or parent* or women or men or woman or man or child* or youth or youths or baby or babies or tribe or tribes or tribal or shaman* or native or patient*)).ti,ab,kf. | 983 |
| 57 | or/55-56 [Indigenous, South America] | 5709 |
| 58 | or/1-57 [Indigenous, Global] | 638110 |
| 59 | smoking cessation/ or smoking reduction/ | 76662 |
| 60 | electronic cigarette/ or nicotine gum/ or nicotine lozenge/ or nicotine patch/ or nicotine vaccine/ | 20103 |
| 61 | smoking cessation agent/ or *amfebutamone/ or *varenicline/ or *nortriptyline/ or *clonidine/ or *cytisine/ | 29987 |
| 62 | vaping/ | 7807 |
| 63 | ((smok* or tobacco* or cigar* or Vape? or vaping or e-cigarette? or e-liquid? or e-cig? or ecig? or eliquid? or ecigarette? or juul or nicotine) adj5 (quit* or ceas* or cessation* or stop* or suspend* or desist* or end* or break* or cutoff* or reduct* or reduce? or reducing or termin* or discontinu* or abstin* or dehabituat* or de-habituat*)).ti,ab,kf. | 98120 |
| 64 | (pharmaco* adj2 (smok* or tobacco* or cigar* or Vape? or vaping or e-cigarette? or e-liquid? or e-cig? or ecig? or eliquid? or ecigarette? or juul or nicotine)).ti,ab,kf. | 1960 |
| 65 | (Nicorette* or bupropion* or vareniclin* or nortriptylin* or clonidin* or chantix* or champix* or wellbutrin* or well-butrin* or Zyntabac* or Quomen* or Zyban* or Amfebutamon* or Cytisine* or baptitoxin* or cytisiniclin* or Polacrilex* or sophorin*).ti,ab,kf. | 36301 |
| 66 | (nicotine adj2 (gum* or lozenge* or patch* or spray* or tablet* or sublingual* or sub-lingual* or inhal* or strip? or microtab* or replace*)).ti,ab,kf. | 9263 |
| 67 | ((smok* or tobacco* or cigar* or Vape? or vaping or e-cigarette? or e-liquid? or e-cig? or ecig? or eliquid? or ecigarette? or juul or nicotine) adj2 (intervention* or treatment* or program* or therap*)).ti,ab,kf. | 32550 |
| 68 | or/59-66 | 175649 |
| 69 | 58 and 68 | 4011 |
| 70 | limit 69 to dc=20240101-20250528 | 487 |
| 71 | limit 69 to dd=20240101-20250528 | 464 |
| 72 | 70 or 71 | 488 |

## S3. CENTRAL Search

| Database: EBM Reviews - Cochrane Central Register of Controlled Trials April 2025  Platform: Ovid  Date Searched: May 28, 2025 | | |
| --- | --- | --- |
| **#** | **Searches** | **Results** |
| 1 | exp Indigenous Peoples/ | 23 |
| 2 | Ethnopharmacology/ | 10 |
| 3 | Health Services, Indigenous/ | 88 |
| 4 | exp Medicine, Traditional/ not (Chinese or China).ti,ab,kw. | 1068 |
| 5 | Shamanism/ | 2 |
| 6 | ((traditional adj1 (medicine* or heal* or food* or health*)) not (Chinese or China)).ti,ab,kw. | 1416 |
| 7 | (Aboriginal? or aborigine? or aborigen* or amerindian* or amerindio* or indian? or indigene* or indigenous* or indigena* or nativo or originarios or tribe? or tribal*).ti,ab,kw. | 8202 |
| 8 | or/1-7 [Indigenous, Broad] | 10521 |
| 9 | Arctic Regions/ or Nunavut/ | 18 |
| 10 | (Aamjiwnaang or Abenaki? or Abitibiwinni or Ahtahkakoop or Aishihik or Aklavik or Akuliarmiut* or Akwesasne* or Alert Bay or Aleut* or Alexandra Fiord or Algonqui?n* or Amadjuak or Amerind* or Amisk or Anish?na?b?e* or Aquiatulavik Point or Arctic or Armstrong settlement or Aroland or Arviat or Asimakaniseekan or Assabaska or Assiniboine or Athabasca? or Athapaskan or Atikamek* or Attawapiskat or Aundeck-Omni-Kaning or autochtone*).ti,ab,kw. | 287 |
| 11 | (Baffin Island or Baker Lake or Barriere Lake or Batchewana or Bathurst Inlet or Beardy or Bearfoot Onondaga or Beaver Creek or Behochoko or Belcher Islands or Beothuk* or Berens River Bloodvein or Betsiamite* or Bibigo?ining or Biinjitiwabik Zaaging or Bimose or Bingwi Neyaashi or Birch Portage or Bittern Lake or Bkejwanon* or Blackfoot or Bois-brule* or Brochet or Budd's Point or Buffalo River or Bungee or Bunibonibee or Bylot Island).ti,ab,kw. | 8 |
| 12 | (Cacouna or Cambridge Bay or Canoe Lake or Canupawakpa or (Cape adj1 (Dorset or Dyer or Smith)) or Carcross or Carmacks or Carrot River or Carry the Kettle or Cayuga or Charlton Depot or Chesterfield Inlet or Chipewyan or Chipp#wa* or Chisasibi or Clyde River or Coal Harbour or Colville Lake or Coral Harbour or Coucoucache or country food* or Craig Harbour or Cree or Cypress Health).ti,ab,kw. | 37 |
| 13 | (Dakelh or Dakota or Dauphin River or Dawson City or Day Star or Deh Cho or Deline or Dene or Denesuline or Destruction Bay or Devon Island or Dipper Rapids or Dogrib* or dokis or Dopitciwa* or Dundas Harbour or Dunne?za).ti,ab,kw. | 84 |
| 14 | (Eabametoong or (Eagle adj (Plains or village)) or Eastmain or Eeyou Istchee or Ehdiitat or Ekuanitshit* or Elak Dase or Ellesmere Island or Ennadai or eskimo* or Esquimau* or Essipit* or ethnomedicin* or ethnopharmacol*).ti,ab,kw. | 246 |
| 15 | ((first adj2 (nation? or people?)) or First-Nation? or Firstnation? or Fisher River or Fond du Lac or (Fort adj1 (Conger or Good Hope or Hope or Liard or McPherson or Providence or Ross or Selkirk or Simpson or Smith)) or Four Portages).ti,ab,kw. | 603 |
| 16 | (Grandmother's Bay or Gespeg or Gesgapegiag* or Ginoogaming or Gits#an or Gjoa Haven or (God's adj1 (river or lake)) or Grise Fiord or Gwi?ch?in or Gwichya).ti,ab,kw. | 1 |
| 17 | (Haida or Haines Junction or Haisla or Hall Beach or (Hare adj1 (boy* or girl* or men or man or woman or women or people* or person or persons or band? or native* or tribe or tribal)) or Hatchet Lake or Haudenosaunee or Hay River or Hazen Camp or Heiltsuk or Huron or Huron-Wendat or Huronne-Wendat).ti,ab,kw. | 7 |
| 18 | (Han not (Chinese or China)).ti,ab,kw. | 482 |
| 19 | (Igloolik or Igluligaarjuk or Ikaluit or Ils a la Crosse or ((indian or council) adj3 band) or Innu? or Inuit* or Inuk* or Inupiat* or Inuvik or Inuvialu* or Inuinnaqtun or Iqaluit or Iqaluktuttiaq or Iroquois or Isachsen or Island Lake or Itivimiut* or Ivujivik).ti,ab,kw. | 62 |
| 20 | (James Bay or Joseph Bighead).ti,ab,kw. | 1 |
| 21 | (Kabapikotawangag or Kalaallit* or Kahkewistahaw or Kahnawa?ke or Kanesatake or Kasabonika Lake or Kashechewan or Kaska? or Kawacatoose or Kawawachikamach* or Kebaowek or Keeseekoowenin or Kekerten or Keno City or Keewaytinook or Kee?Way?Win or Kelsey Trail Health or Kiashke Zaaging or Kimmirut or Kingnaitmiut* or King William Island or Kinistin or Kinonjeoshtegon or Kipawa or Kipisa or Kitchenuhmaykoosib or Kitcisakik or Kitigan Zibi or Kitikmeot or Kitimat or Kitsakie or Kivalliq or Kivitoo or Konadaha Seneca or Koocheching or Ktunaxa or Kugaaruk or Kugluktuk or Kutchin* or Kuujjuaq or Kwakiutl or Kwakwaka?wakw).ti,ab,kw. | 2 |
| 22 | (La Plonge or (Lac adj (Brochet or Romanie or John or Simon or La Hache or La Ronge)) or Lake Manitoba or Lean Man or Lenape or (Little adj (Black Bear or Saskatchewan or Red River or Salmon)) or Listuguj* or Long Point First or Lucky Man).ti,ab,kw. | 2 |
| 23 | (M?Chigeeng or MacDowell Lake or Mackenzie River Basin or MacKenzie Valley or Magnetawan or Maguse River or Makaw or Makaoo or Malecite* or Maliotenam or Maliseet or Mamawetan or Mamit Innuat or Mamuitun or Manawan or Mani-Utenam or Manitoulin or Manto Sipi or Mashteuiatsh or Matachewan or Mathias Colomb or Matimekosh* or Matimekush* or Mattagami or Mawiomi or (medicine adj (man or men or woman or women)) or Metchif or Metif or metis or Miawpukek or Michif or Mic?mac or mic mac or Migmaw or Mig maw or Mi?gmaq or Mi?gmawei or Mi?kmaq or Mi#chif or Mingan or Ministikwan or Minoahchak or Mirond Lake or Mishkeegogamang or Missanabie or Mistawasis or Mistissini* or Mitaanjigaming or Mixed-blood* or Mo?hawk or Mocreebec or Montagnais or Moos-Toosis or (moravian adj2 thames) or Moraviantown or Morin Lake or Mosquito-Grizzley Bear* or Muscowpetung or Mushkegowuk or Muskoday or Muskowekwan).ti,ab,kw. | 58 |
| 24 | ("Nacho Nyak Dun" or Naicatchewenin or Nain or Nako#a or Namaygoosisagagun or Nan#sivik or Naongashiing or Naotkamegwanning or Naskapi* or Natashquan* or (native adj2 (group? or health or community or communities or person$ or people$ or population* or america* or canad* or Nation or band or bands or reserv*)) or (Native* adj1 (man or men or women or woman or boy* or girl* or child* or adolescent* or youth? or adult*)) or Nekaneet or Nelson House or Nemaska* or Nemiscau or Neskantaga or Netsilik or New Thunderchild or Nibinamik or Nigigoonsiminikaaning or Nihtat or Nipissing or Nisga?a or Nisichawayasihk or Nlaka?pamux or Norman Wells or Northlands Nursing Station or North Slave or (northern adj1 (Saskatchewan or Manitoba or Quebec or Alberta or British Columbia or Ontario)) or Norway House or Nottingham Island or Nugumiut* or Nunatsiavut or Nunav* or Nutaqqavut or Nuwata or Nuxalk or Nuu-chah-nulth).ti,ab,kw. | 758 |
| 25 | (ob?djiwan or Obashkaandagaang or Ocean Man or Ochapowace or O-Chi-Chak-Ko-Sipi or Ochiichagwe or Odanak or off-reserve or Oji-Cree or Ojibw* or Okanagan or Okanese or Okemasis or Oki or Old Crow or One Arrow or Oneida or Onion Lake or Onigaming or Onkwehonwe or Onodaga or on-reserve or Opaskwayak or Opawakoscikan or O-Pipon-Na-Piwin or Opitciwa* or Oqomiut* or Original people? or Ouj?-Bougoumou or Ouje?Bougoumou or Oweenkeno).ti,ab,kw. | 48 |
| 26 | (Padlei or Padloping Island or Pakua Ship* or Pakuaship* or Pangnirtung or Parmachene or pasqua or Passamaquoddy or Pauingassi or Pauktuutit or Paulatuk or Peepeekisis or Peguis or Pelly or Perry Island or Pessamit* or Peter Ballantyne or (Peuple adj2 (premier or racine or natif*)) or Pheasant Rump or Piapot or Pikogan or Pikwakanagan or Pinaymootang or Pine Bluff or Pond Inlet or Port Burwell or Potato River or Potawatomi or Prairie North Health or premiere nation or Prince Albert Parkland Health or Pukatawagan).ti,ab,kw. | 11 |
| 27 | (Qaumauangmiut* or Qayuqtuvik or Qikiqtarjuaq or Qikqta* or Qingaumiut* or Qoloqtaaluk).ti,ab,kw. | 0 |
| 28 | (Rankin Inlet or Rapid Lake or Read Island or Red Pheasant or red road or Red Sucker Lake or Repulse Bay or residential school* or Resolute Bay or Resolution Island or Roseau River).ti,ab,kw. | 52 |
| 29 | (Sabaskong or Sachs Harbour or Sagamok or Sagkeeng or Sahtu or Sakimay or Salish or Sa?lteaux or Sandy Narrows or Sanikiluak or Sanikiluarmiut* or Sapotaweyak or Saulteaux or Saumingmiut* or Sayisi Cree or Schefferville or Sec wepmc or Seekaskootch or Sekani or Sekon or Seneca or Sept-Iles or shaman* or Shamattawa or Shawanaga or Sheguiandah or Shesheep or Sheshegwaning or Shoal Lake or Shubenacadie or Sikusilaamiut* or Sioux or Siphik or Six Nations or Skownan or Slave Lake or Slavey* or Southend or South Indian Lake or South Slave or Sunrise Health or Standing Buffalo or Stanjikoming or Stanley or Starblanket or Stl?atlimc or St Theresa Point or Sturgeon or Subarctic or Sucker River or Sweetgrass).ti,ab,kw. | 342 |
| 30 | (Tadoule Lake or Tagish or Tahltan or Takuaikan or Talirpingmiut* or Talo?yoak or Ta?an Kwach?an or Tanana? or Tanquary Camp or Tarramiut* or Tasttine or Tataskweyak or Tavani or Taykwa Tagamou or Temagami or T#mis#aming or Teslin or Tetlitn or Thom Bay or Tlingit or Tlicho or Tli Cho or Tootinaowaziibeeng or Tr?ondek Hwech?in or treaty or treaties or Trout Lake or Tsilgehtchic or Tsilhqot?in or Tsimshian or Tsuu T?inia or Tulita or Tuktoyaktuk or Tumor Lake or Tungasugit or Turtle island or Tuscarora or Tutchone* or Tuttarvingat).ti,ab,kw. | 30 |
| 31 | (Uashat or Ulukhaktck or Umingmaktok or Unamen Shipu or Ungava or Upper Liard or Uqqurmiut* or (urban adj3 (Indian* or Native* or Aboriginal* or indigenous*))).ti,ab,kw. | 123 |
| 32 | (Victoria Island or Vuntut).ti,ab,kw. | 2 |
| 33 | (Waban-Aki or Wabaseemoog or Wabauskang or Wabigoon Lake or Wager Bay or Wahgoshig or Wahnapitae or Wahpeton or Wakashan or Wapachewanak or Wapekeka or Wa-Pii or Wasagamack or Wasauksing or Washagamis Bay or Waskaganish or Waswanipi or Waterhen or Watson Lake or Wauzhushk or Wawakepewin or Waywayseecappo or Webequie or Weenusk or Wemindji or Wemotaci or Wendake or Wendat* or Wet?suwet?en or Whale Cove or Whapmagoostui or (White adj (Bear or Cap)) or Whitehorse or Wikwemikong or Willow Bunch or Winneway or Witchekan or Wolastoqiyik or Wolinak or Wood Mountain or Woody Lake or Wrigley or Wuskwi or Wyandot).ti,ab,kw. | 35 |
| 34 | (Yellowkni* or Yellowquill or Yellow Quill or York Landing or Yukon).ti,ab,kw. | 39 |
| 35 | or/9-34 [Indigenous, Canada] | 3131 |
| 36 | Alaska/ | 84 |
| 37 | ("A' ani" or Absaroka or Haaninin or Atsina or "Gros Ventre" or Acopsel or Tlacopsel or Lacopsel or Ahtna or Ahtena or Akenitsi or Occaneechi or Akokisa or Horcoquisa or Orcoquizas or Aleut or Unangax or Unangan or Alibamu or "Alabama Alsea" or Alutiiq or Sugpiag or Amahami or Awaxawi or Androscoggin or Arosaguntacook or Ameriscoggin or Anishinaabeg or Chippewa or Anihsinape or Saulteaux or Apalachee or Aranama or "Texan Coahuilteca" or Tamique or Arikara or Sahnish or Arickaree or Adakadaho or Assiniboine or Nakota or Nakoda or Nakona or "Atsa' Kudok-wa" or Awatixa or Bannock or "Snake Indian*" or Bidai or Quasmigdo or Biloxi or Blackfoot or Niitsitapi or Sikasikaitsitapi or Cahto or Kaipomo or Cahuilla or Ivilyuqaletem or Ivilyuat or Catawba or Inna or Iswa or Chemehuevi or Chickasaw or "Chilula Chimakum" or Aqokulo or Chimariko or Chiricahua or Tsokanende or Chitimacha or Chetimachan or Sitimacha or Chowanoke or Roanoke or Chumash or Ciboney or "Taino Ciwat" or Clatsop or Coos or Coosa or Uchis or Chiaha or Coste or Talisi or Coquille or Kokwell or Coso or Cowlitz or Taitnapam or "Crow Nation" or "Cui Ui Ticutta" or Cupeno or Kuupangaxwichem or Cupa or "Cup' ig" or Nunivak or "Dakota Oyate" or Lakota or Nakota or Santee or Teton or Sioux or Deadose or "Deg Xina" or "Deg Xit' an" or Kaiyuhkhotana or "Deg Hit' an" or "Dena' ina" or Tanaina or "Dichinanek' Hwt' ana" or "Upper Kuskokwim Athabascan*" or Kolchan or Goltsan or "Tundra Kolosh" or "Do lkabya" or Duwamish or Esselen or Eyak or "Gidi' tikadi" or Guwevkabaya or "Gwich' in" or Kutchin or Haida or Xaadas or Xaat or Halchidhoma or Havasupai or "Green Water People" or Hiratsa or Hiraaca or "Ho-chaaqa" or Winnebago or Holikachuk or Innoko or "Tlegon-khotana" or Hopi or "Houma-Louisiana" or Huaco or Waco or Hualapai or Hupa or Natinixwe or "Natinook-wa" or "Hwech' in" or Hankutchin or "Iroquois Confederacy" or "Hodinoso ni" or "Illinois Confedera*" or Ilinoweg or Illini or Inupiat or Inuit or Ioway or Baxoje or Jicarilla or Juaneno or Acjachemen or Jumano or Kalapuya or Clackama or Kalispel or "Pend d' Oreilles" or Qlispe or Karuk or Karok or "Chum-ne" or Katkoc or Kansa or Kanza or Kawaiisu or Nuwa or Kennebec or "Kinipekw Kittitas" or Klickitat or "Qwu' lh-hwai-pum" or "Awi-adshi" or Mahane or Wahnookt or "Koa' aga' itoka" or Keresan or Kichai or Kitsai or Keechi or "K' itaish" or Kiowa or Gaigwu or Cauigu or Kutjau or "Kwu-da" or "Tep-da" or Kitanemuk or Kittitas or Klickitat or "Qwu' lh-hwai-pum" or "Awi-adshi" or Mahane or Wahnookt or "Koa' aga' itoka" or Konkow or "Koop Ticutta" or Koyukon or Ktunaxa or Kootenai or Kucadikadi or "Kotsa' va" or Kumeyaay or "Tipai-Ipai" or Kamia or Diegueno or Kwapa or Cocopah or Cucapa or "Xawitt kwnchawaay" or Lassik or Lenape or "Leni-Lenape" or Lipan or Luiseno or Payomkawichum or Madqwadabaya or "Desert Yavapai" or Mahican or Mohicans or Makah or Makuhadokado or Maliseet or Wolistoqiag or Manahoac or Mahock or Meipontsky or Mandan or Mattole or "Bear River" or "Tul' bush" or "Ni' ekeni" or Meherrin or Menominee or Mackinac or Mescalero or Myaamiaki or Kickapoo or Twigtwee or Missouria or Miwok or Miwuk or Moadokado or Modoc or Mohave or "Aha Makhav" or Mohawk or "Kaneng' hega" or Molala or Molale or Molele or Nyyhmy or Moosonee or "Moose Cree" or Monsonis or Multnomah or (Chinook not cultivar) or Nabedache or Nabaydacu or Wawadishe or Nabiltse or Dakubetede or "Nacho Nyak Dun" or Tutchone or Nacono or "Na' isha" or Nanticoke or Navajo or Ndee or Nial or Niimiipu or "Nez Perce" or Watapala or Watapahlute or Nisenan or Nisqually or Nomlaki or Noamlakee or "Central Wintun" or Nongatl or Nottoway or Cheroenhaka or "Northern Cheyenne" or Ohlone or Costanoan or Omaha or "O' odham" or Pima or Papago or Osage or Otoe or Otse or "Ozav Dika" or Palus or Passamaquoddy or Pestomuhkati or Patiri or Petaros or Pastia or Patwin or "Southern Wintun" or Panis or Skidi or Pedee or Penobscot or "Petun Piipaash" or "Kokmalik' op" or Piscatawa or Doeg or Conoy or "Pit River" or Pomo or Kashaya or Ponca or Ponka or Pottawatomi or Bodewadmik or Powhatan or Puyallup or Spuyalepabs or Quapaw or Ugahxpa or Quechan or Kwtsaan or Quileute or Salinan or Saponi or Monacan or Sapon or "Eastern Blackfoot" or Christanna or Sawawatodo or Serrano or Taaqtam or "Maarenga' yam" or Yuhaviatam or Shasta or Chasta or Sasti or Shoshone or Siletz or Sinkine or Sinkyone or "Siuslaw Umpqua" or Skitswish or "Schitsu' umash" or Snohomish or Snuqualmi or Sokoki or Missiquoi or Stillaguamish or Stoluckwamish or Suquamish or Sutaio or Swinomish or Skagit or Syilx or Sotaae or "Taga Ticutta" or Takelma or Dagelma or Taltushtuntede or Galice or "Tanan Gwich' in" or Taos or Taovaya or Tataviam or Alliklik or Tawakoni or Tahuacano or Tenino or Thawikila or Hathawekela or "Fort Ancient" or Tigua or Tillamook or Nehalem or Timbisha or Panamint or Timpanogos or Tlingit or "Toi Ticutta" or Tolowa or "Talawa Dini' " or Tongva or Gabrieleno or Fernandeno or Tobikhar or Tonkawa or Ticanwatic or Tsikip or Appalousa or Opelousa or Tsitsistas or Tubatulabal or Tukabatchee or Tuscarora or Tomahittan or Kuskarawock or Tutelo or Tutero or Totteroy or Tutera or Yusan or Tututni or Umatilla or Umpqua or Waccamaw or Waxmaw or Wadatika or "Harney Valley Paiute" or Wailiki or Waluulapam or "Walla Walla" or Walpapi or Huipui or Wampanoag or Massasoit or Wanapum or Wappo or Washoe or Wichita or Willapa or Kwalhioqua or "Wi pukba" or "Verde Valley Yavapai" or Wintu or "Northern Wintun" or Wiyot or "Wee' at" or Weyet or Yakama or "Yamosopo Tuviwarai" or Yaqui or Yoeme or Yatasi or Yattasih or "Yavbe' " or "Yavapai" or "Ysleta del Sur" or Yojuane or Yokuts or Mariposa or Yuki or Yupighyt or "Yup'ik" or Yupik or Yurok or "Olekwo'l" or Zuni or "Native Hawaiian*" or Kanaka or Maoli or Oiwi).ti,ab,kw. | 1099 |
| 38 | ((Applegate or Alabama or Delaware or Flathead or Hohe or Iowa or Ishak or Kaw or Kato or Spokane or Miami or Arkansas or Tali or Tunica or Pawnee or Okanagan or "Coeur D' Alene" or Piscataway or Ree or Tula or Wichita or Yuma) adj3 (reservation* or nation or people or peoples or population or man or men or woman or women or child* or youth* or elder or elders or communit* or tribe or tribes or tribal or Indian*)).ti,ab,kw. | 188 |
| 39 | or/36-38 [Indigenous, United States] | 1363 |
| 40 | ("Abakuria" or "Abaluhya" or "Abagusii" or "Abakuria" or "Aembu" or "Agikuyu" or "Akamba" or "Anuak" or "Anywaa" or "Amazigh" or "Ambala" or "Ambeere" or "Ambundu" or "Ambuun" or "Amharan" or "Angba" or "Baaka" or "Baamba" or "Babindi" or "Babini" or "Baboma" or "Bachokwe" or "Bacwa" or "Bafumbira" or "Baganda" or "Bagyele" or "Bagwere" or "Bagyeli" or "Bakiga" or "Bakola" or "Baholo" or "Bakalanga" or "Bakiga" or "Bakolo" or "Bakongo" or "Bakonjo" or "Baluba" or "Balunda" or "Balovale" or "Bamasaba" or "Bambuti" or "Bangala" or "Bangoli" or "Bangungu" or "Bantu" or "Banyankole" or "Banyarwanda" or "Banyole" or "Banyoro" or "Bapende" or "Bapedi" or "Barabaig" or "Barombi" or "Barundi" or "Baruuli" or "Basamia" or "Basoga" or "Batswana" or "Batooro" or "Batsamba" or "Batswana" or "Batwa" or "Bayaka" or "Bedzan" or "Bazombe" or "Bebayaka" or "Bedzan" or "Bhaca" or "Biaka" or "Borana" or "Chewa" or "Copts" or "Cormorian" or "Cushitic" or "Dahalo" or "Datooga" or "Dikidiki" or "Dogon" or "Ewondo" or "Fulani" or "Fuliru" or "Ganguela" or "Gciriku" or "Gyele" or "Hadza" or "Hadzabe" or "Haillom" or "Haratin" or "Herero" or "Himba" or "Hlubi" or "Iriryen" or "Iqvayliyen" or "Kabyle*" or "Kalenjin" or "Kanioka" or "Kanioka" or "Kaonde" or "Karamojong" or "Kavango" or "Kereuyu" or "Khoikhoi" or "KhoiSan" or "Kikuyu Kwangali" or "Lokele" or "Lowme" or "Lotuko" or "Lwalwa" or "Maasai" or "Makonde" or "Makua" or "Mande" or "Masalit" or "Matumbi" or "Mayeuyi" or "Mayeyi" or "Mbenga" or "Mbukushu" or "Mbochi" or "Mboro" or "Mbuti" or "Medzan" or "Mijikenda" or "Mozabite*" or "Nafusa" or "Ndebele" or "Ngombe" or "Namaqua" or "Nyanga" or "Nyamwezi" or "Ogiek" or "Ovambo" or "Ovimbundu" or "Phuthi" or "Pokomo" or "Rendille" or "Riffian" or "Riffians" or "Sakuma" or "Samburu" or "Sandawe" or "Sangha" or "Sango" or "Sengwer" or "Serer" or "Sesotho" or "Shangaan" or "Shawiya" or "Shenwa" or "Shi" or "Shilluk" or "Sukua" or "Sukus" or "Swahili" or "Tabwa" or "Tambuka" or "Taveta" or "Thembu" or "Tigrayan" or "Topoke" or "Tsonga" or "Toubou" or "Tuareg" or "Tumbuka" or "Ugana" or "Wochua" or "Xhosa" or "Xindonga" or "Yoruba" or "Zenati" or "Zuwara" or (("Indigenous" or "Afar" or "Afars" or "Aka" or "Akie" or "Ameru" or "Asua" or "Ateker" or "Atwot" or "Awjila" or "Bafia" or "Baka" or "Bakongo" or "Bakwe" or "Balunda" or "Balovale" or "Bango" or "Bassa" or "Beja" or "Bekpak" or "Bemba" or "Bembe" or "Benet" or "Berber" or "Berbers" or "Bira" or "Bowe" or "Bubi" or "Budja" or "Bulu" or "Bunrun" or "Chaga" or "Chopi" or "Damara" or "Dinka" or "Djerba" or "Duala" or "Dzing" or "Efe" or "Elmolo" or "Fang" or "Foora" or "Fula" or "Fur" or "Ghomara" or "Ghadames" or "Gllana" or "Glu" or "Gogo" or "Gongo" or "Haya" or "Havu" or "Hema" or "Hima" or "Hunde" or "Hutu" or "Huva" or "Iboko" or "Igbo" or "Ijo" or "Jieng" or "Kadu" or "Kande" or "Kango" or "Katla" or "Kgaga" or "Khoe" or "Kola" or "Komo" or "Kota" or "Kua" or "Kuba" or "Kwango" or "Kx'z" or "Kxoe" or "Lala" or "Lozi" or "Luo" or "Luba" or "Lupu" or "Masmuda" or "Matmata" or "Mbala" or "Mbam" or "Mbo" or "Mbolo" or "Mbuza" or "Mongo" or "Mpondo" or "Myene" or "Naadh" or "Nama" or "Nande" or "Naro" or "Ngoni" or "Ndau" or "Ndebele" or "Ngoli" or "Ngondi" or "Ngoni" or "Nguni" or "Nkoya" or "Nkumu" or "Nuba" or "Nubian" or "Nuer" or "Nzebi" or "Ogoni" or "Omoro" or "Oroko" or "Pygmy" or "Popoi" or "Poto" or "Puru" or "Rashad" or (San not ("San Francisco" or "San Diego" or "San Antonio")) or "Sango" or "Sanhaja" or "Sena" or "Shilha" or "Shira" or "Shona" or "Shua" or "Sokna" or "Somali*" or "Sotho" or "Sua" or "Subu" or "Swazi" or "Taitaa" or "Tchokwe" or "Teke" or "Tembo" or "Tetela" or (Tonga and Africa*) or "Tshwa" or "Tsoa" or "Twa" or "Turkana" or "Tuu" or "Venda" or "Vira" or "Watta" or "Wakuti" or "Yaaku" or "Yaka" or "Yakoma" or "Yanzi" or "Yao" or "Yeke" or "Yela" or "Yeyi" or "Zulu") adj3 (population* or people or peoples or person or persons or elder or elders or man or men or woman or women or child* or youth* or clan or clans or tribe or tribes or tribal or family or families or parent* or grandparent* or elder or elders or grandmother* or grandfather* or baby or babies or infant or infants or patient or patients or speakers or speaking or village* or communit*))).ti,ab,kw. [Indigenous, Africa] | 1780 |
| 41 | "torres strait islander*".ti,ab,kw. [Indigenous, Australia] | 170 |
| 42 | (Acatec or Aguacateco or Amuzgo or Bokota or Boruca or Bribri or "Bri Bri" or Buglere or Cabecar or Cakchiquel or Changuena or Chatino or Chiapanec or Chicomuceltec or Chinantee or Chocho or Cholti or "Ch'olti'" or "Ch'olti'anor Chontal" or Chorotega or Chorti or Chuj or Chumbia or Corobici or (Cueva not Spain) or Cuicatec or Cuitlatee or Cuytec or Dorasque or Embera or Garifuna or Guatuso or Guaymi or Guaymis or Guetar or Huastec or Huave or Huetar or Itzaj or Ixil or Jacalteco or Jonaz or Kanjobal or Kekchi or Kuna or Maleku or Mangue or Matambu or Matlatzinca or Mazahua or Motozintlec or Mayan or Mayangna or Mestizo or Miskito or Mixtec or Mopan or Nahua or Nahuatl or Ngabe or Otomi or Pantec or Paya or Popoloca or Popoloc or Poqomam or Poqomchi or "Q'eqchi'" or Quiche or Quitirrisi or Sacapulteco or Sipacapense or Subtiaba or Tacaneco or Tarasco or Tamaulipec or Tamazultec or Tecoxquin or Tectiteco or Tecual or Tecuexe or Tepehura or Tepuztecor or Teribe or Terraba or Totonac or Trique or Tzeltal or Tzotzil or Tzutujil or Ulwa or Uspantec* or Uspanteko or Voto or Xinca or Waunana or Wounaan or Yucatec or Zapotec or Zoque).ti,ab,kw. [Indigenous, Central America] | 67 |
| 43 | exp China/ and minority group/ | 13 |
| 44 | (((China or Chinese) and "ethnic minorit*") or "Achang" or "Bonan" or "Bouyei" or "Blang" or "Deang" or "Dongxiang" or "Dulong" or "Ewenki" or "Gaoshan" or "Gelao" or "Hezhe" or "Jingpo" or "Jinuo" or "Kazak" or "Kirgiz" or "Korean" or "Lahu" or "Luoba" or "Manchu" or "Maonan" or "Menba" or "Miao" or "Mongolian" or "Mulao" or "Naxi" or "Oroqen" or "Ozbek" or " Pumi" or "Qiang" or "Shui" or "Tajik" or "Tatar" or "Tibetan" or "Tujia" or "Uigur" or "Yugur" or "Zhuang" or (("Hui" or "Yi" or "Dong" or "Yao" or "Bai" or "Hani" or "Li" or "Dai" or "She" or "Lisu" or "Va" or "Sui" or "Tu" or "Xibe" or "Daur" or "Salar" or "Nu" or "Gin" or "Jino") adj3 (ethnic or minorit* or population* or people or peoples or person or persons or elder or elders or man or men or woman or women or child* or youth* or clan or clans or tribe or tribes or tribal or family or families or parent* or grandparent* or elder or elders or grandmother* or grandfather* or baby or babies or infant or infants or patient or patients or speakers or speaking or village* or communit*))).ti,ab,kw. | 11049 |
| 45 | or/43-44 [Indigenous, China] | 11055 |
| 46 | Greenland/ | 11 |
| 47 | (Greenland* or Kalaallit or Kalaallisut or Nuuk or Sisimiut or Ilulissat or Qaqortoq Aasiaat or Maniitsoq or Tasiilaq or Uummannaq or Narsaq or Paamiut or Nanortalik or Upernavik or Qasigiannguit or Tunumiit or Inughuit or Avanersuarmiut).ti,ab,kw. | 37 |
| 48 | or/46-47 [Greenland] | 38 |
| 49 | ("Adivasis" or "Adnamanese" or "Andaman" or "Baluch" or "Baluchis" or "Bodo" or "Boro" or "Boros" or "Bote" or "Brahuis" or "Chakmas" or "Chepang" or "Chhantyal" or "Damai" or "Dewan" or "Ghale" or "Gurkha" or "Gurung" or "Hayu" or "Hyolmo" or "Jarawa" or "Jirel" or "Jumma" or "Kalash" or "Khas" or "Kirati" or "Koinch" or "Kulung" or "Kusunda" or "Limbu" or "Lohorung" or "Magar" or "Makrani" or "Mangar" or "Marma" or "Miji" or "Mongar" or "Mro" or "Naga" or "Nepami" or "Newar" or "Nicobar" or "Onge" or "Rai" or "Rang" or "Raute" or "Sajolang" or "Santhal" or "Sentinelese" or "Sindhis" or "Sulemani" or "Sunuwar" or "Tamang" or "Thakali" or "Thangmi" or "Tharu" or "Tripura" or "Tumbahangphe" or "Wanniyala-Aetto" or "Yakkha" or "Yolmopa").ti,ab,kw. [Indigenous, Indian Subcontinent] | 963 |
| 50 | (Aguacatec or Akwa'ala or Abxubal or Ayuukja'ay or "Batzil k'op" or Binizaa or "Chichimeca Jonaz" or Chinantec or Chocho or "Ch ol" or Chontal or "Chuj" or Cochimi or Comcaac or Hamasipini or Harijio or "Ha shuta enima" or "Hach t'an" or Huastecor Hnahnu or Hnatho or "indos mexicano" or Ixcatec or Ixil or Jacaltec or K'akchikel or K'anjobal or Kanjobal or Kaqchikel or Kechi or K'iche or Kikapooa or Kikapu or Kiliwa or "Ko'lew" or "K'op o winik atel" or Kumiai or Lacandon or Laymon or Makurawe or Maya or Maya'wiinik or Mazahua or Mazatec or Me'phaa or Mesoamerindian* or Mexicanero or Mexikatlajtolli or "Mixe" or Mixtec or Motocintleco or Mti'pa or Nahuas or Ocuiltec or Otomi or (Oaxaca not Oaxaca-Blinder) or "Pame" or Papago or Tlahuica or Paipai or "Pima Bajo" or "pueblos indigenas" or Purepecha or P'urhepecha or Qatok or (Quiche not Guatemala) or Q'iche or Raramuri or "Runixa ngiigua" or ("Seri" not "Seri 82") or "Slijuala sihanuk" or Tacuate or Tarahumara or Teenek or Tepehua or Ti'pai or Tlapanec or "Tohono O'odham" or Totonac or Tachiwin or "Tsa jujmi" or Tzotzil or "Tu'un savi" or Tzeltal or "Uza" or "Winik" or Xigue or Yucatec or Zapotec).ti,ab,kw. [Indigenous, Mexico] | 118 |
| 51 | ("Bedouin*" or "Jahalin" or "al-Kaabneh" or "al-Azazmeh" or "al-Ramadin" or "al-Rshaida").ti,ab,kw. | 19 |
| 52 | ((nomadic or seminomad* or "semi nomad*") adj3 ("people" or "peoples" or "elder" or "elders" or "grandmother*" or "grandfather*" or "parent*" or "women" or "men" or "woman" or "man" or "child*" or "youth" or "youths" or "baby" or "babies" or "tribe" or "tribes" or "tribal" or "shaman*" or "native" or "patient*")).ti,ab,kw. | 8 |
| 53 | or/51-52 [Indigenous, Middle East] | 27 |
| 54 | (maori* or maaori* or "m aori" or "ma ori" or moriori or tangata or whanau or rangatahi or tamariki or wahine or marae or hauora or kaupapa or tinana or hinengaro or wairua).ti,ab,kw. [Indigenous, New Zeland] | 414 |
| 55 | (Saami or Sampi or (Sami not Ulus) or Samis or Southernsami* or Umesami* or Pitesami* or Lulesami* or Northernsami* or Enaresami* or Kolasami* or Lapp or Lapps or Lappish or Lappland or (Lapland* not longspur) or Lappalainen* or Saamelainen* or reindeer herd* or reindeer culture* or reindeer pastoral* or Lappbys or Samebys or reinbeitesdistrikt or paliskunta or siida).ti,ab,kw. [Indigenous, Northern Europe] | 77 |
| 56 | (Ainus or Ainu or Aleuts or Alyutors or Chukchis or Chuvans or Dolgans or Enets or Entsy or Yupik or Yup'ik or Yuit or Yupigyt or Chaplino or Naukan or Itelmens or Kamchadals or Kereks or "Komi" or Koryaks or Nenets or Nentsy or Nganasans or Tavgi or Sami or Veps or Yukaghirs or Chulyms or Evenks or Tungus or Evens or "Kets" or Khantys or Mansi or Vguls or Selkups or Teleuts or Nanais or Nanaitsy or Negidal or Nivikh or Oroch or orok or Taz or udege or ulch or Kumadins or Chelkans or Shorians or Soyots or Telengits or Tofalars or Tugalars or "Tufans" or "Todzhins" or Laks or Tabasarans or Turuls or Aguls or Tsakhurs or Kumyks or Nogais or "Andis" or Akhvakh or Archins or Bagvalals or Bezhta or Botlikhs or Chamalals or Godoberi or Hinukh or Hunzibs or Khwarshi or Karata or Tindis or Tsez or Abazin or Besermyan or Izhorians or Karelians or Nagaybaks or Setos or Shapsugs or Quratay).ti,ab,kw. [Indigenous, Russia] | 506 |
| 57 | (Abipon or Achuar or Achuagua or Akawaio or Amarizana or Andoque or Akawaio or Akuriyo or Anauya or Araona or Arawak or Ayamn or Aguaruna or Amahuaca or Amarakaeri or Andoa or Arabela or Arawak or Arhuaco or Ashaninca or Asheninca or Atsahuaca or Aymara or Ayoreo or Bakairi or "Baniva" or Barasana or Baniwa or "Baure" or Bororo or Cabiyari or Cacataibo or Caquinte or Cacua or Cahuarano or "Caiua" or "Camara Indians" or Camaracoto or Camsa or Canamari or Candoshi or Canela or Canichana or Capanahua or Carapana or Cariay or "Carib" or Carijona or Carutana or Cashibo or Cashinahua or Cawishana or Cavinena or Caxuiana or Cayuvava or Chontaquiro or Cocama or "Cubeo" or Curipaco or Chacobo or Chaima or (Chana not striatus) or Chapacura or Charrua or Chimila or Chitonahua or Chorote or Chipaya or Chiquitano or Chulupi or Carare or Coconuco or Cofan or Coreguaje or Coyaima or Chamacoco or Chamicuro or Chayahuita or Cocama or "Culina" or Culino or Cubeo or Cuiba or "Cuiva" or Cumanagoto or Curripaco or "Deni" or Desano or Embera or Guarani or Guajajara or "Guana" or Guanano or Guarayo or Guarayu or Guahibo or Guajiro or Guambiano or Guanano or Guayabero or Guarequena or Guinao or "Guana" or Gayon or Guahibo or Hixkaryana or Huachipairi or Huambisa or Huarayo or Lauanaua or Ikpeng or Ingariko or Irantxe or Itonama or Inapari or Iquito or Isconahua or Jumana or Japreria or Jirajara or Juruti or Jaqaru or Jebero or Kadiweu or Kaingang or Kamayura or Karaja or Karipuna or "Kariri" or Katukina or Kaxarari or Kayabi or Kayapo or "Kuikuro alapalo" or Kulina or "Kaiwa" or Kallawaya or "Kogui" or "Kuna" or Kaweskar or "Lule" or Macuna or Maipure or Mapuche or Mataco or Mocovi or Machinere or Machinerev or Machiguenga or Macushi or Macuna or "Madi" or Malayo or Mamainde or Manao or Mandauaca or Mandawaka or Mapidian or Mapuche or Mapidian or Maquiritare or Maquiritari or Maragua or Marawan or Mariate or Marubo or Mastanahua or Mataco or Matipuhy or "Matis" or "Matses" or Mawakua or Mawakwa or Maxakali or Mehinaku or Miranha or Moronawa or Munduruku or Movima or Muellama or Muinane or Mapoyo or "Mashco Piro" or Muniche or Nambikwara or Nocaman or Nuquini or Nomatsiguenga or Nanti or Ocaina or Omagua or Orejon or "Opon" or Pacahuara or "Paez" or Paicone or Palicur or Panare or "Pano" or "Paresi" or Paumari or "Pemon" or Pilaga or Puelche or Pauna or Pauserna or Piapoco or Piraha or Piratapuyo or Pisabo or Piaroa or "Pijao" or Piratapuyo or Paraujano or Pemon or Pemono or Piapoco or Puinave or Patamona or Poyanawa or Puinave or Puquina or Quechua or Quichua or Retuara or Resigaro or Reyesano or Sabanes or "Saliba" or Saluma or Sarave or Secoya or Selknam or "Sensi" or Shaninawa or Shapra or Sharanahua or Shebayo or Shiwiar or Shikiana or Sikiana or Siriono or Sinsiga or "Siona" or Suruwaha or Tacano or Tamanaco or Tiahuanaco or Tariano or Tehuelche or Tariano or Tatuyo or "Tembe" or "Terena" or Telembi or Ticuna or Ticuna or Tiriyo or Tiwanaku or Tiwanaku or "Torom" or "Totoro" or Tsimane or Tuberao or "Tucano" or Tunebo or Tuxinawa or Tuyuca or Uainuma or Urarina or Vilela or Waimaha or Waiampi or Waiwai or Wapishana or Waraiku or Warekena or Waura or Wayampi or Wayana or Wirina or Waimaha or Waunana or "Wiwa" or "Warao" or Wayuu or Witoto or Xavante or Xipaya or Xiriana or Xokleng or Yabaana or Yaminawa or Yaminahua or Yaruma or Yawalapiti or Yuracare or Yabarana or Yavitero or "Yine" or Yamana or Yaghan or Yucuna or Yurumangui or Yukpa or Yanesha or Yoranahua or Yagua or Yaminahua or Zaparo or Zamuco or "Trio Indians" or "More Indians" or "Bare Indians").ti,ab,kw. | 124 |
| 58 | (("Inga" or "Maca" or "Leco" or "Mojo" or "Uro" or "Maco" or Lengua or "Toba" or "Zoe" or "Ona" or "Catio" or "Passe" or "Bari" or "Awa" or "Bora" or "Bara" or "Remo" or "Pano" or "Sape") adj3 (Indians or Indian or Indigenous or Amerindian* or Aborigin* or people or peoples or elder or elders or grandmother* or grandfather* or parent* or women or men or woman or man or child* or youth or youths or baby or babies or tribe or tribes or tribal or shaman* or native or patient*)).ti,ab,kw. | 195 |
| 59 | or/57-58 [Indigenous, South America] | 317 |
| 60 | or/1-59 [Indigenous, Global] | 28148 |
| 61 | smoking cessation/ or smoking reduction/ or "tobacco use cessation"/ | 5886 |
| 62 | exp "Tobacco Use Cessation Devices"/ | 857 |
| 63 | exp Smoking Cessation Agents/ | 4773 |
| 64 | Vaping/ | 229 |
| 65 | ((smok* or tobacco* or cigar* or Vape? or vaping or e-cigarette? or e-liquid? or e-cig? or ecig? or eliquid? or ecigarette? or juul or nicotine) adj5 (quit* or ceas* or cessation* or stop* or suspend* or desist* or end* or break* or cutoff* or reduct* or reduce? or reducing or termin* or discontinu* or abstin* or dehabituat* or de-habituat*)).ti,ab,kw. | 17185 |
| 66 | (pharmaco* adj2 (smok* or tobacco* or cigar* or Vape? or vaping or e-cigarette? or e-liquid? or e-cig? or ecig? or eliquid? or ecigarette? or juul or nicotine)).ti,ab,kw. | 554 |
| 67 | (Nicorette* or bupropion* or vareniclin* or nortriptylin* or clonidin* or chantix* or champix* or wellbutrin* or well-butrin* or Zyntabac* or Quomen* or Zyban* or Amfebutamon* or Cytisine* or baptitoxin* or cytisiniclin* or Polacrilex* or sophorin*).ti,ab,kw. | 8280 |
| 68 | (nicotine adj2 (gum* or lozenge* or patch* or spray* or tablet* or sublingual* or sub-lingual* or inhal* or strip? or microtab* or replace*)).ti,ab,kw. | 3745 |
| 69 | ((smok* or tobacco* or cigar* or Vape? or vaping or e-cigarette? or e-liquid? or e-cig? or ecig? or eliquid? or ecigarette? or juul or nicotine) adj2 (intervention* or treatment* or program* or therap*)).ti,ab,kw. | 9351 |
| 70 | or/61-68 | 25447 |
| 71 | 60 and 70 | 481 |
| 72 | limit 71 to yr="2024 -Current" | 45 |

## S4. APA PsycINFO Search

| Database: APA PsycInfo 1806 to May 2025 Week 3  Platform: Ovid  Date Searched: May 28, 2025 | | |
| --- | --- | --- |
| **#** | **Searches** | **Results** |
| 1 | exp indigenous populations/ | 18871 |
| 2 | tribes/ | 1464 |
| 3 | shamanism/ | 633 |
| 4 | ((traditional adj1 (medicine* or heal* or food* or health*)) not (Chinese or China)).ti,ab,id. | 2931 |
| 5 | (Aboriginal? or aborigine? or aborigen* or amerindian* or amerindio* or indian? or indigene* or indigenous* or indigena* or nativo or originarios or tribe? or tribal*).ti,ab,id. | 51366 |
| 6 | or/1-5 [Indigenous, Broad] | 59104 |
| 7 | (Aamjiwnaang or Abenaki? or Abitibiwinni or Ahtahkakoop or Aishihik or Aklavik or Akuliarmiut* or Akwesasne* or Alert Bay or Aleut* or Alexandra Fiord or Algonqui?n* or Amadjuak or Amerind* or Amisk or Anish?na?b?e* or Aquiatulavik Point or Arctic or Armstrong settlement or Aroland or Arviat or Asimakaniseekan or Assabaska or Assiniboine or Athabasca? or Athapaskan or Atikamek* or Attawapiskat or Aundeck-Omni-Kaning or autochtone*).ti,ab,id. | 1734 |
| 8 | (Baffin Island or Baker Lake or Barriere Lake or Batchewana or Bathurst Inlet or Beardy or Bearfoot Onondaga or Beaver Creek or Behochoko or Belcher Islands or Beothuk* or Berens River Bloodvein or Betsiamite* or Bibigo?ining or Biinjitiwabik Zaaging or Bimose or Bingwi Neyaashi or Birch Portage or Bittern Lake or Bkejwanon* or Blackfoot or Bois-brule* or Brochet or Budd's Point or Buffalo River or Bungee or Bunibonibee or Bylot Island).ti,ab,id. | 76 |
| 9 | (Cacouna or Cambridge Bay or Canoe Lake or Canupawakpa or (Cape adj1 (Dorset or Dyer or Smith)) or Carcross or Carmacks or Carrot River or Carry the Kettle or Cayuga or Charlton Depot or Chesterfield Inlet or Chipewyan or Chipp#wa* or Chisasibi or Clyde River or Coal Harbour or Colville Lake or Coral Harbour or Coucoucache or country food* or Craig Harbour or Cree or Cypress Health).ti,ab,id. | 666 |
| 10 | (Dakelh or Dakota or Dauphin River or Dawson City or Day Star or Deh Cho or Deline or Dene or Denesuline or Destruction Bay or Devon Island or Dipper Rapids or Dogrib* or dokis or Dopitciwa* or Dundas Harbour or Dunne?za).ti,ab,id. | 1286 |
| 11 | (Eabametoong or (Eagle adj (Plains or village)) or Eastmain or Eeyou Istchee or Ehdiitat or Ekuanitshit* or Elak Dase or Ellesmere Island or Ennadai or eskimo* or Esquimau* or Essipit* or ethnomedicin* or ethnopharmacol*).ti,ab,id. | 525 |
| 12 | ((first adj2 (nation? or people?)) or First-Nation? or Firstnation? or Fisher River or Fond du Lac or (Fort adj1 (Conger or Good Hope or Hope or Liard or McPherson or Providence or Ross or Selkirk or Simpson or Smith)) or Four Portages).ti,ab,id. | 2802 |
| 13 | (Grandmother's Bay or Gespeg or Gesgapegiag* or Ginoogaming or Gits#an or Gjoa Haven or (God's adj1 (river or lake)) or Grise Fiord or Gwi?ch?in or Gwichya).ti,ab,id. | 10 |
| 14 | (Haida or Haines Junction or Haisla or Hall Beach or (Hare adj1 (boy* or girl* or men or man or woman or women or people* or person or persons or band? or native* or tribe or tribal)) or Hatchet Lake or Haudenosaunee or Hay River or Hazen Camp or Heiltsuk or Huron or Huron-Wendat or Huronne-Wendat).ti,ab,id. | 114 |
| 15 | (Han not (Chinese or China)).ti,ab,id. | 3920 |
| 16 | (Igloolik or Igluligaarjuk or Ikaluit or Ils a la Crosse or ((indian or council) adj3 band) or Innu? or Inuit* or Inuk* or Inupiat* or Inuvik or Inuvialu* or Inuinnaqtun or Iqaluit or Iqaluktuttiaq or Iroquois or Isachsen or Island Lake or Itivimiut* or Ivujivik).ti,ab,id. | 706 |
| 17 | (James Bay or Joseph Bighead).ti,ab,id. | 26 |
| 18 | (Kabapikotawangag or Kalaallit* or Kahkewistahaw or Kahnawa?ke or Kanesatake or Kasabonika Lake or Kashechewan or Kaska? or Kawacatoose or Kawawachikamach* or Kebaowek or Keeseekoowenin or Kekerten or Keno City or Keewaytinook or Kee?Way?Win or Kelsey Trail Health or Kiashke Zaaging or Kimmirut or Kingnaitmiut* or King William Island or Kinistin or Kinonjeoshtegon or Kipawa or Kipisa or Kitchenuhmaykoosib or Kitcisakik or Kitigan Zibi or Kitikmeot or Kitimat or Kitsakie or Kivalliq or Kivitoo or Konadaha Seneca or Koocheching or Ktunaxa or Kugaaruk or Kugluktuk or Kutchin* or Kuujjuaq or Kwakiutl or Kwakwaka?wakw).ti,ab,id. | 92 |
| 19 | (La Plonge or (Lac adj (Brochet or Romanie or John or Simon or La Hache or La Ronge)) or Lake Manitoba or Lean Man or Lenape or (Little adj (Black Bear or Saskatchewan or Red River or Salmon)) or Listuguj* or Long Point First or Lucky Man).ti,ab,id. | 22 |
| 20 | (M?Chigeeng or MacDowell Lake or Mackenzie River Basin or MacKenzie Valley or Magnetawan or Maguse River or Makaw or Makaoo or Malecite* or Maliotenam or Maliseet or Mamawetan or Mamit Innuat or Mamuitun or Manawan or Mani-Utenam or Manitoulin or Manto Sipi or Mashteuiatsh or Matachewan or Mathias Colomb or Matimekosh* or Matimekush* or Mattagami or Mawiomi or (medicine adj (man or men or woman or women)) or Metchif or Metif or metis or Miawpukek or Michif or Mic?mac or mic mac or Migmaw or Mig maw or Mi?gmaq or Mi?gmawei or Mi?kmaq or Mi#chif or Mingan or Ministikwan or Minoahchak or Mirond Lake or Mishkeegogamang or Missanabie or Mistawasis or Mistissini* or Mitaanjigaming or Mixed-blood* or Mo?hawk or Mocreebec or Montagnais or Moos-Toosis or (moravian adj2 thames) or Moraviantown or Morin Lake or Mosquito-Grizzley Bear* or Muscowpetung or Mushkegowuk or Muskoday or Muskowekwan).ti,ab,id. | 672 |
| 21 | ("Nacho Nyak Dun" or Naicatchewenin or Nain or Nako#a or Namaygoosisagagun or Nan#sivik or Naongashiing or Naotkamegwanning or Naskapi* or Natashquan* or (native adj2 (group? or health or community or communities or person$ or people$ or population* or america* or canad* or Nation or band or bands or reserv*)) or (Native* adj1 (man or men or women or woman or boy* or girl* or child* or adolescent* or youth? or adult*)) or Nekaneet or Nelson House or Nemaska* or Nemiscau or Neskantaga or Netsilik or New Thunderchild or Nibinamik or Nigigoonsiminikaaning or Nihtat or Nipissing or Nisga?a or Nisichawayasihk or Nlaka?pamux or Norman Wells or Northlands Nursing Station or North Slave or (northern adj1 (Saskatchewan or Manitoba or Quebec or Alberta or British Columbia or Ontario)) or Norway House or Nottingham Island or Nugumiut* or Nunatsiavut or Nunav* or Nutaqqavut or Nuwata or Nuxalk or Nuu-chah-nulth).ti,ab,id. | 10285 |
| 22 | (ob?djiwan or Obashkaandagaang or Ocean Man or Ochapowace or O-Chi-Chak-Ko-Sipi or Ochiichagwe or Odanak or off-reserve or Oji-Cree or Ojibw* or Okanagan or Okanese or Okemasis or Oki or Old Crow or One Arrow or Oneida or Onion Lake or Onigaming or Onkwehonwe or Onodaga or on-reserve or Opaskwayak or Opawakoscikan or O-Pipon-Na-Piwin or Opitciwa* or Oqomiut* or Original people? or Ouj?-Bougoumou or Ouje?Bougoumou or Oweenkeno).ti,ab,id. | 397 |
| 23 | (Padlei or Padloping Island or Pakua Ship* or Pakuaship* or Pangnirtung or Parmachene or pasqua or Passamaquoddy or Pauingassi or Pauktuutit or Paulatuk or Peepeekisis or Peguis or Pelly or Perry Island or Pessamit* or Peter Ballantyne or (Peuple adj2 (premier or racine or natif*)) or Pheasant Rump or Piapot or Pikogan or Pikwakanagan or Pinaymootang or Pine Bluff or Pond Inlet or Port Burwell or Potato River or Potawatomi or Prairie North Health or premiere nation or Prince Albert Parkland Health or Pukatawagan).ti,ab,id. | 47 |
| 24 | (Qaumauangmiut* or Qayuqtuvik or Qikiqtarjuaq or Qikqta* or Qingaumiut* or Qoloqtaaluk).ti,ab,id. | 2 |
| 25 | (Rankin Inlet or Rapid Lake or Read Island or Red Pheasant or red road or Red Sucker Lake or Repulse Bay or residential school* or Resolute Bay or Resolution Island or Roseau River).ti,ab,id. | 1010 |
| 26 | (Sabaskong or Sachs Harbour or Sagamok or Sagkeeng or Sahtu or Sakimay or Salish or Sa?lteaux or Sandy Narrows or Sanikiluak or Sanikiluarmiut* or Sapotaweyak or Saulteaux or Saumingmiut* or Sayisi Cree or Schefferville or Sec wepmc or Seekaskootch or Sekani or Sekon or Seneca or Sept-Iles or shaman* or Shamattawa or Shawanaga or Sheguiandah or Shesheep or Sheshegwaning or Shoal Lake or Shubenacadie or Sikusilaamiut* or Sioux or Siphik or Six Nations or Skownan or Slave Lake or Slavey* or Southend or South Indian Lake or South Slave or Sunrise Health or Standing Buffalo or Stanjikoming or Stanley or Starblanket or Stl?atlimc or St Theresa Point or Sturgeon or Subarctic or Sucker River or Sweetgrass).ti,ab,id. | 3990 |
| 27 | (Tadoule Lake or Tagish or Tahltan or Takuaikan or Talirpingmiut* or Talo?yoak or Ta?an Kwach?an or Tanana? or Tanquary Camp or Tarramiut* or Tasttine or Tataskweyak or Tavani or Taykwa Tagamou or Temagami or T#mis#aming or Teslin or Tetlitn or Thom Bay or Tlingit or Tlicho or Tli Cho or Tootinaowaziibeeng or Tr?ondek Hwech?in or treaty or treaties or Trout Lake or Tsilgehtchic or Tsilhqot?in or Tsimshian or Tsuu T?inia or Tulita or Tuktoyaktuk or Tumor Lake or Tungasugit or Turtle island or Tuscarora or Tutchone* or Tuttarvingat).ti,ab,id. | 1151 |
| 28 | (Uashat or Ulukhaktck or Umingmaktok or Unamen Shipu or Ungava or Upper Liard or Uqqurmiut* or (urban adj3 (Indian* or Native* or Aboriginal* or indigenous*))).ti,ab,id. | 1020 |
| 29 | (Victoria Island or Vuntut).ti,ab,id. | 1 |
| 30 | (Waban-Aki or Wabaseemoog or Wabauskang or Wabigoon Lake or Wager Bay or Wahgoshig or Wahnapitae or Wahpeton or Wakashan or Wapachewanak or Wapekeka or Wa-Pii or Wasagamack or Wasauksing or Washagamis Bay or Waskaganish or Waswanipi or Waterhen or Watson Lake or Wauzhushk or Wawakepewin or Waywayseecappo or Webequie or Weenusk or Wemindji or Wemotaci or Wendake or Wendat* or Wet?suwet?en or Whale Cove or Whapmagoostui or (White adj (Bear or Cap)) or Whitehorse or Wikwemikong or Willow Bunch or Winneway or Witchekan or Wolastoqiyik or Wolinak or Wood Mountain or Woody Lake or Wrigley or Wuskwi or Wyandot).ti,ab,id. | 249 |
| 31 | (Yellowkni* or Yellowquill or Yellow Quill or York Landing or Yukon).ti,ab,id. | 90 |
| 32 | or/7-31 [Indigenous, Canada] | 28088 |
| 33 | ("A' ani" or Absaroka or Haaninin or Atsina or "Gros Ventre" or Acopsel or Tlacopsel or Lacopsel or Ahtna or Ahtena or Akenitsi or Occaneechi or Akokisa or Horcoquisa or Orcoquizas or Aleut or Unangax or Unangan or Alibamu or "Alabama Alsea" or Alutiiq or Sugpiag or Amahami or Awaxawi or Androscoggin or Arosaguntacook or Ameriscoggin or Anishinaabeg or Chippewa or Anihsinape or Saulteaux or Apalachee or Aranama or "Texan Coahuilteca" or Tamique or Arikara or Sahnish or Arickaree or Adakadaho or Assiniboine or Nakota or Nakoda or Nakona or "Atsa' Kudok-wa" or Awatixa or Bannock or "Snake Indian*" or Bidai or Quasmigdo or Biloxi or Blackfoot or Niitsitapi or Sikasikaitsitapi or Cahto or Kaipomo or Cahuilla or Ivilyuqaletem or Ivilyuat or Catawba or Inna or Iswa or Chemehuevi or Chickasaw or "Chilula Chimakum" or Aqokulo or Chimariko or Chiricahua or Tsokanende or Chitimacha or Chetimachan or Sitimacha or Chowanoke or Roanoke or Chumash or Ciboney or "Taino Ciwat" or Clatsop or Coos or Coosa or Uchis or Chiaha or Coste or Talisi or Coquille or Kokwell or Coso or Cowlitz or Taitnapam or "Crow Nation" or "Cui Ui Ticutta" or Cupeno or Kuupangaxwichem or Cupa or "Cup' ig" or Nunivak or "Dakota Oyate" or Lakota or Nakota or Santee or Teton or Sioux or Deadose or "Deg Xina" or "Deg Xit' an" or Kaiyuhkhotana or "Deg Hit' an" or "Dena' ina" or Tanaina or "Dichinanek' Hwt' ana" or "Upper Kuskokwim Athabascan*" or Kolchan or Goltsan or "Tundra Kolosh" or "Do lkabya" or Duwamish or Esselen or Eyak or "Gidi' tikadi" or Guwevkabaya or "Gwich' in" or Kutchin or Haida or Xaadas or Xaat or Halchidhoma or Havasupai or "Green Water People" or Hiratsa or Hiraaca or "Ho-chaaqa" or Winnebago or Holikachuk or Innoko or "Tlegon-khotana" or Hopi or "Houma-Louisiana" or Huaco or Waco or Hualapai or Hupa or Natinixwe or "Natinook-wa" or "Hwech' in" or Hankutchin or "Iroquois Confederacy" or "Hodinoso ni" or "Illinois Confedera*" or Ilinoweg or Illini or Inupiat or Inuit or Ioway or Baxoje or Jicarilla or Juaneno or Acjachemen or Jumano or Kalapuya or Clackama or Kalispel or "Pend d' Oreilles" or Qlispe or Karuk or Karok or "Chum-ne" or Katkoc or Kansa or Kanza or Kawaiisu or Nuwa or Kennebec or "Kinipekw Kittitas" or Klickitat or "Qwu' lh-hwai-pum" or "Awi-adshi" or Mahane or Wahnookt or "Koa' aga' itoka" or Keresan or Kichai or Kitsai or Keechi or "K' itaish" or Kiowa or Gaigwu or Cauigu or Kutjau or "Kwu-da" or "Tep-da" or Kitanemuk or Kittitas or Klickitat or "Qwu' lh-hwai-pum" or "Awi-adshi" or Mahane or Wahnookt or "Koa' aga' itoka" or Konkow or "Koop Ticutta" or Koyukon or Ktunaxa or Kootenai or Kucadikadi or "Kotsa' va" or Kumeyaay or "Tipai-Ipai" or Kamia or Diegueno or Kwapa or Cocopah or Cucapa or "Xawitt kwnchawaay" or Lassik or Lenape or "Leni-Lenape" or Lipan or Luiseno or Payomkawichum or Madqwadabaya or "Desert Yavapai" or Mahican or Mohicans or Makah or Makuhadokado or Maliseet or Wolistoqiag or Manahoac or Mahock or Meipontsky or Mandan or Mattole or "Bear River" or "Tul' bush" or "Ni' ekeni" or Meherrin or Menominee or Mackinac or Mescalero or Myaamiaki or Kickapoo or Twigtwee or Missouria or Miwok or Miwuk or Moadokado or Modoc or Mohave or "Aha Makhav" or Mohawk or "Kaneng' hega" or Molala or Molale or Molele or Nyyhmy or Moosonee or "Moose Cree" or Monsonis or Multnomah or (Chinook not cultivar) or Nabedache or Nabaydacu or Wawadishe or Nabiltse or Dakubetede or "Nacho Nyak Dun" or Tutchone or Nacono or "Na' isha" or Nanticoke or Navajo or Ndee or Nial or Niimiipu or "Nez Perce" or Watapala or Watapahlute or Nisenan or Nisqually or Nomlaki or Noamlakee or "Central Wintun" or Nongatl or Nottoway or Cheroenhaka or "Northern Cheyenne" or Ohlone or Costanoan or Omaha or "O' odham" or Pima or Papago or Osage or Otoe or Otse or "Ozav Dika" or Palus or Passamaquoddy or Pestomuhkati or Patiri or Petaros or Pastia or Patwin or "Southern Wintun" or Panis or Skidi or Pedee or Penobscot or "Petun Piipaash" or "Kokmalik' op" or Piscatawa or Doeg or Conoy or "Pit River" or Pomo or Kashaya or Ponca or Ponka or Pottawatomi or Bodewadmik or Powhatan or Puyallup or Spuyalepabs or Quapaw or Ugahxpa or Quechan or Kwtsaan or Quileute or Salinan or Saponi or Monacan or Sapon or "Eastern Blackfoot" or Christanna or Sawawatodo or Serrano or Taaqtam or "Maarenga' yam" or Yuhaviatam or Shasta or Chasta or Sasti or Shoshone or Siletz or Sinkine or Sinkyone or "Siuslaw Umpqua" or Skitswish or "Schitsu' umash" or Snohomish or Snuqualmi or Sokoki or Missiquoi or Stillaguamish or Stoluckwamish or Suquamish or Sutaio or Swinomish or Skagit or Syilx or Sotaae or "Taga Ticutta" or Takelma or Dagelma or Taltushtuntede or Galice or "Tanan Gwich' in" or Taos or Taovaya or Tataviam or Alliklik or Tawakoni or Tahuacano or Tenino or Thawikila or Hathawekela or "Fort Ancient" or Tigua or Tillamook or Nehalem or Timbisha or Panamint or Timpanogos or Tlingit or "Toi Ticutta" or Tolowa or "Talawa Dini' " or Tongva or Gabrieleno or Fernandeno or Tobikhar or Tonkawa or Ticanwatic or Tsikip or Appalousa or Opelousa or Tsitsistas or Tubatulabal or Tukabatchee or Tuscarora or Tomahittan or Kuskarawock or Tutelo or Tutero or Totteroy or Tutera or Yusan or Tututni or Umatilla or Umpqua or Waccamaw or Waxmaw or Wadatika or "Harney Valley Paiute" or Wailiki or Waluulapam or "Walla Walla" or Walpapi or Huipui or Wampanoag or Massasoit or Wanapum or Wappo or Washoe or Wichita or Willapa or Kwalhioqua or "Wi pukba" or "Verde Valley Yavapai" or Wintu or "Northern Wintun" or Wiyot or "Wee' at" or Weyet or Yakama or "Yamosopo Tuviwarai" or Yaqui or Yoeme or Yatasi or Yattasih or "Yavbe' " or "Yavapai" or "Ysleta del Sur" or Yojuane or Yokuts or Mariposa or Yuki or Yupighyt or "Yup'ik" or Yupik or Yurok or "Olekwo'l" or Zuni or "Native Hawaiian*" or Kanaka or Maoli or Oiwi).ti,ab,id. | 5112 |
| 34 | ((Applegate or Alabama or Delaware or Flathead or Hohe or Iowa or Ishak or Kaw or Kato or Spokane or Miami or Arkansas or Tali or Tunica or Pawnee or Okanagan or "Coeur D' Alene" or Piscataway or Ree or Tula or Wichita or Yuma) adj3 (reservation* or nation or people or peoples or population or man or men or woman or women or child* or youth* or elder or elders or communit* or tribe or tribes or tribal or Indian*)).ti,ab,id. | 1068 |
| 35 | or/33-34 [Indigenous, United States] | 6152 |
| 36 | ("Abakuria" or "Abaluhya" or "Abagusii" or "Abakuria" or "Aembu" or "Agikuyu" or "Akamba" or "Anuak" or "Anywaa" or "Amazigh" or "Ambala" or "Ambeere" or "Ambundu" or "Ambuun" or "Amharan" or "Angba" or "Baaka" or "Baamba" or "Babindi" or "Babini" or "Baboma" or "Bachokwe" or "Bacwa" or "Bafumbira" or "Baganda" or "Bagyele" or "Bagwere" or "Bagyeli" or "Bakiga" or "Bakola" or "Baholo" or "Bakalanga" or "Bakiga" or "Bakolo" or "Bakongo" or "Bakonjo" or "Baluba" or "Balunda" or "Balovale" or "Bamasaba" or "Bambuti" or "Bangala" or "Bangoli" or "Bangungu" or "Bantu" or "Banyankole" or "Banyarwanda" or "Banyole" or "Banyoro" or "Bapende" or "Bapedi" or "Barabaig" or "Barombi" or "Barundi" or "Baruuli" or "Basamia" or "Basoga" or "Batswana" or "Batooro" or "Batsamba" or "Batswana" or "Batwa" or "Bayaka" or "Bedzan" or "Bazombe" or "Bebayaka" or "Bedzan" or "Bhaca" or "Biaka" or "Borana" or "Chewa" or "Copts" or "Cormorian" or "Cushitic" or "Dahalo" or "Datooga" or "Dikidiki" or "Dogon" or "Ewondo" or "Fulani" or "Fuliru" or "Ganguela" or "Gciriku" or "Gyele" or "Hadza" or "Hadzabe" or "Haillom" or "Haratin" or "Herero" or "Himba" or "Hlubi" or "Iriryen" or "Iqvayliyen" or "Kabyle*" or "Kalenjin" or "Kanioka" or "Kanioka" or "Kaonde" or "Karamojong" or "Kavango" or "Kereuyu" or "Khoikhoi" or "KhoiSan" or "Kikuyu Kwangali" or "Lokele" or "Lowme" or "Lotuko" or "Lwalwa" or "Maasai" or "Makonde" or "Makua" or "Mande" or "Masalit" or "Matumbi" or "Mayeuyi" or "Mayeyi" or "Mbenga" or "Mbukushu" or "Mbochi" or "Mboro" or "Mbuti" or "Medzan" or "Mijikenda" or "Mozabite*" or "Nafusa" or "Ndebele" or "Ngombe" or "Namaqua" or "Nyanga" or "Nyamwezi" or "Ogiek" or "Ovambo" or "Ovimbundu" or "Phuthi" or "Pokomo" or "Rendille" or "Riffian" or "Riffians" or "Sakuma" or "Samburu" or "Sandawe" or "Sangha" or "Sango" or "Sengwer" or "Serer" or "Sesotho" or "Shangaan" or "Shawiya" or "Shenwa" or "Shi" or "Shilluk" or "Sukua" or "Sukus" or "Swahili" or "Tabwa" or "Tambuka" or "Taveta" or "Thembu" or "Tigrayan" or "Topoke" or "Tsonga" or "Toubou" or "Tuareg" or "Tumbuka" or "Ugana" or "Wochua" or "Xhosa" or "Xindonga" or "Yoruba" or "Zenati" or "Zuwara" or (("Indigenous" or "Afar" or "Afars" or "Aka" or "Akie" or "Ameru" or "Asua" or "Ateker" or "Atwot" or "Awjila" or "Bafia" or "Baka" or "Bakongo" or "Bakwe" or "Balunda" or "Balovale" or "Bango" or "Bassa" or "Beja" or "Bekpak" or "Bemba" or "Bembe" or "Benet" or "Berber" or "Berbers" or "Bira" or "Bowe" or "Bubi" or "Budja" or "Bulu" or "Bunrun" or "Chaga" or "Chopi" or "Damara" or "Dinka" or "Djerba" or "Duala" or "Dzing" or "Efe" or "Elmolo" or "Fang" or "Foora" or "Fula" or "Fur" or "Ghomara" or "Ghadames" or "Gllana" or "Glu" or "Gogo" or "Gongo" or "Haya" or "Havu" or "Hema" or "Hima" or "Hunde" or "Hutu" or "Huva" or "Iboko" or "Igbo" or "Ijo" or "Jieng" or "Kadu" or "Kande" or "Kango" or "Katla" or "Kgaga" or "Khoe" or "Kola" or "Komo" or "Kota" or "Kua" or "Kuba" or "Kwango" or "Kx'z" or "Kxoe" or "Lala" or "Lozi" or "Luo" or "Luba" or "Lupu" or "Masmuda" or "Matmata" or "Mbala" or "Mbam" or "Mbo" or "Mbolo" or "Mbuza" or "Mongo" or "Mpondo" or "Myene" or "Naadh" or "Nama" or "Nande" or "Naro" or "Ngoni" or "Ndau" or "Ndebele" or "Ngoli" or "Ngondi" or "Ngoni" or "Nguni" or "Nkoya" or "Nkumu" or "Nuba" or "Nubian" or "Nuer" or "Nzebi" or "Ogoni" or "Omoro" or "Oroko" or "Pygmy" or "Popoi" or "Poto" or "Puru" or "Rashad" or (San not ("San Francisco" or "San Diego" or "San Antonio")) or "Sango" or "Sanhaja" or "Sena" or "Shilha" or "Shira" or "Shona" or "Shua" or "Sokna" or "Somali*" or "Sotho" or "Sua" or "Subu" or "Swazi" or "Taitaa" or "Tchokwe" or "Teke" or "Tembo" or "Tetela" or (Tonga and Africa*) or "Tshwa" or "Tsoa" or "Twa" or "Turkana" or "Tuu" or "Venda" or "Vira" or "Watta" or "Wakuti" or "Yaaku" or "Yaka" or "Yakoma" or "Yanzi" or "Yao" or "Yeke" or "Yela" or "Yeyi" or "Zulu") adj3 (population* or people or peoples or person or persons or elder or elders or man or men or woman or women or child* or youth* or clan or clans or tribe or tribes or tribal or family or families or parent* or grandparent* or elder or elders or grandmother* or grandfather* or baby or babies or infant or infants or patient or patients or speakers or speaking or village* or communit*))).ti,ab,id. [Indigenous, Africa] | 12223 |
| 37 | "torres strait islander*".ti,ab,id. [Indigenous, Australia] | 886 |
| 38 | (Acatec or Aguacateco or Amuzgo or Bokota or Boruca or Bribri or "Bri Bri" or Buglere or Cabecar or Cakchiquel or Changuena or Chatino or Chiapanec or Chicomuceltec or Chinantee or Chocho or Cholti or "Ch'olti'" or "Ch'olti'anor Chontal" or Chorotega or Chorti or Chuj or Chumbia or Corobici or (Cueva not Spain) or Cuicatec or Cuitlatee or Cuytec or Dorasque or Embera or Garifuna or Guatuso or Guaymi or Guaymis or Guetar or Huastec or Huave or Huetar or Itzaj or Ixil or Jacalteco or Jonaz or Kanjobal or Kekchi or Kuna or Maleku or Mangue or Matambu or Matlatzinca or Mazahua or Motozintlec or Mayan or Mayangna or Mestizo or Miskito or Mixtec or Mopan or Nahua or Nahuatl or Ngabe or Otomi or Pantec or Paya or Popoloca or Popoloc or Poqomam or Poqomchi or "Q'eqchi'" or Quiche or Quitirrisi or Sacapulteco or Sipacapense or Subtiaba or Tacaneco or Tarasco or Tamaulipec or Tamazultec or Tecoxquin or Tectiteco or Tecual or Tecuexe or Tepehura or Tepuztecor or Teribe or Terraba or Totonac or Trique or Tzeltal or Tzotzil or Tzutujil or Ulwa or Uspantec* or Uspanteko or Voto or Xinca or Waunana or Wounaan or Yucatec or Zapotec or Zoque).ti,ab,id. [Indigenous, Central America] | 1158 |
| 39 | (((China or Chinese) and "ethnic minorit*") or "Achang" or "Bonan" or "Bouyei" or "Blang" or "Deang" or "Dongxiang" or "Dulong" or "Ewenki" or "Gaoshan" or "Gelao" or "Hezhe" or "Jingpo" or "Jinuo" or "Kazak" or "Kirgiz" or "Korean" or "Lahu" or "Luoba" or "Manchu" or "Maonan" or "Menba" or "Miao" or "Mongolian" or "Mulao" or "Naxi" or "Oroqen" or "Ozbek" or " Pumi" or "Qiang" or "Shui" or "Tajik" or "Tatar" or "Tibetan" or "Tujia" or "Uigur" or "Yugur" or "Zhuang" or (("Hui" or "Yi" or "Dong" or "Yao" or "Bai" or "Hani" or "Li" or "Dai" or "She" or "Lisu" or "Va" or "Sui" or "Tu" or "Xibe" or "Daur" or "Salar" or "Nu" or "Gin" or "Jino") adj3 (ethnic or minorit* or population* or people or peoples or person or persons or elder or elders or man or men or woman or women or child* or youth* or clan or clans or tribe or tribes or tribal or family or families or parent* or grandparent* or elder or elders or grandmother* or grandfather* or baby or babies or infant or infants or patient or patients or speakers or speaking or village* or communit*))).ti,ab,id. [Indigenous, China] | 26903 |
| 40 | (Greenland* or Kalaallit or Kalaallisut or Nuuk or Sisimiut or Ilulissat or Qaqortoq Aasiaat or Maniitsoq or Tasiilaq or Uummannaq or Narsaq or Paamiut or Nanortalik or Upernavik or Qasigiannguit or Tunumiit or Inughuit or Avanersuarmiut).ti,ab,id. [Greenland] | 273 |
| 41 | ("Adivasis" or "Adnamanese" or "Andaman" or "Baluch" or "Baluchis" or "Bodo" or "Boro" or "Boros" or "Bote" or "Brahuis" or "Chakmas" or "Chepang" or "Chhantyal" or "Damai" or "Dewan" or "Ghale" or "Gurkha" or "Gurung" or "Hayu" or "Hyolmo" or "Jarawa" or "Jirel" or "Jumma" or "Kalash" or "Khas" or "Kirati" or "Koinch" or "Kulung" or "Kusunda" or "Limbu" or "Lohorung" or "Magar" or "Makrani" or "Mangar" or "Marma" or "Miji" or "Mongar" or "Mro" or "Naga" or "Nepami" or "Newar" or "Nicobar" or "Onge" or "Rai" or "Rang" or "Raute" or "Sajolang" or "Santhal" or "Sentinelese" or "Sindhis" or "Sulemani" or "Sunuwar" or "Tamang" or "Thakali" or "Thangmi" or "Tharu" or "Tripura" or "Tumbahangphe" or "Wanniyala-Aetto" or "Yakkha" or "Yolmopa").ti,ab,id. [Indigenous, Indian Subcontinent] | 1002 |
| 42 | (Aguacatec or Akwa'ala or Abxubal or Ayuukja'ay or "Batzil k'op" or Binizaa or "Chichimeca Jonaz" or Chinantec or Chocho or "Ch ol" or Chontal or "Chuj" or Cochimi or Comcaac or Hamasipini or Harijio or "Ha shuta enima" or "Hach t'an" or Huastecor Hnahnu or Hnatho or "indos mexicano" or Ixcatec or Ixil or Jacaltec or K'akchikel or K'anjobal or Kanjobal or Kaqchikel or Kechi or K'iche or Kikapooa or Kikapu or Kiliwa or "Ko'lew" or "K'op o winik atel" or Kumiai or Lacandon or Laymon or Makurawe or Maya or Maya'wiinik or Mazahua or Mazatec or Me'phaa or Mesoamerindian* or Mexicanero or Mexikatlajtolli or "Mixe" or Mixtec or Motocintleco or Mti'pa or Nahuas or Ocuiltec or Otomi or (Oaxaca not Oaxaca-Blinder) or "Pame" or Papago or Tlahuica or Paipai or "Pima Bajo" or "pueblos indigenas" or Purepecha or P'urhepecha or Qatok or (Quiche not Guatemala) or Q'iche or Raramuri or "Runixa ngiigua" or ("Seri" not "Seri 82") or "Slijuala sihanuk" or Tacuate or Tarahumara or Teenek or Tepehua or Ti'pai or Tlapanec or "Tohono O'odham" or Totonac or Tachiwin or "Tsa jujmi" or Tzotzil or "Tu'un savi" or Tzeltal or "Uza" or "Winik" or Xigue or Yucatec or Zapotec).ti,ab,id. [Indigenous, Mexico] | 1521 |
| 43 | ("Bedouin*" or "Jahalin" or "al-Kaabneh" or "al-Azazmeh" or "al-Ramadin" or "al-Rshaida").ti,ab,id. | 470 |
| 44 | ((nomadic or seminomad* or "semi nomad*") adj3 ("people" or "peoples" or "elder" or "elders" or "grandmother*" or "grandfather*" or "parent*" or "women" or "men" or "woman" or "man" or "child*" or "youth" or "youths" or "baby" or "babies" or "tribe" or "tribes" or "tribal" or "shaman*" or "native" or "patient*")).ti,ab,id. | 90 |
| 45 | or/43-44 [Indigenous, Middle East] | 555 |
| 46 | (maori* or maaori* or "m aori" or "ma ori" or moriori or tangata or whanau or rangatahi or tamariki or wahine or marae or hauora or kaupapa or tinana or hinengaro or wairua).ti,ab,id. [Indigenous, New Zeland] | 1900 |
| 47 | (Saami or Sampi or (Sami not Ulus) or Samis or Southernsami* or Umesami* or Pitesami* or Lulesami* or Northernsami* or Enaresami* or Kolasami* or Lapp or Lapps or Lappish or Lappland or (Lapland* not longspur) or Lappalainen* or Saamelainen* or reindeer herd* or reindeer culture* or reindeer pastoral* or Lappbys or Samebys or reinbeitesdistrikt or paliskunta or siida).ti,ab,id. [Indigenous, Northern Europe] | 468 |
| 48 | (Ainus or Ainu or Aleuts or Alyutors or Chukchis or Chuvans or Dolgans or Enets or Entsy or Yupik or Yup'ik or Yuit or Yupigyt or Chaplino or Naukan or Itelmens or Kamchadals or Kereks or "Komi" or Koryaks or Nenets or Nentsy or Nganasans or Tavgi or Sami or Veps or Yukaghirs or Chulyms or Evenks or Tungus or Evens or "Kets" or Khantys or Mansi or Vguls or Selkups or Teleuts or Nanais or Nanaitsy or Negidal or Nivikh or Oroch or orok or Taz or udege or ulch or Kumadins or Chelkans or Shorians or Soyots or Telengits or Tofalars or Tugalars or "Tufans" or "Todzhins" or Laks or Tabasarans or Turuls or Aguls or Tsakhurs or Kumyks or Nogais or "Andis" or Akhvakh or Archins or Bagvalals or Bezhta or Botlikhs or Chamalals or Godoberi or Hinukh or Hunzibs or Khwarshi or Karata or Tindis or Tsez or Abazin or Besermyan or Izhorians or Karelians or Nagaybaks or Setos or Shapsugs or Quratay).ti,ab,id. [Indigenous, Russia] | 1610 |
| 49 | (Abipon or Achuar or Achuagua or Akawaio or Amarizana or Andoque or Akawaio or Akuriyo or Anauya or Araona or Arawak or Ayamn or Aguaruna or Amahuaca or Amarakaeri or Andoa or Arabela or Arawak or Arhuaco or Ashaninca or Asheninca or Atsahuaca or Aymara or Ayoreo or Bakairi or "Baniva" or Barasana or Baniwa or "Baure" or Bororo or Cabiyari or Cacataibo or Caquinte or Cacua or Cahuarano or "Caiua" or "Camara Indians" or Camaracoto or Camsa or Canamari or Candoshi or Canela or Canichana or Capanahua or Carapana or Cariay or "Carib" or Carijona or Carutana or Cashibo or Cashinahua or Cawishana or Cavinena or Caxuiana or Cayuvava or Chontaquiro or Cocama or "Cubeo" or Curipaco or Chacobo or Chaima or (Chana not striatus) or Chapacura or Charrua or Chimila or Chitonahua or Chorote or Chipaya or Chiquitano or Chulupi or Carare or Coconuco or Cofan or Coreguaje or Coyaima or Chamacoco or Chamicuro or Chayahuita or Cocama or "Culina" or Culino or Cubeo or Cuiba or "Cuiva" or Cumanagoto or Curripaco or "Deni" or Desano or Embera or Guarani or Guajajara or "Guana" or Guanano or Guarayo or Guarayu or Guahibo or Guajiro or Guambiano or Guanano or Guayabero or Guarequena or Guinao or "Guana" or Gayon or Guahibo or Hixkaryana or Huachipairi or Huambisa or Huarayo or Lauanaua or Ikpeng or Ingariko or Irantxe or Itonama or Inapari or Iquito or Isconahua or Jumana or Japreria or Jirajara or Juruti or Jaqaru or Jebero or Kadiweu or Kaingang or Kamayura or Karaja or Karipuna or "Kariri" or Katukina or Kaxarari or Kayabi or Kayapo or "Kuikuro alapalo" or Kulina or "Kaiwa" or Kallawaya or "Kogui" or "Kuna" or Kaweskar or "Lule" or Macuna or Maipure or Mapuche or Mataco or Mocovi or Machinere or Machinerev or Machiguenga or Macushi or Macuna or "Madi" or Malayo or Mamainde or Manao or Mandauaca or Mandawaka or Mapidian or Mapuche or Mapidian or Maquiritare or Maquiritari or Maragua or Marawan or Mariate or Marubo or Mastanahua or Mataco or Matipuhy or "Matis" or "Matses" or Mawakua or Mawakwa or Maxakali or Mehinaku or Miranha or Moronawa or Munduruku or Movima or Muellama or Muinane or Mapoyo or "Mashco Piro" or Muniche or Nambikwara or Nocaman or Nuquini or Nomatsiguenga or Nanti or Ocaina or Omagua or Orejon or "Opon" or Pacahuara or "Paez" or Paicone or Palicur or Panare or "Pano" or "Paresi" or Paumari or "Pemon" or Pilaga or Puelche or Pauna or Pauserna or Piapoco or Piraha or Piratapuyo or Pisabo or Piaroa or "Pijao" or Piratapuyo or Paraujano or Pemon or Pemono or Piapoco or Puinave or Patamona or Poyanawa or Puinave or Puquina or Quechua or Quichua or Retuara or Resigaro or Reyesano or Sabanes or "Saliba" or Saluma or Sarave or Secoya or Selknam or "Sensi" or Shaninawa or Shapra or Sharanahua or Shebayo or Shiwiar or Shikiana or Sikiana or Siriono or Sinsiga or "Siona" or Suruwaha or Tacano or Tamanaco or Tiahuanaco or Tariano or Tehuelche or Tariano or Tatuyo or "Tembe" or "Terena" or Telembi or Ticuna or Ticuna or Tiriyo or Tiwanaku or Tiwanaku or "Torom" or "Totoro" or Tsimane or Tuberao or "Tucano" or Tunebo or Tuxinawa or Tuyuca or Uainuma or Urarina or Vilela or Waimaha or Waiampi or Waiwai or Wapishana or Waraiku or Warekena or Waura or Wayampi or Wayana or Wirina or Waimaha or Waunana or "Wiwa" or "Warao" or Wayuu or Witoto or Xavante or Xipaya or Xiriana or Xokleng or Yabaana or Yaminawa or Yaminahua or Yaruma or Yawalapiti or Yuracare or Yabarana or Yavitero or "Yine" or Yamana or Yaghan or Yucuna or Yurumangui or Yukpa or Yanesha or Yoranahua or Yagua or Yaminahua or Zaparo or Zamuco or "Trio Indians" or "More Indians" or "Bare Indians").ti,ab,id. | 1302 |
| 50 | (("Inga" or "Maca" or "Leco" or "Mojo" or "Uro" or "Maco" or Lengua or "Toba" or "Zoe" or "Ona" or "Catio" or "Passe" or "Bari" or "Awa" or "Bora" or "Bara" or "Remo" or "Pano" or "Sape") adj3 (Indians or Indian or Indigenous or Amerindian* or Aborigin* or people or peoples or elder or elders or grandmother* or grandfather* or parent* or women or men or woman or man or child* or youth or youths or baby or babies or tribe or tribes or tribal or shaman* or native or patient*)).ti,ab,id. | 56 |
| 51 | or/49-50 [Indigenous, South America] | 1357 |
| 52 | or/1-51 [Indigenous, Global] | 115383 |
| 53 | smoking cessation/ | 15837 |
| 54 | electronic cigarettes/ or smoking prevention/ or tobacco control/ or vaping/ | 4212 |
| 55 | ((smok* or tobacco* or cigar* or Vape? or vaping or e-cigarette? or e-liquid? or e-cig? or ecig? or eliquid? or ecigarette? or juul or nicotine) adj5 (quit* or ceas* or cessation* or stop* or suspend* or desist* or end* or break* or cutoff* or reduct* or reduce? or reducing or termin* or discontinu* or abstin* or dehabituat* or de-habituat*)).ti,ab,id. | 27484 |
| 56 | (pharmaco* adj2 (smok* or tobacco* or cigar* or Vape? or vaping or e-cigarette? or e-liquid? or e-cig? or ecig? or eliquid? or ecigarette? or juul or nicotine)).ti,ab,id. | 590 |
| 57 | (Nicorette* or bupropion* or vareniclin* or nortriptylin* or clonidin* or chantix* or champix* or wellbutrin* or well-butrin* or Zyntabac* or Quomen* or Zyban* or Amfebutamon* or Cytisine* or baptitoxin* or cytisiniclin* or Polacrilex* or sophorin*).ti,ab,id. | 6811 |
| 58 | (nicotine adj2 (gum* or lozenge* or patch* or spray* or tablet* or sublingual* or sub-lingual* or inhal* or strip? or microtab* or replace*)).ti,ab,id. | 3244 |
| 59 | ((smok* or tobacco* or cigar* or Vape? or vaping or e-cigarette? or e-liquid? or e-cig? or ecig? or eliquid? or ecigarette? or juul or nicotine) adj2 (intervention* or treatment* or program* or therap*)).ti,ab,id. | 11284 |
| 60 | bupropion/ | 1095 |
| 61 | clonidine/ | 1406 |
| 62 | or/53-61 | 38238 |
| 63 | 52 and 62 | 929 |
| 64 | limit 63 to up=20240101-20250528 | 79 |
| 65 | limit 63 to yr="2024 -Current" | 58 |
| 66 | 64 or 65 | 84 |

## S5. Bibliography of Indigenous Peoples in North America Search

| Database: Bibliography of Indigenous Peoples in North America Search  Platform: EBSCO  Date Searched: May 28, 2025 | | | | |
| --- | --- | --- | --- | --- |
| **#** | **Query** | **Limiters/Expanders** | **Last Run Via** | **Results** |
| S4 | S1 AND S2 | Limiters - Publication Type: Academic Journal Expanders - Apply related words; Apply equivalent subjects Search modes - Proximity | Interface - EBSCOhost Research Databases Search Screen - Advanced Search Database - Bibliography of Indigenous Peoples in North America | 280  Wasn’t able to reliably limit by date so exported the lot |
| S3 | S1 AND S2 | Expanders - Apply related words; Apply equivalent subjects Search modes - Proximity | Interface - EBSCOhost Research Databases Search Screen - Advanced Search Database - Bibliography of Indigenous Peoples in North America | 475 |
| S2 | TI ( quit* or ceas* or cessation* or stop* or suspend* or desist* or end* or break* or cutoff* or reduct* or reduce* or reducing or termin* or discontinu* or abstin* or dehabituat* or de-habituat* or intervention* or treatment* or program* or therap* ) OR AB ( quit* or ceas* or cessation* or stop* or suspend* or desist* or end* or break* or cutoff* or reduct* or reduce* or reducing or termin* or discontinu* or abstin* or dehabituat* or de-habituat* or intervention* or treatment* or program* or therap* ) | Expanders - Apply related words; Apply equivalent subjects Search modes - Proximity | Interface - EBSCOhost Research Databases Search Screen - Advanced Search Database - Bibliography of Indigenous Peoples in North America | 34,530 |
| S1 | TI ( smok* or tobacco* or cigar* or Vape* or vaping or e-cigarette* or ecigarette* or juul or nicotine or Nicorette* or bupropion* or vareniclin* or nortriptylin* or clonidin* or chantix* or champix* or wellbutrin* or well-butrin* or Zyntabac* or Quomen* or Zyban* or Amfebutamon* or Cytisine* or baptitoxin* or cytisiniclin* or Polacrilex* or sophorin* ) OR AB ( smok* or tobacco* or cigar* or Vape* or vaping or e-cigarette* or ecigarette* or juul or nicotine or Nicorette* or bupropion* or vareniclin* or nortriptylin* or clonidin* or chantix* or champix* or wellbutrin* or well-butrin* or Zyntabac* or Quomen* or Zyban* or Amfebutamon* or Cytisine* or baptitoxin* or cytisiniclin* or Polacrilex* or sophorin* ) | Expanders - Apply related words; Apply equivalent subjects Search modes - Proximity | Interface - EBSCOhost Research Databases Search Screen - Advanced Search Database - Bibliography of Indigenous Peoples in North America | 2,364 |

## Table 6. Global Health Search

| Database: Global Health  Platform: EBSCO  Date Searched: May 28, 2025 | | | | |
| --- | --- | --- | --- | --- |
| **#** | **Query** | **Limiters/Expanders** | **Last Run Via** | **Results** |
| S12 | S3 AND S10 | Limiters - Publication Year: 20240101-20251231 Expanders - Apply related words; Apply equivalent subjects Search modes - Proximity | Interface - EBSCOhost Research Databases Search Screen - Advanced Search Database - Global Health | 52 |
| S11 | S3 AND S10 | Expanders - Apply related words; Apply equivalent subjects Search modes - Proximity | Interface - EBSCOhost Research Databases Search Screen - Advanced Search Database - Global Health | 828 |
| S10 | S4 OR S5 OR S6 OR S7 OR S8 OR S9 | Expanders - Apply related words; Apply equivalent subjects Search modes - Proximity | Interface - EBSCOhost Research Databases Search Screen - Advanced Search Database - Global Health | 27,531 |
| S9 | TI ( ((smok* or tobacco* or cigar* or Vape* or vaping or e-cigarette* or e-liquid* or e-cig* or ecig* or eliquid* or ecigarette* or juul or nicotine) N2 (intervention* or treatment* or program* or therap*)) ) OR AB ( ((smok* or tobacco* or cigar* or Vape* or vaping or e-cigarette* or e-liquid* or e-cig* or ecig* or eliquid* or ecigarette* or juul or nicotine) N2 (intervention* or treatment* or program* or therap*)) ) | Expanders - Apply related words; Apply equivalent subjects Search modes - Proximity | Interface - EBSCOhost Research Databases Search Screen - Advanced Search Database - Global Health | 8,549 |
| S8 | TI ( (nicotine N2 (gum* or lozenge* or patch* or spray* or tablet* or sublingual* or sub-lingual* or inhal* or strip# or microtab* or replace*)) ) OR AB ( (nicotine N2 (gum* or lozenge* or patch* or spray* or tablet* or sublingual* or sub-lingual* or inhal* or strip# or microtab* or replace*)) ) | Expanders - Apply related words; Apply equivalent subjects Search modes - Proximity | Interface - EBSCOhost Research Databases Search Screen - Advanced Search Database - Global Health | 1,460 |
| S7 | TI ( (Nicorette* or bupropion* or vareniclin* or nortriptylin* or clonidin* or chantix* or champix* or wellbutrin* or well-butrin* or Zyntabac* or Quomen* or Zyban* or Amfebutamon* or Cytisine* or baptitoxin* or cytisiniclin* or Polacrilex* or sophorin*) ) OR AB ( (Nicorette* or bupropion* or vareniclin* or nortriptylin* or clonidin* or chantix* or champix* or wellbutrin* or well-butrin* or Zyntabac* or Quomen* or Zyban* or Amfebutamon* or Cytisine* or baptitoxin* or cytisiniclin* or Polacrilex* or sophorin*) ) | Expanders - Apply related words; Apply equivalent subjects Search modes - Proximity | Interface - EBSCOhost Research Databases Search Screen - Advanced Search Database - Global Health | 1,354 |
| S6 | TI ( (pharmaco* N2 (smok* or tobacco* or cigar* or Vape* or vaping or e-cigarette* or e-liquid* or e-cig# or ecig# or eliquid* or ecigarette* or juul or nicotine)) ) OR AB ( (pharmaco* N2 (smok* or tobacco* or cigar* or Vape* or vaping or e-cigarette* or e-liquid* or e-cig# or ecig# or eliquid* or ecigarette* or juul or nicotine)) ) | Expanders - Apply related words; Apply equivalent subjects Search modes - Proximity | Interface - EBSCOhost Research Databases Search Screen - Advanced Search Database - Global Health | 403 |
| S5 | TI ( ((smok* or tobacco* or cigar* or Vape* or vaping or e-cigarette* or e-liquid* or e-cig# or ecig# or eliquid# or ecigarette# or juul or nicotine) N5 (quit* or ceas* or cessation* or stop* or suspend* or desist* or end* or break* or cutoff* or reduct* or reduce* or reducing or termin* or discontinu* or abstin* or dehabituat* or de-habituat*)) ) OR AB ( ((smok* or tobacco* or cigar* or Vape* or vaping or e-cigarette* or e-liquid* or e-cig# or ecig# or eliquid# or ecigarette# or juul or nicotine) N5 (quit* or ceas* or cessation* or stop* or suspend* or desist* or end* or break* or cutoff* or reduct* or reduce* or reducing or termin* or discontinu* or abstin* or dehabituat* or de-habituat*)) ) | Expanders - Apply related words; Apply equivalent subjects Search modes - Proximity | Interface - EBSCOhost Research Databases Search Screen - Advanced Search Database - Global Health | 22,995 |
| S4 | DE "tobacco control" OR DE "smoking cessation" | Expanders - Apply related words; Apply equivalent subjects Search modes - Proximity | Interface - EBSCOhost Research Databases Search Screen - Advanced Search Database - Global Health | 9,768 |
| S3 | S1 OR S2 | Expanders - Apply related words; Apply equivalent subjects Search modes - Proximity | Interface - EBSCOhost Research Databases Search Screen - Advanced Search Database - Global Health | 83,712 |
| S2 | TI ( (Aboriginal* or aborigine* or aborigen* or amerindian* or amerindio* or indian* or indigene* or indigenous* or indigena* or nativo or originarios or tribe* or tribal*) ) OR AB ( (Aboriginal* or aborigine* or aborigen* or amerindian* or amerindio* or indian* or indigene* or indigenous* or indigena* or nativo or originarios or tribe* or tribal*) ) | Expanders - Apply related words; Apply equivalent subjects Search modes - Proximity | Interface - EBSCOhost Research Databases Search Screen - Advanced Search Database - Global Health | 81,506 |
| S1 | DE "indigenous people" OR DE "aborigines" OR DE "Inuit" OR DE "Native Americans" OR DE "Pacific Islanders" | Expanders - Apply related words; Apply equivalent subjects Search modes - Proximity | Interface - EBSCOhost Research Databases Search Screen - Advanced Search Database - Global Health | 6,824 |

## Table 7. Informit Indigenous Collection Search

| Database: Informit Indigenous Collection  Platform: https://search.informit.org/  Date Searched: September 3, 2024 | |
| --- | --- |
| Search | Results |
| Title:smok* OR Title:tobacco* OR Title:cigar* OR Title:vape? OR Title:vaping OR Title:"e-cigarette" OR Title:"e-cigarettes" OR Title:"e-cig" OR Title:ecigarette* OR Title:juul OR Title:nicotine OR Title:nicorette* OR Title:bupropion* OR Title:vareniclin* OR Title:nortriptylin* OR Title:clonidin* OR Title:chantix* OR Title:champix* OR Title:wellbutrin* OR Title:well-butrin* OR Title:Zyntabac* OR Title:Quomen* OR Title:Zyban* OR Title:Amfebutamon* OR Title:Cytisine* OR Title:baptitoxin* OR Title:cytisiniclin* OR Title:Polacrilex* OR Title:sophorin* OR Abstract:smok* OR Abstract:tobacco* OR Abstract:cigar* OR Abstract:vape? OR Abstract:vaping OR Abstract:"e-cigarette" OR Abstract:"e-cigarettes" OR Abstract:"e-cig" OR Abstract:ecigarette* OR Abstract:juul OR Abstract:nicotine OR Abstract:nicorette* OR Abstract:bupropion* OR Abstract:vareniclin* OR Abstract:nortriptylin* OR Abstract:clonidin* OR Abstract:chantix* OR Abstract:champix* OR Abstract:wellbutrin* OR Abstract:well-butrin* OR Abstract:Zyntabac* OR Abstract:Quomen* OR Abstract:Zyban* OR Abstract:Amfebutamon* OR Abstract:Cytisine* OR Abstract:baptitoxin* OR Abstract:cytisiniclin* OR Abstract:Polacrilex* OR Abstract:sophorin* | 190 |
